# Are Major a Posteriori Dietary Patterns Reproducible in the Italian Population? A Systematic Review and Quantitative Assessment

**DOI:** 10.1016/j.advnut.2023.100165

**Published:** 2023-12-23

**Authors:** Rachele Bianco, Michela C Speciani, Maria Parpinel, Matteo Tesi, Monica Ferraroni, Valeria Edefonti

**Affiliations:** 1 Department of Medicine (DMED), Università degli Studi di Udine, Udine, Italy; 2 Branch of Medical Statistics, Biometry and Epidemiology “G. A. Maccacaro,” Department of Clinical Sciences and Community Health, Università degli Studi di Milano, Milano, Italy; 3 Fondazione IRCCS Ca’ Granda Ospedale Maggiore Policlinico, Milano, Italy

**Keywords:** congruence coefficient, cross-study reproducibility of dietary patterns, a posteriori dietary patterns, factor analysis, generalizability of dietary patterns, Italy, principal component analysis, similarity of dietary patterns across studies, systematic review

## Abstract

Although a posteriori dietary patterns (DPs) naturally reflect actual dietary behavior in a population, their specificity limits generalizability. Among other issues, the absence of a standardized approach to analysis have further hindered discovery of genuinely reproducible DPs across studies from the same/similar populations. A systematic review on a posteriori DPs from principal component analysis or exploratory factor analysis (EFA) across study populations from Italy provides the basis to explore assessment and drivers of DP reproducibility in a case study of epidemiological interest. First to our knowledge, we carried out a qualitative (i.e., similarity plots built on text descriptions) and quantitative (i.e., congruence coefficients, CCs) assessment of DP reproducibility. The 52 selected articles were published in 2001–2022 and represented dietary habits in 1965–2022 from 70% of the Italian regions; children/adolescents, pregnancy/breastfeeding women, and elderly were considered in 15 articles. The included studies mainly derived EFA-based DPs on food groups from food frequency questionnaires and were of “good quality” according to standard scales. Based on text descriptions, the 186 identified DPs were collapsed into 113 (69 food-based and 44 nutrient-based) apparently different DPs (39.3% reduction), later summarized along with the 3 “Mmixed-Salad/Vegetable-based Patterns,” “Pasta-and-Meat-oriented/Starchy Patterns,” and “Ddairy Products” and “Ssweets/Animal-based Patterns” groups, by matching similar food-based and nutrient-based groups of collapsed DPs. Based on CCs (215 CCs, 68 DPs, 18 articles using the same input lists), all pairs of DPs showing the same/similar names were at least “fairly similar” and ∼81% were “equivalent.” The 30 “equivalent” DPs ended up into 6 genuinely different DPs (80% reduction) that targeted fruits and (raw) vegetables, pasta and meat combined, and cheese and deli meats. Such reduction reflects the same study design, list of input variables, and DP identification method followed across articles from the same groups. This review was registered at PROSPERO as CRD42022341037.

**Statement of significance**

This is the first systematic review collecting evidence on Italian dietary patterns derived from principal component or exploratory factor analysis. The systematic review provides the basis for a qualitative and quantitative assessment of reproducibility of Italian dietary patterns, as based on text descriptions and congruence coefficients, respectively. We found that Italian dietary patterns based on fruit and (raw) vegetables, pasta and meat combined, and cheese and deli meats are reproducible across studies, although more rigorous statistical approaches may allow a better identification of reproducible dietary patterns and related causes. The established evidence base may inform dietary pattern identification in the Italian population and more generally future research on dietary pattern reproducibility across studies within the same country.

## Introduction

Following the dietary pattern (DP) approach, multiple related dietary components (food items, food groups, or nutrients) are synthesized into combined variables reflecting key dietary profiles in a population [1,2], while overcoming well-known multiple comparison issues [3].

A posteriori DPs [3] are defined by using multivariate statistical methods (i.e., principal component analysis [PCA], exploratory factor analysis [EFA], and cluster analysis [4]) and are therefore advantageous in naturally reflecting actual dietary behavior in a population and related study- or population-specific context (e.g., geography/climate, socioeconomic status, food supply, ethnic background, culinary tradition) [5]. However, their specificity limits generalizability, especially when compared with the a priori (i.e., comparing subjects’ diet against evidence-based benchmark diets) option [6].

The absence of a standardized approach to analysis (e.g., definition of input variables and their preprocessing, DP identification method, and DP labeling), poor information reporting, and subjective DP labeling (based on supposed similarities with previously published DPs) have further limited fair comparisons among sets of a posteriori DPs [7] and still hindered discovery of genuinely reproducible DPs across studies from the same or similar populations [8,9] (i.e., cross-study reproducibility [7,10]).

A few pioneering [11,12] and more recent [[13], [14], [15], [16], [17], [18], [19]] articles have explored either qualitatively or quantitatively the cross-study reproducibility of DPs derived from PCA or EFA, which are by far the most commonly derived a posteriori DPs in nutritional epidemiology [3]. Following a qualitative approach, the assessment of cross-study reproducibility emerged from a narrative synthesis based on text description and/or visual inspection of loadings of potentially similar DPs. Congruence coefficients (CCs) between factor loadings and correlation coefficients between factor scores have been also used to quantitatively evaluate reproducibility of apparently similar study-specific DPs [13,15,16]. Independently of the different cut-offs used, the CC has proved to be an effective measure of reproducibility for PCA/EFA-based DPs across studies [13,15,16]. Additionally, the potential effectiveness of more rigorous statistical approaches has been under investigation [17].

The Italian diet is traditionally recognized as a variant of the Mediterranean diet characterized by the abundance of fruit and vegetables, wheat, legumes, and olive oil [20,21]. However, per capita weekly consumption data revealed that typical Mediterranean-style foods have been consumed less than expected in 2019 [22]. A Life Cycle Inventory analysis suggested that, while intakes of milk/yogurt and legumes were in line with the Mediterranean nutritional model, as estimated by using current dietary reference values [23], fruits, vegetables, pasta, bread, and extra-virgin olive oil showed lower (24%–51%, depending on the food item/group) intakes, compensated by higher (78%–918%) intakes of meat, and higher (580%) intakes of sugar, sweets, snacks, or alcohol-free beverages [22]. While waiting for novel findings from official nation-wide food consumption surveys [24]—the most recent one dating back to the 2005–2006 “Istituto Nazionale di Ricerca per gli Alimenti e la Nutrizione-Studio sui Consumi Alimentari in Italia” (INRAN-SCAI) [25]—a systematic review of the otherwise scattered scientific evidence on Italian dietary behavior may contribute to fill in this gap, by summarizing recent evidence in the light of the old one. As recent country-specific dietary guidelines recognized the effective use of DPs as their first evidence base [2,26], a systematic review on all and more recent DPs may contribute to inform future research on DP identification in the Italian population and the development of the next Italian dietary guidelines.

Within the movement supporting reproducible research in science [27], the current article builds on the first 2 systematic reviews on reproducibility and validity of PCA/EFA-based DPs in nutritional epidemiology [7,10] and explores the cross-study reproducibility of PCA/EFA-based DPs in a case study of epidemiological interest, which is Italy. In detail, first to our knowledge, we systematically collected existing evidence on PCA/EFA-based DPs identified in Italian free-living individuals, with a focus on the DP identification process and its consistency across included articles. We also investigated DP cross-study reproducibility, to assess whether major DPs are consistently identified within Italy, by proposing a:

1. qualitative assessment of reproducibility of all available and most recently identified DPs, as based on similarity plots built on original text descriptions and factor loadings;
2. quantitative assessment of reproducibility of subsets of DPs, as based on the CCs applied on the same list of input variables.

As a third research aim, we compared the results from the qualitative and quantitative assessments of DP reproducibility, to identify possible drivers of agreement and discrepancies. This not only informs DP assessment in the Italian population but also future research utilizing a posteriori DP identification methods. A companion article will examine whether the identified DPs, grouped according to their reproducibility, are consistently related to disease outcomes, determinants, or correlates of interest, if any, as described in the original articles included in this systematic review.

## Methods

This systematic review was conducted referring to the Preferred Reporting Items for Systematic Reviews and Meta-Analyses (PRISMA) 2020 guidelines [28]. The review protocol was registered in the International Prospective Register of Systematic Reviews database (registration no: CRD42022341037).

### Eligibility criteria

Articles were considered eligible for inclusion if they (1) were (original) full-texts articles in peer-reviewed journals; (2) enrolled human subjects living in Italy; (3) identified DPs based on PCA and/or EFA (indicated as PCA-based, EFA-based, or PCA/EFA-based DPs in the following) on dietary data, independently of any additional analysis on health outcomes, determinants, or correlates. Articles were excluded if (1) they did not provide original data, or they were case reports, in vitro and in vivo animal studies, conference abstracts or posters; (2) the reference population lived outside Italy or, in international studies, it was not possible to distinguish the Italian-specific DPs, which are of interest in the current review; (3) results concerned single nutrients, single food items, or single food groups; (4) the term DP was used to identify dietary attitudes, perceptions, or patterns of meals; (5) DPs were identified using the a priori approach, the mixed-type approach, or the a posteriori approach but not following PCA or EFA; (6) PCA or EFA were applied on dietary behaviors; and (7) PCA or EFA were applied on lifestyle variables, including diet, to derive lifestyle patterns (details in Supplementary Methods). No restrictions were imposed on year of publication, population characteristics, or participants’ health status.

### Search strategy

An electronic literature search was conducted in parallel by 2 authors (RB and MT) on December 21, 2022 using 3 electronic databases: MEDLINE/PubMed, Embase, and Cochrane (CENTRAL and Reviews). The search strategy used both keywords and controlled vocabulary terms around the fields of “dietary patterns,” “factor analysis,” “principal component analysis,” and “Italy.” No language filters were used. No reference was made to potential health outcomes, determinants, or correlates of interest, as far as PCA/EFA-based DPs were identifiable in Italy. Details on strings were provided in Supplementary Methods. We used the EndNote 20 software program (Thomson Reuters, New York, NY, USA) for the electronic management of the review process.

### Article selection

After duplicates were removed, titles and abstracts of the remaining articles were screened for eligibility. Subsequently, all eligible full-text articles were retrieved, screened, and included in the systematic review when appropriate. The reference lists of the articles identified during this process were also examined by hand search to further identify potentially relevant articles. Each of the previous steps was carried out in parallel by 2 authors (RB and MT); any disagreements between reviewers were resolved by discussion and consensus with a third investigator (VE).

### Data extraction

Using a predefined Excel spreadsheet, data extraction was performed independently by 2 investigators (RB and MT). Data extraction was checked by other 2 investigators (VE and MS) and a third one (MF) was involved in resolving any potential disagreement. Information extracted from each study included the following: (1) general characteristics of the studies; (2) study design; (3) dietary assessment tool used; (4) DP identification method; (5) number of DPs, proportion of variance explained, name, and composition; (6) statistical methods used to relate the identified DPs to disease outcomes/determinants/correlates, and (7) main results on the relationship between identified DPs and disease outcomes/determinants/correlates (corresponding to those statistical models adjusted for all the available confounders, if models were fitted).

The current article is focused on the description of the PCA/EFA-based DPs identified in Italy, with a focus on their identification process and on their potential cross-study reproducibility. A companion review will be focused on the relationship between identified DPs and disease outcomes/determinants/correlates, by providing details on the statistical methods used to assess this relationship.

### Assessment of study quality

For each aticle that met the inclusion criteria, study quality was independently evaluated by 2 reviewers (RB and MS) by using the Quality Assessment Tools from the National Institutes of Health National Heart, Lung, and Blood Institute [29]. Any disagreements were solved by discussion and consensus with a third reviewer’s grade (VE). Involved researchers used the available study rating tools on the range of items provided by each tool (range: 0–14 for cohort, cross-sectional studies, or trials; 0–12 for case–control studies) to judge each study quality [29]. To better identify mid–high-quality studies, we added an extra category, “very good,” to the originally suggested “poor,” “fair,” and “good” [29]. We categorized total scores into 4 levels in such a way that ≥25% (corresponding to 3 points) of item’s positive answers were included in any category. Owing to the lack of previous evidence on reproducibility of DPs in Italy, we chose not to exclude studies based on their quality. Therefore, all the retrieved studies were considered in the analyses.

### Narrative synthesis and qualitative and quantitative assessments of reproducibility of DPs

We first performed a narrative synthesis of the findings from the included studies in terms of study design, population characteristics, dietary assessment tool, DP identification methods, and text description of the identified DPs. Second, we performed a qualitative assessment of the reproducibility of all available DPs, as based on similarity plots built on original text descriptions and factor loadings, when available; we referred to factor loadings to assess the relative importance of dominant food groups or nutrients, in case of very rich descriptions of DPs. Third, we performed a quantitative assessment of reproducibility of DPs, as based on the CCs calculated on the same lists of input variables. The CC (-1≤CC≤1) is the preferred index for measuring similarity of PCA/EFA-based DPs [30,31]. In the absence of any recent and reliable information on Italian DPs, we followed a more conservative approach than the most similar systematic review on PCA/EFA-based DPs from Japan [13]. In detail, we opted for (1) calculating CCs over smaller but more comparable groups of articles sharing the same list of input variables (i.e., either nutrients or food groups), to avoid extra subjectivity in defining a common input list and potential artifacts possibly deriving from imputation of new loadings based on the original ones [13]; (2) adopting a higher cut-off (CC: 0.85 vs. 0.80 [13]) for “fair similarity” of DPs, thereby a 0.85≤CC≤0.94 indicates “fair similarity” [15,16] in our application; (3) adopting a specific cut-off (CC: 0.95) for “equivalence” of DPs, thereby a CC ≥ 0.95 indicates “equivalence” [15,16]; and (4) evaluating similarity of DP pairs over the entire CC distribution and not only on the median [13]. The quantitative assessment of DP reproducibility was conducted with the R software [32] and its package “psych” [33]. When needed, corresponding authors were contacted (twice, 15 days apart per protocol) to provide or confirm information on PCA/EFA loadings that allowed to calculate CCs. Finally, we carried out a sensitivity analysis (including both a qualitative and quantitative assessment of DP reproducibility) on the most recently identified DPs (i.e., those based on dietary information collected at least in part over 2013–2022), to assess if any shifting from typical Mediterranean-style habits can be tracked.

## Results

### Article selection process

**FIGURE 1:**
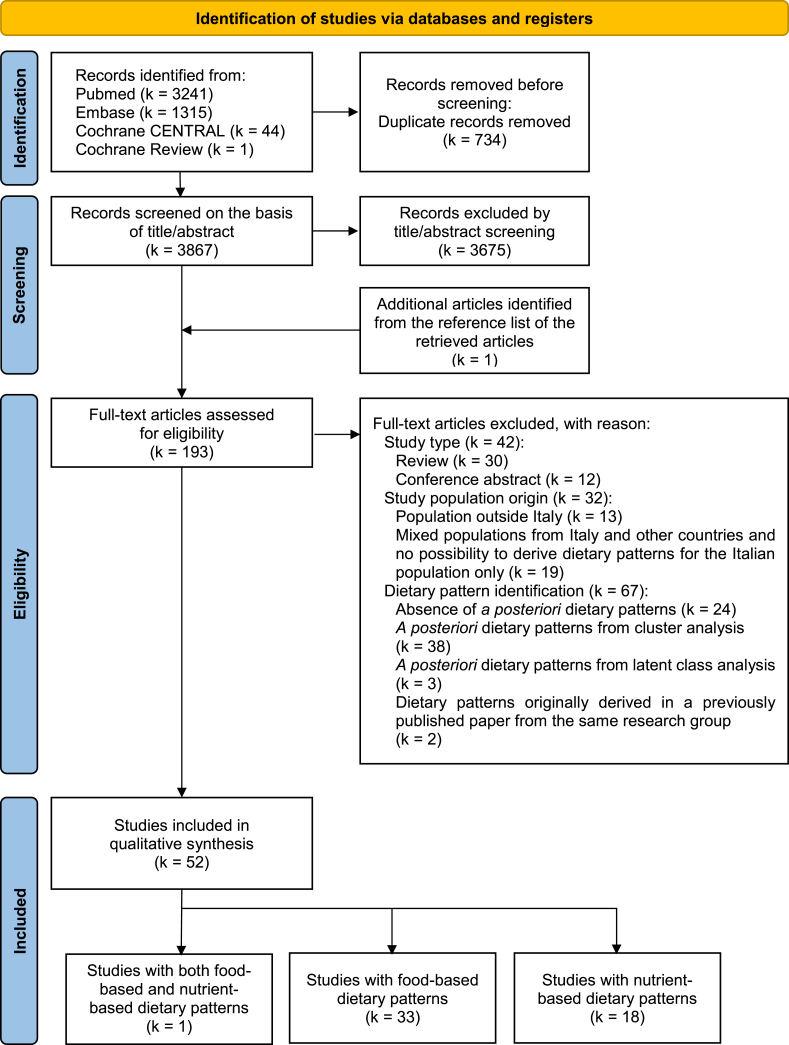
Flow diagram of the study selection process [28]. EMBASE, Excerpta Medica Database; PRISMA, Preferred Reporting Items for Systematic Reviews and Meta-Analyses.

Figure 1 shows the PRISMA flowchart of the article selection process. The electronic literature search detected 4601 records. After 734 duplicates were removed and 3675 records were excluded by title/abstract screening, 193 full-text articles (including 1 article from the reference lists of the retrieved articles) were considered eligible for a detailed analysis. Of these, 52 (all in English language) remained after exclusion criteria were applied and were summarized in the current review [11,12,[34], [35], [36], [37], [38], [39], [40], [41], [42], [43], [44], [45], [46], [47], [48], [49], [50], [51], [52], [53], [54], [55], [56], [57], [58], [59], [60], [61], [62], [63], [64], [65], [66], [67], [68], [69], [70], [71], [72], [73], [74], [75], [76], [77], [78], [79], [80], [81], [82], [83]]. Reasons for exclusion are described in Figure 1.

### Quality assessment of the identified articles

Among the selected articles, 7 (13.5%) [57,[59], [60], [61], [62],^74^,^82^] were based on studies of “very good” quality, 35 (67.3%) [12,[34], [35], [36], [37], [38], [39], [40], [41], [42], [43], [44], [45], [46], [47], [48], [49], [50], [51], [52], [53], [54], [55], [56],^58^,^65^,^67^,^68^,^70^,^73^,^76^,^77^,^80^,^81^,^83^] on studies of “good” quality, 8 (15.4%) [63,64,66,69,71,72,78,79] on studies of “fair” quality, and 2 (3.8%) [11,75] on studies of “poor” quality; the 2 studies of “poor” quality did not refer to any outcome and therefore lost 6 over 14 points (Supplemental Table 1 for details on the single studies). Across the different quality assessment tools, sample size justification was the item that received the highest number of “No” replies (Supplemental Figure 1).

### Main characteristics of the included studies

**FIGURE 2:**
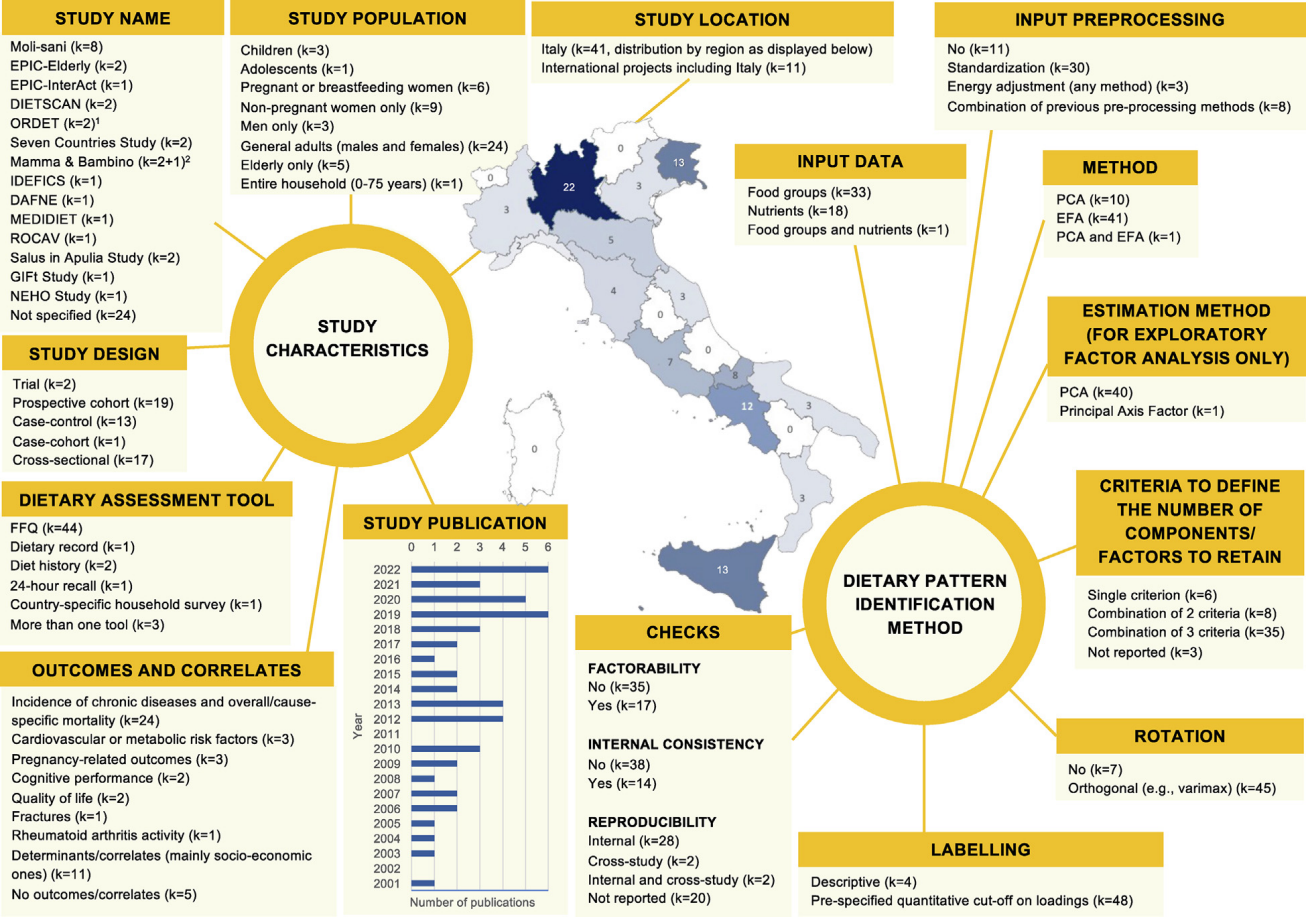
General characteristics of the studies included in the systematic review and main steps in the dietary pattern identification process: a summary of findings from the systematic review. DAFNE, Data Food Networking; DIETSCAN, Dietary Patterns and Cancer; EFA, exploratory factor analysis; EPIC, European Prospective Investigation into Cancer and Nutrition; FFQ, food frequency questionnaire; GIFt, gestational intake of food toward healthy outcomes; IDEFICS, Identification and prevention of Dietary- and lifestyle-induced health EFfects In Children and infantS; NEHO, Neonatal Environment and Health Outcomes; ORDET, Ormoni e Dieta nell'Eziologia del Tumore della Mammella; PCA, Principal Component Analysis; ROCAV, Risk Of Cardiovascular diseases and abdominal aortic Aneurysm in Varese. ^1^The DIETSCAN project included one Italian cohort – the ORDET one – which recruited women only and it was therefore classified as “nonpregnant women only” instead of “general adults (males and females)”.^2^The Mamma & Bambino birth cohort was also pooled together with MAMI-MED in another study (Magnano San-Lio et al. [65]).

Figure 2 summarizes study design, dietary assessment tools, disease outcomes/determinants/correlates of interest, and the DP identification process used in the 52 selected articles (Supplemental Table 2 for additional details).

Target populations covered 14 out of the 20 Italian regions, with Lombardy, Friuli Venezia Giulia, Sicily, and Campania being the most represented regions. Selected articles were published between 2001 [81] and 2022 [47,65,66,75,82,83], with 21% published by 2009 and 79% from 2010 onward. Eleven articles (21%) referred to international studies including Italy [11,47,[56], [57], [58],^61^,^62^,^66^,^70^,^71^,^80^], 15 (29%) were based on Italian multicentric studies [34,[36], [37], [38], [39], [40], [41], [42], [43], [44], [45],^65^,^72^,^82^,^83^], and 26 (50%) recruited participants from single centers/geographic areas [35,46,[48], [49], [50], [51], [52], [53], [54], [55],^59^,^60^,^63^,^64^,[67], [68], [69],[73], [74], [75], [76], [77], [78], [79], [80], [81]]. Several articles were based on the same studies, including (but not limited to) those from the (Italian) Moli-sani [[48], [49], [50], [51], [52], [53], [54], [55]] (8 articles), Mamma & Bambino [[63], [64], [65]] (3 articles), Salus in Apulia Study [74,75] (2 articles), and “Ormoni e Dieta nell'Eziologia del Tumore della Mammella” (ORDET) [59,60] (2 articles), as well as the international programs “Dietary Patterns and Cancer” (DIETSCAN) [11,12] (2 articles where the ORDET cohort represented Italy), “European Prospective Investigation into Cancer and Nutrition” (EPIC)-Elderly [56,57] (2 articles), EPIC-InterAct [58] (1 article), and the “Seven Countries Study” [61,62] (2 articles). The most frequent study design was the prospective cohort [11,12,47,54,56,57,[59], [60], [61], [62], [63], [64], [65],^70^,^74^,^79^,^80^,^82^,^83^] (19 articles, of which 8 [11,47,56,63,65,70,79,83] performed cross-sectional analyses only), followed by the cross-sectional design [46,[48], [49], [50], [51], [52], [53],^55^,[66], [67], [68], [69],[71], [72], [73],^75^,^76^] (17 articles), and by the case–control [[34], [35], [36], [37], [38], [39], [40], [41], [42], [43], [44], [45],^81^] (13 articles) one. As to the target population, 24 articles included general (males and females) adults [[35], [36], [37], [38], [39], [40],[43], [44], [45], [46],[48], [49], [50], [51], [52], [53], [54], [55],^58^,^73^,^74^,^77^,^78^,^81^], 3 articles included men only [41,61,62], 15 included women only [11,12,34,42,59,60,[63], [64], [65], [66], [67], [68],^72^,^82^,^83^], of which 6 were based on pregnant or breastfeeding women [[63], [64], [65],^72^,^82^,^83^]. In addition, elderly [56,57,75,79,80], children/adolescents [47,69,70,76] and the entire household (0–75 years) [71] were considered in another 10 articles (Figure 2).

### Dietary assessment

Italian dietary habits were generally assessed once, at recruitment, with a single tool and they referred to the 1965 [61,62] to 2022 [65] period. With few exceptions [47,71,78], interviewer-administered [[34], [35], [36], [37], [38], [39], [40], [41], [42], [43], [44], [45], [46],[63], [64], [65], [66], [67], [68],^70^,^72^,^77^,^83^] or self-administered [11,12,[48], [49], [50], [51], [52], [53], [54], [55],^59^,^60^,^69^,[73], [74], [75]] food frequency questionnaires (FFQs) were mostly adopted (Supplemental Table 2 and Figure 2). In most studies, the FFQ reference period was either 1 [11,12,[56], [57], [58], [59], [60],[73], [74], [75],^77^,^81^] or 2 [[34], [35], [36], [37], [38], [39], [40], [41], [42], [43], [44], [45]] years; shorter reference periods were mainly related to recordings during pregnancy or lactation [[63], [64], [65],^72^,^82^,^83^]. With the exception of 2 articles [81,83], most of the FFQs were reported to be reproducible and/or valid, or based on previously validated tools. The number of food items investigated in the FFQs ranged from 31 [74] to 217 [56,57] (median: 95 items), with 48% of the FFQs showing >100 items (Supplemental Table 2).

### DP identification

DPs were identified on nutrients in 18 articles [[34], [35], [36], [37], [38], [39], [40], [41], [42], [43], [44], [45], [46], [47],^72^,^77^,^78^,^81^] and on food groups in 33 articles [11,12,[48], [49], [50], [51], [52], [53], [54], [55], [56], [57], [58], [59], [60], [61], [62], [63], [64], [65], [66], [67], [68], [69], [70], [71],[73], [74], [75], [76],^79^,^82^,^83^], with one article using both input data [80] (Figure 2 and Supplemental Table 3). Selected nutrients ranged from 10 [80] to 37 [47] (median: 28 nutrients) and selected food groups from 8 [80] to 57 [56,57] (median: 37 food groups) (Supplemental Table 3). Among included articles, 10 performed PCA and 41 performed EFA, whereas one article [62] performed both; EFA was generally applied using the PCA method. Most analyses preprocessed input data, especially by using standardization. The number of components/factors to retain was mostly defined through a combination of 2 (8 articles) or 3 (35 articles) criteria including: eigenvalue>1 or 2, Scree-plot construction (or percentage of variance explained), or component/factor interpretability. Varimax rotation was the preferred orthogonal rotation method, and it was applied in 45 articles (Figure 2). Most articles adopted a quantitative labeling of components/factors, referring to cut-offs ranging from 0.1 [75] to 0.63 [[34], [35], [36], [37], [38], [39], [40], [41], [42], [43], [44], [45], [46],^72^,^77^] in absolute value (median: 0.30, Supplemental Table 3).

Checks on matrix factorability prior to EFA were proposed in 17 articles (∼33%) [[35], [36], [37], [38], [39], [40], [41], [42], [43], [44], [45], [46], [47],^72^,[76], [77], [78]], of which 15 conducted by the same research team [[35], [36], [37], [38], [39], [40], [41], [42], [43], [44], [45], [46], [47],^72^,^77^]. Similarly, the same research group assessed internal consistency of DPs with Cronbach’s alpha in 14 articles (∼27%) [[35], [36], [37], [38], [39], [40], [41], [42], [43], [44], [45], [46], [47],^72^]. Finally, 28 articles (∼54%) assessed internal reproducibility of DPs by using different statistical approaches [10]. Although most of them referred to the split-half approach, different EFA estimation procedures or factor score calculations were also compared [[34], [35], [36], [37], [38], [39], [40], [41], [42], [43], [44], [45], [46], [47], [48], [49], [50], [51], [52], [53], [54], [55],^61^,^62^,^64^,^67^,^72^]. Two articles assessed cross-study reproducibility [7] of DPs [70,71], whereas another 2 [11,58] assessed both internal and cross-study reproducibility of DPs (Figure 2 and Supplemental Table 3).

The number of DPs described in each article ranged from 2 to 6 (food-based DPs: 2–6, nutrient-based DPs: 3–5), with a median of 4 DPs per article. When reported, the percentage of total variance explained by the retained components/factors varied from 6.6% (3 factors, 46 food groups) [55] to 82% (2 factors, 17 food groups) [61,62], with a median percentage of 45.5%. Seventeen articles showed percentages over 75%, with most of them (15 articles) identifying nutrient-based DPs (Supplemental Table 3).

### Qualitative assessment of DP reproducibility: original descriptions

Overall, 186 DPs were identified across all the included articles (food-based DPs: 102; nutrient-based DPs: 84). Except for 15 DPs without any label, the matching of the remaining 171 DPs on original names allowed to identify DPs named as “Vitamins and Fiber” (14 articles, from case–control studies on diet and cancer), “Starch-rich” (13 articles, from case–control studies on diet and cancer), “Animal Products” (13 articles, from case–control studies on diet and cancer), “Prudent” (11 articles, from a research group from Sicily, EPIC-Elderly, ORDET, and “Neonatal Environment and Health Outcomes” birth cohort), “Pasta and Meat” (10 articles, from Moli-sani and EPIC-Elderly), “Western” (9 articles, from a research group from Sicily, ORDET, and “Risk Of Cardiovascular diseases and abdominal aortic Aneurysm in Varese”), “Eggs and Sweets” (8 articles, from Moli-sani), “Olive Oil and Vegetables” (8 articles, from Moli-sani), as well as “Animal Unsaturated Fatty Acids” (“AUFA”) and “Vegetable Unsaturated Fatty Acids” (“VUFA”) (7 and 5 articles, respectively, from case–control studies on diet and cancer) (Supplemental Table 3).

**FIGURE 3:**
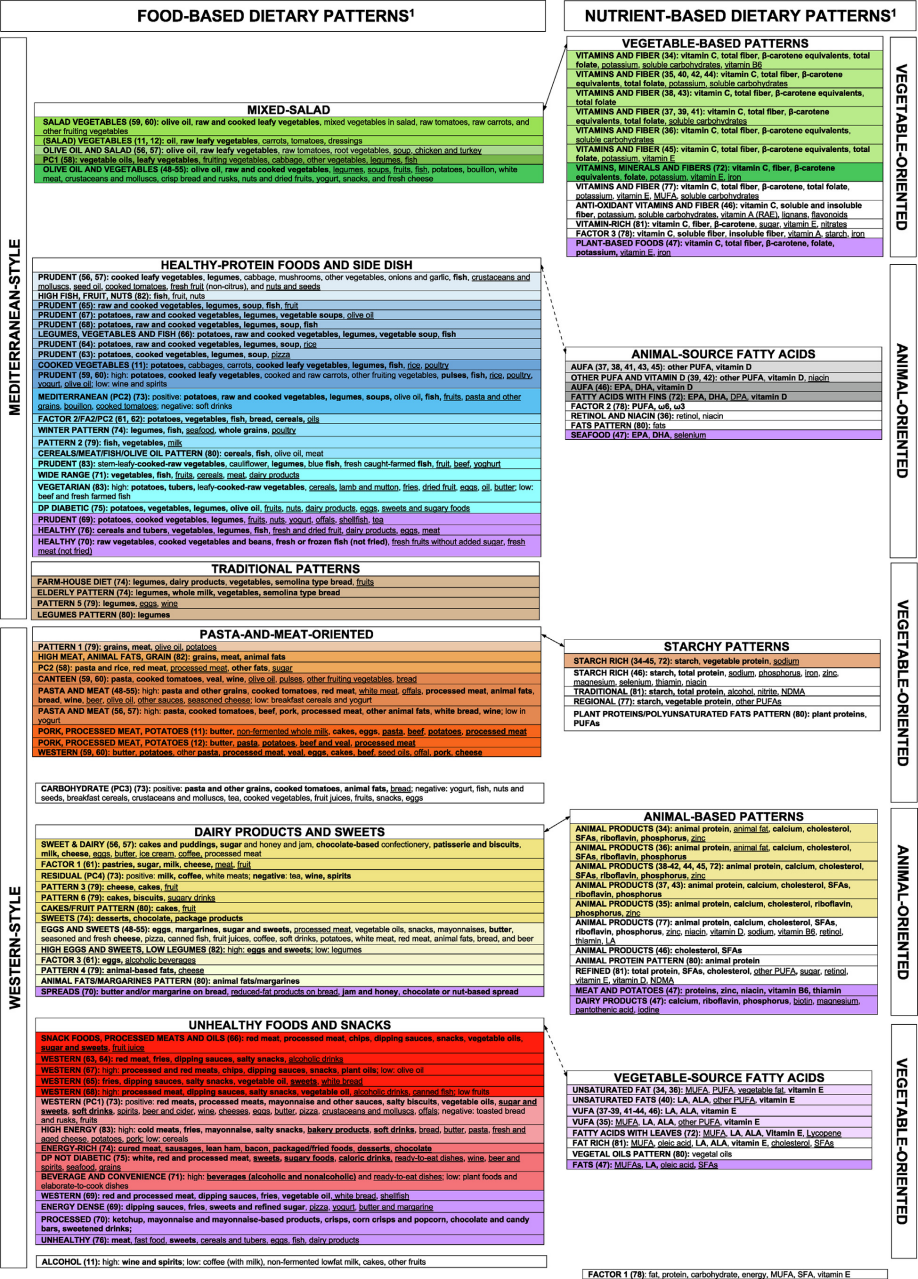
Qualitative assessment of reproducibility for all the available dietary patterns: dietary patterns identified using principal component analysis or exploratory factor analysis in Italy from 1965 to 2022, in groups based on original text descriptions and loadings. ALA, α-linolenic acid; AUFA, animal unsaturated fatty acids; DHA, docosahexaenoic acid; DP, dietary pattern; DPA, docosapentaenoic acid; EPA, eicosapentaenoic acid; FA, factor analysis (factor name from original articles); LA, linoleic acid; NDMA, N-nitrosodimethylamine; PC, principal component (analysis) (principal component names from original articles); RAE, retinol activity equivalent; VUFA, vegetable unsaturated fatty acids. ^1^Dietary patterns that look similar (based on original loadings and text description) were placed one close to the other and consistently indicated with the same color code. When dietary patterns were virtually identical, we synthetized them as one cell. Dietary patterns left in white were too far from the others to be indicated with a color code. Variants of the same color indicate different subgroups of dietary patterns within the same group, with loadings showing modest but nutritionally relevant differences across color-specific subgroups. Results were separately displayed for food-based (left) and nutrient-based (right) patterns and for adults and children/adolescents (consistently indicated in violet). Food-based and nutrient-based patterns were juxtaposed based on correlation coefficients between nutrient-based dietary patterns and selected food groups, as provided in most of the original articles. Arrows linking the different groups indicate stronger (solid line) and weaker (dashed line) similarities between food-based and nutrient-based dietary patterns.

To compensate for subjective DP labeling, we referred to text descriptions and loadings in original articles to collapse in Figure 3 the 186 identified DPs (expressed with original names) into 113 apparently different DPs (39.3% total reduction), of which 69 were food-based and 44 were nutrient-based DPs.

#### Food-based DPs

We organized the 69 food-based DPs into “Mediterranean-style” and “Western-style” macro-areas (Figure 3). The Mediterranean-style macro-area included 3 different groups of DPs that we defined as “Mixed-Salad,” “Healthy-Protein Foods and Side Dish,” and “Traditional” DPs. The “Mixed-Salad” group (in green) included DPs based on olive oil, raw (and sometimes cooked) vegetables (DPs named “Salad Vegetables”) [11,12,59,60], with additional presence of legumes and fish [58], soup and turkey in the EPIC-Elderly study [56,57], and further inclusion of fruits and potatoes in the Moli-sani study [[48], [49], [50], [51], [52], [53], [54], [55]]. The “Healthy-Protein Foods and Side Dish” group (in blue) included DPs based on the presence of at least one source of healthy proteins (i.e., fish, poultry, nuts, and/or legumes) and a side dish represented mainly by cooked vegetables [56,57,65,70,79], potatoes and/or grains [74,80], or a combination of them [11,[59], [60], [61], [62], [63], [64],[66], [67], [68], [69],^73^,^76^]. In addition, fruit loaded high on a “Prudent”-like DP in 5 articles [56,57,65,73,82], one of which just expressed fish, nuts, and fruit [82]. Four DPs of the “Healthy-Protein Foods and Side Dish” group presented a wider range of components in adults [71,75,83] or children/adolescents [69,70,76]. The DPs included in the “Traditional” group (in brown) characterized elderly populations from Apulia and Calabria (southern Italy) and shared consumption of legumes [80], integrated with semolina-type bread, dairy products, and other vegetables [74], or eggs and wine [79].

The “Western-style” macro-area included the “Pasta-and-Meat oriented,” “Dairy Products and Sweets*,*” and “Unhealthy Foods and Snacks” groups of patterns. The “Pasta-and-Meat-oriented” group (in orange) included DPs loading high on grains (e.g., pasta and/or rice), (red) meat, and animal fats [11,12,[48], [49], [50], [51], [52], [53], [54], [55], [56], [57], [58], [59], [60],^82^]. Additional dominant food groups were cooked tomatoes, (white) bread, and wine [[48], [49], [50], [51], [52], [53], [54], [55], [56], [57],^59^,^60^]. The “Dairy Products and Sweets” group (in yellow) included DPs loading high on sweets [74,79,80], dairy products [73,79] or spreads [80], and eggs [61], or a combination of them [[48], [49], [50], [51], [52], [53], [54], [55], [56], [57],^61^,^70^,^79^,^82^]. The “Unhealthy Foods and Snacks” group (in red) included DPs loading high on processed foods, like snacks or salty snacks, dipping sauces, deli meats (including cold cut, cured meat, sausages, bacon, lean ham), desserts or sugary/soft drinks, and ready-to-eat dishes, as identified in adults (including pregnant women), or children/adolescents [[63], [64], [65], [66], [67], [68], [69], [70], [71],[73], [74], [75], [76],^83^]. In addition, 5 DPs of this group also included alcoholic beverages [63,64,68,71,73,75].

Although alcoholic beverages have been previously identified in the “Traditional,” “Pasta-and-Meat-oriented,” and “Unhealthy Foods and Snacks” groups as consumed at mealtime, one article identified an “Alcohol” DP alone, likely because the DIETSCAN project provided DPs based on a parallel analysis of international studies [11].

#### Nutrient-based DPs

Apart from a single DP [78] representing the overall diet, we organized the 44 nutrient-based DPs into the “Animal-oriented” and “Vegetable-oriented” macro-areas (Figure 3). The “Animal-oriented” macro-area included 2 different groups of DPs, “Animal-based Patterns” and “Animal-source Fatty Acids”. Within the “Animal-based Patterns” group (in yellow) the “Animal Products” DP was characterized in most articles by animal protein, calcium, cholesterol, SFAs, riboflavin, phosphorus, and zinc [[38], [39], [40], [41], [42],^44^,^45^,^72^]; based on a different classification of fats, 2 articles [34,36] additionally showed animal fat in the “Animal Products” DP. Although 3 DPs showed a richer DP composition in adults (“Animal Products” DP [77]) and children (“Dairy products” and “Meat and Potatoes” DPs[47]), another 2 were poorly characterized [46,80]. Finally, the “Refined" DP [81] suggested shifting toward more processed foods.

Within the “Animal-source Fatty Acids” group (in gray), most DPs from the same research group were labeled “AUFA” and were mainly characterized by vitamin D and other PUFAs [37,38,41,43,45]. In another 3 articles, eicosapentaenoic acid, docosahexaenoic acid [46,47], and/or docosapentaenoic acid [72], omega-3 and omega-6 [78] were found as dominant nutrients, due to a different classification of fats. Three additional articles also included niacin among the “AUFA” DP-based dominant nutrients [36,39,42], of which one included niacin and retinol only [36].

The “Vegetable-oriented” macro-area included 3 different groups of DPs that we defined as “Vegetable-based Patterns,” “Vegetable-source Fatty Acids,” and “Starchy Patterns”. Within the “Vegetable-based Patterns” group (in green), most DPs from the same research group were labeled “Vitamins and Fiber” and were all characterized by vitamin C, total fiber, and β-carotene equivalents; additional dominant nutrients were total folate, potassium, vitamin B6, vitamin E, and soluble carbohydrates, alone or in combination [[34], [35], [36], [37], [38], [39], [40], [41], [42], [43], [44], [45]]. In other articles, additional dominant nutrients included MUFAs, iron, nitrates, lignans, vitamin A, flavonoids, starch, or a combination of some of them [46,47,72,77,78,81].

Within the “Vegetable-source Fatty Acids” group (in lilac), most DPs from the same research group were labeled “VUFA” and were all characterized by linoleic acid, α-linolenic acid, and vitamin E [[37], [38], [39],[41], [42], [43], [44],^46^]. Pregnant women additionally loaded high on MUFAs and lycopene [72]. A different classification of fats allowed to identify vegetable fat as an additional dominant nutrient in 2 articles from the same research group [34,36]. The joint presence of vegetable and animal sources of fatty acids mainly characterized the “Unsaturated Fats” [40], the “VUFA” [35], and the“Fat-rich” [81] DPs in adults, as well as the “Fats” DP in children [47]. Finally, 1 article identified a “Fats Pattern” but did not provide further specification on the type of fats; however, the presence of a “Vegetal Oil Pattern” in the same article allowed us to interpret the former “Fats Pattern” as belonging to the “Animal-source Fatty Acids” group and the latter “Vegetal Oil Pattern” as belonging to the “Vegetable-source Fatty Acids” group [80].

Within the "“Starchy Patterns” group (in orange), the “Starch-rich” DPs from the same research group were all characterized by starch, vegetable protein, and sodium [[34], [35], [36], [37], [38], [39], [40], [41], [42], [43], [44], [45],^72^]; additional nutrients included various minerals and vitamins [46], as well as PUFAs/other PUFAs [77,80]. Similarly, the “Traditional” DP from Tuscany included nitrites, alcohol, and N-nitrosodimethylamine, together with starch and total protein [81].

#### Food-based and nutrient-based DPs: an overall picture

Based on correlation coefficients between nutrient-based DPs and selected food groups provided in the original articles [[38], [39], [40], [41], [42], [43], [44], [45]], we identified similarities between the following groups of nutrient-based and food-based DPs (Figure 3, solid line):

1. “Mixed-Salad” and “Vegetable-based Patterns,”
2. “Pasta-and-Meat-oriented” and “Starchy Patterns,”
3. “Dairy Products and Sweets” and “Animal-based Patterns.”

Similarities were less clear between the “Healthy-Protein Foods and Side Dish” and “Animal-source Fatty Acids” groups and the “Unhealthy Foods and Snacks” and “Vegetable-source Fatty Acids groups”, respectively (Figure 3, dashed line). As the “AUFA” DP showed fish together with red meat, liver, unspecified seed oil, olive oil, and eggs (ordered according to frequency), it generally showed a healthy source of proteins, but no side dishes. Food groups correlated with the “VUFA” DP included unspecified seed oils, together with red meat, specified seed oil, and olive oil, which might target fried foods potentially present in the “Unhealthy Foods and Snacks” group, but other relevant food groups (i.e., processed meat, soft drinks, or sugar and candies) did not show up.

### Quantitative assessment of DP reproducibility: congruence coefficients

Globally, 215 CCs were calculated across 68 apparently similar DPs identified in the 18 articles that used the same lists of input variables (68/186=36.6% reduction in DPs, 18/52=∼35% selected articles whose details are provided in Supplemental Table 4). All CCs suggested “fair similarity” of DPs and 80.9% suggested DP “equivalence.” When collapsing DPs based on “fair similarity,” the 68 DPs under evaluation ended up into 13 genuinely different DPs, 6 of which were due to the different input data lists used in the Moli-sani study [[49], [50], [51],[53], [54], [55]]; when collapsing DPs based on “equivalence,” 30 DPs ended up into 6 genuinely different DPs (with 2 “Pasta and Meat” DPs from the Moli-sani study [[49], [50], [51],[53], [54], [55]]) (80% total reduction).

**FIGURE 4:**
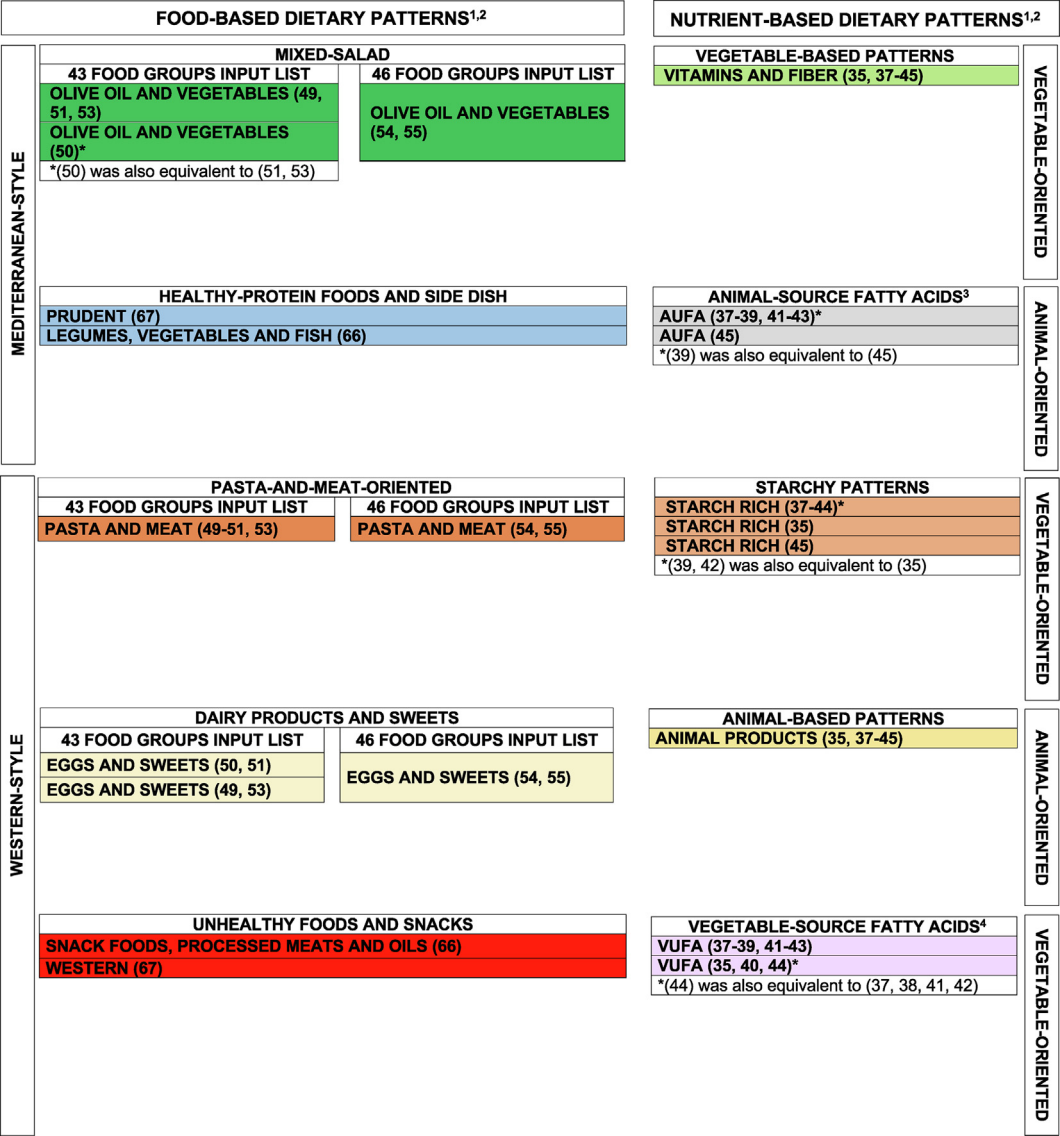
Quantitative assessment of reproducibility for those dietary patterns identified on the same list of input variables: dietary patterns identified using principal component analysis or exploratory factor analysis in Italy from 1991 to 2017 and evaluated to be equivalent. AUFA, animal unsaturated fatty acids; VUFA, vegetable unsaturated fatty acids. ^1^Each cell included only equivalent dietary patterns, as expressed by all available congruence coefficients. ^2^Congruence coefficients were computed within groups of dietary patterns presenting the same list of input variables [[49], [50], [51],^53^] and, separately, [54,55] for the “Mixed-Salad,” the “Pasta-and-Meat-oriented,” and the “Dairy Products and Sweets” groups, due to different lists of food groups; [66,67] for the “Unhealthy Foods and Snacks” and the “Healthy-Protein Foods and Side Dish” groups; [35,[37], [38], [39], [40], [41], [42], [43], [44], [45]] for the “Vegetable-based Patterns” group; [[37], [38], [39],[41], [42], [43],^45^] for the “Animal-source Fatty Acids” group; [35,[37], [38], [39], [40], [41], [42], [43], [44], [45]] for the "Starchy Patterns” group; [35,[37], [38], [39], [40], [41], [42], [43], [44], [45]] for the “Animal-based Patterns” group; [35,[37], [38], [39], [40], [41], [42], [43], [44]] for the “Vegetable-source Fatty Acids” group. Results were separately displayed for food-based (left) and nutrient-based (right) patterns. Food-based and nutrient-based patterns were juxtaposed based on correlation coefficients between nutrient-based dietary patterns and selected food groups, as provided in most of the original articles. ^3^Among the dietary patterns here named “AUFA”, the 2 from [39,42] were originally named “Other PUFAs and Vitamin D.” ^4^Among the dietary patterns here named “VUFA”, the one from [40] was originally named "Unsaturated Fats.”

Separate summary statistics of CCs by research group and DP labels are provided in Table 1 [11,12,35,[37], [38], [39], [40], [41], [42], [43], [44], [45],[49], [50], [51],[53], [54], [55],^59^,^60^,[63], [64], [65], [66], [67], [68],^74^,^75^,^79^,^80^] and corresponding “equivalent” DPs are summarized in Figure 4 [35,[37], [38], [39], [40], [41], [42], [43], [44], [45],[49], [50], [51],[53], [54], [55],^66^,^67^]. Within the 10 available multicentric case–control studies on diet and cancer at different sites [35,[37], [38], [39], [40], [41], [42], [43], [44], [45]], the “Animal Products” and the “Vitamins and Fiber” DPs consistently showed “equivalence,” as the minimum of the CC distributions already reached 0.95; the “AUFA” DP showed “equivalence” in ≥75% of its CCs (first quartile of CCs: 0.96), whereas the “Starch-rich” and the “VUFA” DPs were “equivalent” in ≥50% of the corresponding CCs (median of CCs: 0.98 and 0.96, respectively). Within the 6 available articles from the Moli-sani study [[49], [50], [51],[53], [54], [55]], the “Olive Oil and Vegetables,” “Eggs and Sweets,” and “Pasta and Meat” DPs were separately compared across 4 articles considering 43 food groups [[49], [50], [51],^53^] and 2 articles considering 46 food groups [54,55]. In the former comparison, the “Pasta and Meat” DP consistently showed “equivalence” (minimum CCs ≥ 0.95), the “Olive Oil and Vegetables” DP showed “equivalence” in 75% of its CCs (first quartile of CCs = 0.95) and the “Eggs and Sweets” DP showed “equivalence” in ≥25% of the corresponding CCs (third quartile of CCs = 0.98) [[49], [50], [51],^53^]. In the latter comparison, the same 3 pairs of DPs were equivalent [54,55] (see Supplemental Tables 5 and 6 for details). Within 2 companion articles of a research group from Sicily [66,67], pairs of similar DPs did not reach “equivalence” (Table 1 and Figure 4) [11,12,35,[37], [38], [39], [40], [41], [42], [43], [44], [45],[49], [50], [51],[53], [54], [55],^59^,^60^,[63], [64], [65], [66], [67], [68],^74^,^75^,^79^,^80^].

**TABLE 1:** Quantitative assessment of dietary pattern reproducibility for those dietary patterns identified on the same list of input variables: summary statistics on congruence coefficients^1^ between loadings of pairs of apparently similar dietary patterns^2^

Multicentric case–control studies on diet and cancer at several sites, articles presenting the same list of 28 nutrients as input variables [35,[37], [38], [39], [40], [41], [42], [43], [44], [45]]						
Nutrient-based dietary pattern	Number involved articles	Minimum	First quartile	Median	Third quartile	Maximum

Animal Products	10	0.95	0.98	0.99	0.99	1.00
Vitamins and Fiber	10	0.95	0.97	0.98	0.99	0.99
Starch-rich	10	0.88	0.93	0.98	0.99	1.00
Animal Unsaturated Fatty Acids (AUFA)^3^	7	0.91	0.96	0.97	0.98	0.99
Vegetable Unsaturated Fatty Acids (VUFA)^4^	9	0.88	0.93	0.96	0.97	0.99

Moli-sani study, articles presenting the same list of 43 food groups as input variables [[49], [50], [51],^53^]						

Food-based dietary pattern	Number involved articles	Minimum	First quartile	Median	Third quartile	Maximum

Olive Oil and Vegetables	4	0.94	0.95	0.95	0.98	1.00
Pasta and Meat	4	0.97	0.97	0.98	0.99	1.00
Eggs and Sweets	4	0.92	0.93	0.94	0.98	1.00
Moli-sani study, articles presenting the same list of 46 food groups as input variables [54,55]						
Food-based dietary pattern	Number involved articles			Congruence coefficient		

Olive Oil and Vegetables	2			0.98		
Pasta and Meat	2			0.98		
Eggs and Sweets	2			0.97		
Research group from Sicily, articles presenting the same list of 39 food groups as input variables [66,67]						
Food-based dietary pattern	Number involved articles			Congruence coefficient		

Snack foods, processed meats and oils/Western^5^	2			0.91		
Legumes, vegetables and fish/Prudent	2			0.90		

1 Congruence coefficients range between 0 and 1 (in absolute value), with values between 0.85 and 0.94 indicating fair similarity, and values ≥0.95 indicating equivalence of corresponding dietary patterns.

2 Dietary patterns identified within the ORDET cohort [11,12,59,60] were not compared one to the other because the full list of factor loadings was not available anymore from the corresponding authors, we were in contact with; similarly, dietary patterns identified in most articles from the research group from Sicily [[63], [64], [65],^68^] were not compared because the full list of factor loadings was not available anymore from the corresponding authors; upon contact with the corresponding author, we were able to confirm that dietary patterns obtained from 2 articles from Calabria [79,80] were identified by using exactly the same study population and therefore the comparison is meaningless; finally, dietary patterns obtained from 2 articles from the Salus in Apulia Study [74,75] were not compared because the number of food groups was different across articles.

3 Three articles [35,40,44] did not contribute to the congruence coefficient-based analyses as the Animal Unsaturated Fatty Acids dietary pattern was not identified in those articles; among the dietary patterns here named Animal Unsaturated Fatty Acids, the 2 from [39,42] were originally named Other PUFAs and Vitamin D.

4 One article [45] did not contribute to the congruence coefficient-based analyses as the Vegetable Unsaturated Fatty Acids dietary pattern was not identified in that article; among the dietary patterns here named Vegetable Unsaturated Fatty Acids, the one from [40] was originally named Unsaturated Fats.

5 Minor inconsistencies were detected in the names of the food groups across the 2 articles. In the current analysis, vegetable oils in [66] was considered equivalent to plant oil in [67]; sugar, sweets in [66] was considered equivalent to sweet and processed sugar in [67].

When integrating corresponding nutrient- and food-based DPs, the “Vitamins and Fiber”/“Olive Oil and Vegetables” DPs were equivalent in 98% of the CCs, the “Animal Products”/“Eggs and Sweets” DPs in 92% of the CCs, and the “Pasta and Meat”/“Starch-rich” DPs in 71% of the CCs.

### Qualitative and quantitative assessment of DP reproducibility: a comparison

In the comparison between FIGURE 3, FIGURE 4 [35,[37], [38], [39], [40], [41], [42], [43], [44], [45],[49], [50], [51],[53], [54], [55],^66^,^67^], we observed that:

1. For the “Animal Products” and “Vitamins and Fiber” DPs, different cells in Figure 3 were indicated to be all “equivalent” based on CCs, so nuances in Figure 3 did not end up into genuinely different DPs in Figure 4 [35,[37], [38], [39], [40], [41], [42], [43], [44], [45],[49], [50], [51],[53], [54], [55],^66^,^67^];
2. For the “AUFA” DP, ∼76% of CCs pointed to “equivalence,” with all the “fairly similar” evaluations related to the bladder cancer study [45]; however, the 2 cells identified in Figure 3 did not reflect this finding, as the “AUFA” DP for bladder cancer was not separate from all the other DPs and “equivalence” was identified between bladder and esophageal cancers [39,45], whose DPs, however, were in 2 different cells;
3. For the “Starch-rich” DP, the same 3 dominant nutrients—represented with 1 cell in Figure 3—ended up into an “equivalent” DP in 67% of the CCs only, with all “fairly similar” evaluations given by gastric and bladder cancer studies [35,45];
4. For the “VUFA” DP, ∼61% of CCs pointed to “equivalence,” with all the “fairly similar” evaluations related to the pancreatic and gastric cancer studies (which also showed “equivalence” between the corresponding “VUFA” DPs); this finding was reflected in part by Figure 3, where gastric- and pancreatic-cancer-related DPs [35,40] were in different cells compared with the other “VUFAs”, but not in the same cell;
5. For the “Pasta and Meat” and “Olive Oil and Vegetables” DPs on both available food-group lists, the DPs presented in Figure 3 were materially confirmed, as all CCs suggested “equivalence,” except for 1 in the “Olive Oil and Vegetables” DP on the 43 food groups [[49], [50], [51],^53^];
6. For the “Eggs and Sweets” DP, the DP presented in Figure 3 was confirmed on the 46 food groups [54,55], but not on the 43 food groups [[49], [50], [51],^53^], where only 33% of CCs suggested “equivalence” between DPs with the same name; most differences were related to the DPs identified for the nutrition knowledge and mass media exposure [50,51] articles, which were, however, “equivalent”;
7. The 2 DPs from the research group from Sicily [66,67] were indicated in different cells in Figure 3 and were consistently indicated as “fairly similar” in Table 1 [11,12,35,[37], [38], [39], [40], [41], [42], [43], [44], [45],[49], [50], [51],[53], [54], [55],^59^,^60^,[63], [64], [65], [66], [67], [68],^74^,^75^,^79^,^80^].

### Sensitivity analysis: qualitative and quantitative assessments of reproducibility for the most recently identified DPs

**FIGURE 5:**
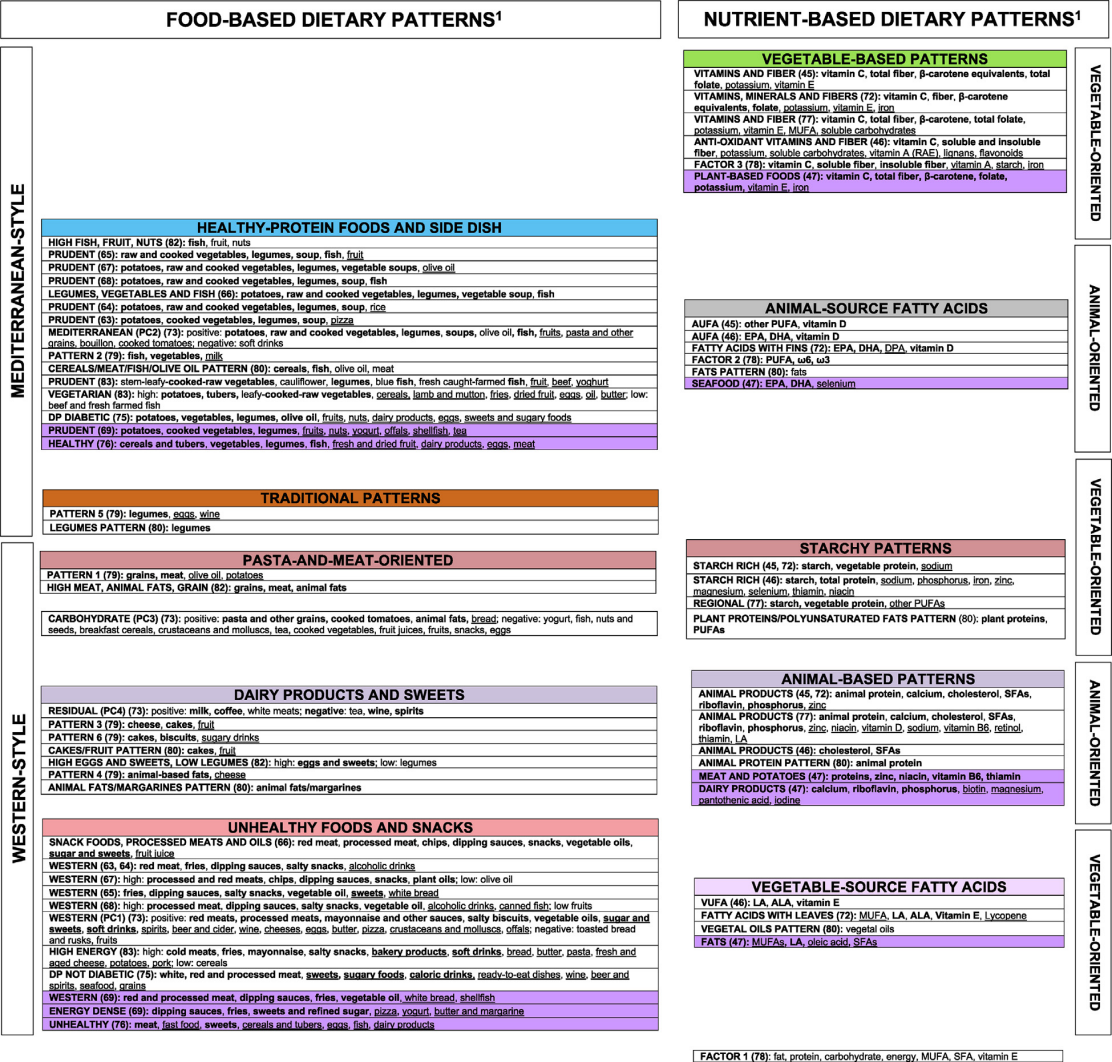
Sensitivity analysis: qualitative assessment of reproducibility for the most recently identified (i.e., latest 10 years of dietary data collection) dietary patterns—dietary patterns identified using principal component analysis or exploratory factor analysis in Italy from 2013 to 2022, in groups based on original text descriptions and loadings. ALA, alpha-linolenic acid; AUFA, animal unsaturated fatty acids; DHA, docosahexaenoic acid; DP, dietary pattern; DPA, docosapentaenoic acid; EPA, eicosapentaenoic acid; LA, linoleic acid; PC, principal component (analysis) (principal component names from original articles); RAE, retinol activity equivalent; SFA, saturated fatty acid(s); VUFA, vegetable unsaturated fatty acids. ^1^Dietary patterns that look similar (based on original loadings and text description) were placed one close to the other. When dietary patterns were virtually identical, we synthetized them as one cell. Results were separately displayed for food-based (left) and nutrient-based (right) patterns and for adults and children/adolescents (consistently indicated in violet). Food-based and nutrient-based patterns were juxtaposed based on correlation coefficients between nutrient-based dietary patterns and selected food groups, as provided in most of the original articles.

Twenty articles identified PCA/EFA-based DPs on dietary habits collected in Italy during 2013 to 2022. Among these, 4 (20%) recruited children, adolescents, or university students [47,69,76,78], 6 (30%) considered pregnant/breastfeeding women [[63], [64], [65],^72^,^82^,^83^] and 3 (15%) nonpregnant women of ∼40 years attending clinical laboratories from Sicily [[66], [67], [68]]; in addition, 4 (20%) recruited elderly [73,75,79,80]. Middle-aged adults of both sexes were available in 3 studies only (15%), of which each sample included at least in part subjects with a disease [45,46,77]. Figure 5 shows the 68 most recently identified DPs collapsed into 65 apparently different DPs (4.4% total reduction), of which 38 were food-based and 27 were nutrient-based DPs. In the comparison between Figures 3 (i.e., all existing DPs) and 5 (i.e., most recently identified ones), the most striking differences that we observed were:

1. The “Mixed-Salad” group was no longer present in Figure 5 (100% reduction);
2. The “Pasta-and-Meat-oriented” group showed a 78% reduction;
3. The “Traditional,” the “Vegetable-source fatty Acids,” and the “Vegetable-based Patterns” groups showed a 50% reduction;
4. The “Starchy Patterns,” the “Unhealthy Foods and Snacks,” and the “Animal-source Fatty Acids” groups showed at most a 25% reduction.

## Discussion

The present systematic review provides a first summary of the evidence on identification methods and reproducibility of PCA/EFA-based DPs across Italian studies. Based on 52 articles published between 2001 and 2022, the included studies collected dietary habits in the 1965–2022 period and mainly derived DPs with EFA applied over food groups obtained from FFQ-based information. Within the qualitative assessment of DP reproducibility by using similarity plots (based on original text descriptions and loadings), we identified similarities across food-based and nutrient-based groups of DPs, i.e., between the “Mixed-Salad” and “Vegetable-based Patterns” groups, between the “Pasta-and-Meat-oriented” and “Starchy Patterns” groups, and between the “Dairy Products and Sweets” and “Animal-based Patterns” groups. Within the quantitative assessment of DP reproducibility by using CCs (215 CCs comparing pairs of DPs among the 68 DPs identified in 18 articles which referred to the same input data lists), pairs of DPs indicated with the same/similar names were all “fairly similar” and ∼81% of them were “equivalent.” Among them, the “Vitamins and Fiber”/“Olive Oil and Vegetables” DPs were equivalent in 98% of the CCs, the “Animal Products”/“Eggs and Sweets” DPs in 92% of the CCs, and the “Pasta and Meat”/“Starch-rich” DPs in 71% of the CCs.

The lack of a standardized approach to DP identification, the subjective labeling of DPs, and a generally poor information reporting have severely limited the ability to genuinely assess reproducibility of a posteriori DPs in different study populations from the same country [9,13,84]. This is especially critical nowadays for Italy, where the most recent nation-wide survey dated back to the INRAN-SCAI 2005–2006 [25]. The current review may provide support to either of these issues, by popularizing the good practice of assessing factorability, internal consistency, and internal reproducibility of identified DPs [10], by highlighting difficulties in using qualitative criteria for DP comparison, and by proposing a quantitative evaluation of reproducibility based on CCs.

Checks on matrix factorability allow to assess if the correlation structure is amenable to PCA/EFA [85]. They are especially useful in food-based PCA/EFA, because the correlation structure is generally weaker. Although they are available in standard statistical software, their use must be increased, to avoid meaningless applications of PCA/EFA. Additional checks on DP internal reproducibility beyond the easiest split-half approach may reassure on their similarity under different statistical options, thus unrevealing the role of subjective decisions in the final PCA/EFA solution [85].

Although DPs are frequently named following a quantitative cut-off applied after rotation, their labeling is still very subjective. In addition, as the label generally needs to be short, often names do not adequately convey to what the underlying principal component/factor is [6]. This was evident in our systematic review, where DPs with the same names did not show such a similar dietary composition, and DPs with similar loadings were given different names. We therefore provided the reader with Figure 3**,** which summarized the 186 identified DPs into 113 apparently different ones**,** based on original text descriptions and loadings. However, Figure 3 is not as effective in synthesizing Italian dietary behavior as one would expect. This is due in part to the need of integrating nutrient-based and food-based DPs in the same picture; although each of the 2 options has its pros and cons (2), matching of food-based and nutrient-based DPs is an extra step of analysis that requires subjective decisions. In addition, within each group, so many likely similar DPs (e.g., those identified by different nuances of the same color) still needs to be somehow summarized, to distinguish true differences from negligible ones or artifacts/noise.

To compensate for these issues, we proposed to quantify with the CCs [14,15,84] similarities between DPs provided in articles that are based on the same list of input variables. In the absence of any recent and reliable information on Italian DPs, we followed the strictest possible approach and provided the reader with benchmark CCs representing the same lists of input variables. In the current systematic review, however, individual research teams did generally adopt the same list of input variables across multiple articles. Therefore, while starting from the same list of variables, we obtained companion study designs, similar inclusion criteria, and dietary assessment tools, a similar preprocessing of input data, and similar DP identification methods. This is what it is reasonable to expect when the same research team develops experience in the application of the same approach over time; however, we could not separate out the contribution of study design and statistical analysis to the cross-study reproducibility of the corresponding DPs.

In this very conservative set-up, we were able to collapse the 68 DPs under evaluation into 13 genuinely different DPs. Although based on ∼35% of included articles only, we believe that the “Vitamins and Fiber/Olive Oil and Vegetables” DPs, the “Animal Products”/“Eggs and sweets” DPs, and the “Pasta and Meat”/“Starch-rich” DPs do effectively summarize the overall Italian dietary behavior expressed in the studies under evaluation in this part of the analysis.

The qualitative assessment added nuances to the quantitative-based representation of the Italian diet. In detail, we identified 3 groups of DPs that we named “Mixed-Salad”/“Vegetable-based Patterns,” “Pasta-and-Meat-oriented”/“Starchy Patterns,” and “Dairy Products and Sweets”/“Animal-based Patterns.” In line with foods typical of the Mediterranean diet, the “Mixed-Salad” or “Vegetable-based Patterns” groups are composed by DPs loading high on raw vegetables and olive oil, with fruit also contributing strongly to the “Vegetable-based Patterns” group. The “Pasta-and-Meat-oriented”/“Starchy Patterns” groups represent the internationally known Italian diet, based on main courses like lasagna, Bolognese pasta, and stuffed pasta; this DP could also encompass pasta/rice eaten at lunch and meat eaten at dinner, together with bread and wine. Finally, the “Dairy Products and Sweets”/“Animal-based Patterns” groups capture the use of cheese, milk, eggs, and sweets, with red and processed meat, butter/margarine, and mayonnaise loading also high on the “Dairy Products and Sweets” group.

Based on 3-day dietary records, the most recent available nation-wide survey INRAN-SCAI 2005–2006 [25] had confirmed results from older surveys that emphasized a large contribution to the overall diet of typical Mediterranean foods, including olive oil to fats, wine to alcoholic beverages, and bread/pasta/pizza to cereals. In 2005–2006, meat was consumed in 99% of the sample, with an alarming average for red meat of ∼100 g/day/capita (raw weight) compared with 418 g/day/capita of fruit and vegetables, in line with Food and Agriculture Organization/World Health Organization recommendations. In line with INRAN-SCAI 2005–2006, recently published consumption trends of available food groups (corrected for waste) over 2000–2017 [86] revealed no important changes in cereals, legumes, pork meat, poultry, eggs, and sugars compared with a relevant decline for animal fat, beef meat, and fruits and vegetables, albeit the last two to a lesser extent. However, while looking at DP reproducibility over recently collected (i.e., last 10 y) dietary data (20 articles), the variety of specific subpopulations under investigation did not allow us to assess whether the trends identified (e.g., the “Mixed-Salad” group is no longer prevalent, the “Pasta-and-Meat-oriented” or the “Traditional” groups are less frequently followed than in past) are generalizable to the overall Italian population. The current sensitivity analysis cannot, therefore, confirm the putative shift of current Italian DPs from more traditional habits, including fruit and raw vegetables, legumes, pasta with meat and tomato sauce, to deli meat, ready-to-eat and/or energy-dense foods.

The current systematic review has strengths and limitations. First, it is based on a nonnegligible number of articles—in line with the systematic review from Japan [13]—and allowed for tracking of Italian dietary habits over a reasonably long time period, with most of the articles covering the last 20 y. Second, it provided graphical summaries of results, synthesizing results on the DP identification process and the qualitative and quantitative assessments of DP reproducibility. Third, being first to our knowledge, we compared qualitative and quantitative evaluations of DP cross-study reproducibility. Among limitations of this systematic review, we acknowledge that it mostly included cross-sectional studies/cross-sectional analyses of cohort studies and case–control studies (73% of the included articles). Moreover, 9 research groups were responsible for ∼83% of articles, and 6 Italian regions, including Sardinia and Trentino-South Tyrol, were not covered by any publication, thus reducing the possibility of identifying nuances in dietary behavior likely useful in defining Italian dietary guidelines. Even though most studies were of “good quality,” reporting of statistical analysis methods and of results was poor in several articles. In the absence of published factor-loading matrices, contacts with the corresponding authors were sometimes unsuccessful, preventing the inclusion of the article in the quantitative assessment of DP reproducibility. Although simple to calculate, CCs look at pairs of DPs; when sets of 5–10 similar DPs are under comparison, this implies evaluating 10–45 CCs and it might therefore be difficult to obtain one clear picture of reproducibility. In addition, we could only apply CCs to distinct lists of nutrients and food groups, thus limiting our ability to provide a global quantitative assessment of DP reproducibility. Finally, although the high CCs obtained did reflect similarities in study design and statistical analysis, we cannot exclude that overlapping of study participants artificially inflated the CCs. In particular, we acknowledge that CCs calculated on the Moli-sani study referred the same original study population, even if the corresponding DPs were identified over the specific subpopulations under investigation in each article and sample sizes generally differed substantially across these articles.

In conclusion, the current systematic review of evidence on 186 PCA/EFA-based DPs identified in Italy confirmed that labeling of DPs is still not performed with sufficient accuracy, even when a quantitative cut-off is followed. Although a degree of subjectivity exists, a qualitative assessment of DP reproducibility, by using graphs built on text descriptions and corresponding loadings, may inform further quantitative assessments performed by using CCs. However, further analyses are needed to better assess why discrepancies, if any, were found between qualitative and quantitative assessments of DP reproducibility. The quantitative assessment of DP reproducibility was carried out following very strict criteria; in particular, we restricted the analysis to articles using the same list of PCA/EFA variables. Although this choice depicts the best-case scenario of consistent study design and analysis, future quantitative assessments of DP reproducibility should include all available articles, to test how much CCs were reduced, when calculated on DPs from independent research groups.

## Acknowledgments

We thank researchers involved in the Moli-sani study (contact person: Bonaccio), the ORDET study (contact persons: Sieri and Sant), the Salus in Apulia Study (contact person: Donghia), as well as the Clinical Nutrition Unit of the University Magna Graecia, Catanzaro (contact persons: Montalcini and Ferro), for providing further information on their original studies. We thank our Administrative Secretary, Barbara Marinelli, for careful checks of information provided in Supplemental Tables 2 and 3. The authors acknowledge the support of the APC central fund of the University of Milan.

### Author contributions

The authors’ responsibilities were as follows—VE and MF: designed overall research plan; RB and MT: screened the data, collected the relevant articles and selected those to be included in the systematic review; RB and MS: extracted the data for the systematic review and prepared corresponding tables, and prepared the figures provided in the manuscript; VE: revised all tables and figures; RB and MS: performed the risk of bias assessment for studies included in the systematic review, and performed the quantitative assessment of dietary pattern reproducibility based on congruence coefficients; VE and MF: provided supervision on statistical issues; MP provided supervision on nutritional issues; VE wrote the article with assistance from RB and MS who especially contributed to the Results section; VE: had primary responsibility for final content; all authors provided critical review of the manuscript and reviewed and approved the final version.

### Conflict of interest

The authors report no conflicts of interest.

### Funding

Valeria Edefonti was supported by the Young Investigator Grant 2020, from Università degli Studi di Milano. Funder had no role in the review.

### Data availability

This systematic review made use of publicly available data from published studies. Therefore, no original data are available for sharing. Template data collection forms, data extracted from included studies, and data and analytic code used for the quantitative assessment of dietary pattern reproducibility using congruence coefficients are available upon request from the corresponding author.

## Footnotes

Appendix A Supplementary data to this article can be found online at https://doi.org/10.1016/j.advnut.2023.100165.

## References

[1] F.B. Hu, Dietary pattern analysis: a new direction in nutritional epidemiology. Curr. Opin. Lipidol. 13(1) (20002) 3–9, 10.1097/00041433-200202000-00002. 11790957

[2] Edefonti V., De Vito R., Parpinel M., Ferraroni M. (2023). Dietary patterns and cancer risk: an overview with focus on methods. The. N. Engl. J. Stat. Data. Sci..

[3] Zhao J., Li Z., Gao Q., Zhao H., Chen S., Huang L. (2021). A review of statistical methods for dietary pattern analysis. Nutr. J..

[4] Gleason P.M., Boushey C.J., Harris J.E., Zoellner J. (2015). Publishing nutrition research: a review of multivariate techniques-part 3: data reduction methods. J. Acad. Nutr. Diet..

[5] Jannasch F., Riordan F., Andersen L.F., Schulze M.B. (2018). Exploratory dietary patterns: a systematic review of methods applied in pan-European studies and of validation studies. Br. J. Nutr..

[6] Schulze M.B., Martinez-Gonzalez M.A., Fung T.T., Lichtenstein A.H., Forouhi N.G. (2018). Food based dietary patterns and chronic disease prevention. BMJ.

[7] Edefonti V., De Vito R., Salvatori A., Bravi F., Patel L., Dalmartello M. (2020). Reproducibility of a posteriori dietary patterns across time and studies: a scoping review. Adv. Nutr..

[8] Krebs-Smith S.M., Subar A.F., Reedy J. (2015). Examining dietary patterns in relation to chronic disease: matching measures and methods to questions of interest. Circulation.

[9] Liese A.D., Krebs-Smith S.M., Subar A.F., George S.M., Harmon B.E., Neuhouser M.L. (2015). The Dietary Patterns Methods Project: synthesis of findings across cohorts and relevance to dietary guidance. J. Nutr..

[10] Edefonti V., De Vito R., Dalmartello M., Patel L., Salvatori A., Ferraroni M. (2020). Reproducibility and validity of a posteriori dietary patterns: a systematic review. Adv. Nutr..

[11] Balder H.F., Virtanen M., Brants H.A., Krogh V., Dixon L.B., Tan F. (2003). Common and country-specific dietary patterns in four European cohort studies. J. Nutr..

[12] Mannisto S., Dixon L.B., Balder H.F., Virtanen M.J., Krogh V., Khani B.R. (2005). Dietary patterns and breast cancer risk: results from three cohort studies in the DIETSCAN project. Cancer. Causes. Control..

[13] Murakami K., Shinozaki N., Fujiwara A., Yuan X., Hashimoto A., Fujihashi H. (2019). A systematic review of principal component analysis-derived dietary patterns in Japanese adults: are major dietary patterns reproducible within a country?. Adv. Nutr..

[14] Bakolis I., Hooper R., Bachert C., Lange B., Haahtela T., Keil T. (2018). Dietary patterns and respiratory health in adults from nine European countries-evidence from the GA2 LEN study. Clin. Exp. Allergy..

[15] Castello A., Buijsse B., Martin M., Ruiz A., Casas A.M., Baena-Canada J.M. (2016). Evaluating the applicability of data-driven dietary patterns to independent samples with a focus on measurement tools for pattern similarity. J. Acad. Nutr. Diet..

[16] Castello A., Lope V., Vioque J., Santamarina C., Pedraz-Pingarron C., Abad S. (2016). Reproducibility of data-driven dietary patterns in two groups of adult Spanish women from different studies. Br. J. Nutr..

[17] De Vito R., Lee Y.C.A., Parpinel M., Serraino D., Olshan A.F., Zevallos J.P. (2019). Shared and study-specific dietary patterns and head and neck cancer risk in an international consortium. Epidemiology.

[18] Edefonti V., Hashibe M., Ambrogi F., Parpinel M., Bravi F., Talamini R. (2012). Nutrient-based dietary patterns and the risk of head and neck cancer: a pooled analysis in the International Head and Neck Cancer Epidemiology consortium. Ann. Oncol..

[19] Moskal A., Pisa P.T., Ferrari P., Byrnes G., Freisling H., Boutron-Ruault M.C. (2014). Nutrient patterns and their food sources in an international study setting: report from the EPIC study. PLOS. ONE..

[20] Trichopoulou A. (2001). Mediterranean diet: the past and the present. Nutr. Metab. Cardiovasc. Dis..

[21] Trichopoulou A., Kouris-Blazos A., Vassilakou T., Gnardellis C., Polychronopoulos E., Venizelos M. (1995). Diet and survival of elderly Greeks: a link to the past. Am. J. Clin. Nutr..

[22] Vinci G., Maddaloni L., Prencipe S.A., Ruggeri M., Di Loreto M.V. (2022). A comparison of the Mediterranean diet and current food patterns in Italy: a life cycle thinking approach for a sustainable consumption. Int. J. Environ. Res. Public. Health..

[23] Scalfi L., Censi L., Marra M., Maffeis C., Pecoraro P., Polito A. (2014).

[24] Turrini A., Le Donne C., Piccinelli R., D’Addezio L., Mistura L., Sette S. (2022). Italian national dietary survey on adult population from 10 up to 74 years old–IV SCAI ADULT: CREA-Consiglio per la ricerca in agricoltura e l’analisi dell’economia agraria-Centro di ricerca Alimenti e Nutrizione 1. EFSA Support. Publ.

[25] Leclercq C., Arcella D., Piccinelli R., Sette S., Le Donne C., Turrini A. (2009). The Italian National Food Consumption Survey INRAN-SCAI 2005-06: main results in terms of food consumption. Public. Health. Nutr..

[26] Tapsell L.C., Neale E.P., Satija A., Hu F.B. (2016). Foods, nutrients, and dietary patterns: interconnections and implications for dietary guidelines. Adv. Nutr..

[27] Goodman S.N., Fanelli D., Ioannidis J.P. (2016). What does research reproducibility mean?. Sci. Transl. Med..

[28] Page M.J., McKenzie J.E., Bossuyt P.M., Boutron I., Hoffmann T.C., Mulrow C.D. (2021). The PRISMA 2020 statement: an updated guideline for reporting systematic reviews. BMJ.

[29] (2014).

[30] Haven S., ten Berge J.M. (1977).

[31] Lorenzo-Seva U., Ten Berge J.M. (2006). Tucker's congruence coefficient as a meaningful index of factor similarity. Methodology.

[32] R Development Core Team (2022). http://www.R-project.org.

[33] Revelle W. Psych: procedures for psychological, psychometric, and personality research. Version 2.3.9. Northwestern University, Evanston, IL. Available from:. https://CRAN.R-project.org/package=psych.

[34] Edefonti V., Decarli A., La Vecchia C., Bosetti C., Randi G., Franceschi S. (2008). Nutrient dietary patterns and the risk of breast and ovarian cancers. Int. J. Cancer..

[35] Bertuccio P., Edefonti V., Bravi F., Ferraroni M., Pelucchi C., Negri E. (2009). Nutrient dietary patterns and gastric cancer risk in Italy. Cancer. Epidemiol. Biomarkers. Prev..

[36] Edefonti V., Bravi F., La Vecchia C., Randi G., Ferraroni M., Garavello W. (2010). Nutrient-based dietary patterns and the risk of oral and pharyngeal cancer. Oral. Oncol..

[37] Bravi F., Edefonti V., Bosetti C., Talamini R., Montella M., Giacosa A. (2010). Nutrient dietary patterns and the risk of colorectal cancer: a case-control study from Italy. Cancer. Causes. Control..

[38] Edefonti V., Bravi F., Garavello W., La Vecchia C., Parpinel M., Franceschi S. (2010). Nutrient-based dietary patterns and laryngeal cancer: evidence from an exploratory factor analysis. Cancer. Epidemiol. Biomarkers. Prev..

[39] Bravi F., Edefonti V., Randi G., Garavello W., La Vecchia C., Ferraroni M. (2012). Dietary patterns and the risk of esophageal cancer. Ann. Oncol..

[40] Bosetti C., Bravi F., Turati F., Edefonti V., Polesel J., Decarli A. (2013). Nutrient-based dietary patterns and pancreatic cancer risk. Ann. Epidemiol..

[41] Rosato V., Edefonti V., Bravi F., Bosetti C., Bertuccio P., Talamini R. (2014). Nutrient-based dietary patterns and prostate cancer risk: a case-control study from Italy. Cancer. Causes. Control.

[42] Bravi F., Bertuccio P., Turati F., Serraino D., Edefonti V., Dal Maso L. (2015). Nutrient-based dietary patterns and endometrial cancer risk: an Italian case-control study. Cancer. Epidemiol..

[43] Edefonti V., Nicolussi F., Polesel J., Bravi F., Bosetti C., Garavello W. (2015). Nutrient-based dietary patterns and nasopharyngeal cancer: evidence from an exploratory factor analysis. Br. J. Cancer..

[44] Dalmartello M., Bravi F., Serraino D., Crispo A., Ferraroni M., La Vecchia C. (2020). Dietary patterns in Italy and the risk of renal cell carcinoma. Nutrients.

[45] Edefonti V., La Vecchia C., Di Maso M., Crispo A., Polesel J., Libra M. (2020). Association between nutrient-based dietary patterns and bladder cancer in Italy. Nutrients.

[46] Edefonti V., Parpinel M., Ferraroni M., Boracchi P., Schioppo T., Scotti I. (2020). A posteriori dietary patterns and rheumatoid arthritis disease activity: a beneficial role of vegetable and animal unsaturated fatty acids. Nutrients.

[47] Marinoni M., Giordani E., Mosconi C., Rosolen V., Concina F., Fiori F. (2022). Are dietary patterns related to cognitive performance in 7-year-old children? Evidence from a birth cohort in Friuli Venezia Giulia. Italy. Nutrients..

[48] Centritto F., Iacoviello L., di Giuseppe R., De Curtis A., Costanzo S., Zito F. (2009). Dietary patterns, cardiovascular risk factors and C-reactive protein in a healthy Italian population. Nutr. Metab. Cardiovasc. Dis..

[49] Bonaccio M., Bonanni A.E., Di Castelnuovo A., De Lucia F., Donati M.B., de Gaetano G. (2012). Low income is associated with poor adherence to a Mediterranean diet and a higher prevalence of obesity: cross-sectional results from the Moli-sani study. BMJ. Open..

[50] Bonaccio M., Di Castelnuovo A., Costanzo S., De Lucia F., Olivieri M., Donati M.B. (2012). Mass media information and adherence to Mediterranean diet: results from the Moli-sani study. Int. J. Public. Health..

[51] Bonaccio M., Di Castelnuovo A., Costanzo S., De Lucia F., Olivieri M., Donati M.B. (2013). Nutrition knowledge is associated with higher adherence to Mediterranean diet and lower prevalence of obesity. Results from the Moli-sani study. Appetite.

[52] Bonanni A.E., Bonaccio M., di Castelnuovo A., de Lucia F., Costanzo S., Persichillo M. (2013). Food labels use is associated with higher adherence to Mediterranean diet: results from the Moli-sani study. Nutrients.

[53] Bonaccio M., Di Castelnuovo A., Bonanni A., Costanzo S., De Lucia F., Pounis G. (2013). Adherence to a Mediterranean diet is associated with a better health-related quality of life: a possible role of high dietary antioxidant content. BMJ. Open..

[54] Bonaccio M., Di Castelnuovo A., Costanzo S., Persichillo M., De Curtis A., Donati M.B. (2016). Adherence to the traditional Mediterranean diet and mortality in subjects with diabetes. Prospective results from the MOLI-SANI study. Eur. J. Prev. Cardiol..

[55] Bonaccio M., Di Castelnuovo A., Costanzo S., Pounis G., Persichillo M., Cerletti C. (2018). Mediterranean-type diet is associated with higher psychological resilience in a general adult population: findings from the Moli-sani study. Eur. J. Clin. Nutr..

[56] Pala V., Sieri S., Masala G., Palli D., Panico S., Vineis P. (2006). Associations between dietary pattern and lifestyle, anthropometry and other health indicators in the elderly participants of the EPIC-Italy cohort. Nutr. Metab. Cardiovasc. Dis..

[57] Masala G., Ceroti M., Pala V., Krogh V., Vineis P., Sacerdote C. (2007). A dietary pattern rich in olive oil and raw vegetables is associated with lower mortality in Italian elderly subjects. Br. J. Nutr..

[58] Jannasch F., Kroger J., Agnoli C., Barricarte A., Boeing H., Cayssials V. (2019). Generalizability of a diabetes-associated country-specific exploratory dietary pattern is feasible across European populations. J. Nutr..

[59] Sieri S., Krogh V., Pala V., Muti P., Micheli A., Evangelista A. (2004). Dietary patterns and risk of breast cancer in the ORDET cohort. Cancer. Epidemiol. Biomarkers. Prev..

[60] Sant M., Allemani C., Sieri S., Krogh V., Menard S., Tagliabue E. (2007). Salad vegetables dietary pattern protects against HER-2-positive breast cancer: a prospective Italian study. Int. J. Cancer..

[61] Menotti A., Alberti-Fidanza A., Fidanza F., Lanti M., Fruttini D. (2012). Factor analysis in the identification of dietary patterns and their predictive role in morbid and fatal events. Public. Health. Nutr..

[62] Menotti A., Puddu P.E. (2018). Comparison of four dietary scores as determinants of coronary heart disease mortality. Sci. Rep..

[63] Maugeri A., Barchitta M., Agrifoglio O., Favara G., La Mastra C., La Rosa M.C. (2019). The impact of social determinants and lifestyles on dietary patterns during pregnancy: evidence from the “Mamma & Bambino” study. Ann. Ig..

[64] Maugeri A., Barchitta M., Favara G., La Rosa M.C., La Mastra C., Magnano San Lio R. (2019). Maternal dietary patterns are associated with pre-pregnancy body mass index and gestational weight gain: results from the “Mamma & Bambino” Cohort. Nutrients.

[65] Magnano San Lio R., Barchitta M., Maugeri A., La Rosa M.C., Giunta G., Panella M. (2022). The impact of the COVID-19 pandemic on dietary patterns of pregnant women: a comparison between two mother-child cohorts in Sicily. Italy. Nutrients..

[66] Ojeda-Granados C., Barchitta M., La Rosa M.C., La Mastra C., Roman S., Panduro A. (2022). Evaluating Dietary Patterns in Women from Southern Italy and Western Mexico. Nutrients.

[67] Barchitta M., Maugeri A., Quattrocchi A., Agrifoglio O., Scalisi A., Agodi A. (2018). The association of dietary patterns with high-risk human papillomavirus infection and cervical cancer: a cross-sectional study in Italy. Nutrients.

[68] Barchitta M., Maugeri A., Magnano San Lio R., Favara G., La Rosa M.C., La Mastra C. (2019). Dietary patterns are associated with leukocyte LINE-1 methylation in women: a cross-sectional study in Southern Italy. Nutrients.

[69] Barchitta M., Maugeri A., Agrifoglio O., Favara G., La Mastra C., La Rosa M.C. (2019). Dietary patterns and school performance: evidence from a sample of adolescents in Sicily. Italy. Ann. Ig..

[70] Fernandez-Alvira J.M., Bammann K., Pala V., Krogh V., Barba G., Eiben G. (2014). Country-specific dietary patterns and associations with socioeconomic status in European children: the IDEFICS study. Eur. J. Clin. Nutr..

[71] Naska A., Fouskakis D., Oikonomou E., Almeida M.D., Berg M.A., Gedrich K. (2006). Dietary patterns and their socio-demographic determinants in 10 European countries: data from the DAFNE databank. Eur. J. Clin. Nutr..

[72] Bravi F., Di Maso M., Eussen S., Agostoni C., Salvatori G., Profeti C. (2021). Dietary patterns of breastfeeding mothers and human milk composition: data from the Italian MEDIDIET study. Nutrients.

[73] Lasalvia P., Gianfagna F., Veronesi G., Franchin M., Tozzi M., Castelli P. (2021). Identification of dietary patterns in a general population of North Italian adults and their association with arterial stiffness. The RoCAV study. Nutr. Metab. Cardiovasc. Dis..

[74] Zupo R., Sardone R., Donghia R., Castellana F., Lampignano L., Bortone I. (2020). Traditional dietary patterns and risk of mortality in a longitudinal cohort of the Salus in Apulia Study. Nutrients.

[75] Tatoli R., Lampignano L., Bortone I., Donghia R., Castellana F., Zupo R. (2022). Dietary patterns associated with diabetes in an older population from Southern Italy using an unsupervised learning approach. Sensors. (Basel)..

[76] Giontella A., Bonafini S., Tagetti A., Bresadola I., Minuz P., Gaudino R. (2019). Relation between dietary habits, physical activity, and anthropometric and vascular parameters in children attending the primary school in the Verona South District. Nutrients.

[77] Turroni S., Petracci E., Edefonti V., Giudetti A.M., D'Amico F., Paganelli L. (2021). Effects of a diet based on foods from symbiotic agriculture on the gut microbiota of subjects at risk for metabolic syndrome. Nutrients.

[78] Donati Zeppa S., Sisti D., Amatori S., Gervasi M., Agostini D., Piccoli G. (2020). High-intensity interval training promotes the shift to a health-supporting dietary pattern in young adults. Nutrients.

[79] Colica C., Mazza E., Ferro Y., Fava A., De Bonis D., Greco M. (2017). Dietary patterns and fractures risk in the elderly. Front. Endocrinol. (Lausanne)..

[80] Mazza E., Fava A., Ferro Y., Moraca M., Rotundo S., Colica C. (2017). Impact of legumes and plant proteins consumption on cognitive performances in the elderly. J. Transl. Med..

[81] Palli D., Russo A., Decarli A. (2001). Dietary patterns, nutrient intake and gastric cancer in a high-risk area of Italy. Cancer. Causes. Control.

[82] Anelli G.M., Parisi F., Sarno L., Fornaciari O., Carlea A., Coco C. (2022). Associations between maternal dietary patterns, biomarkers and delivery outcomes in healthy singleton pregnancies: multicenter Italian GIFt study. Nutrients.

[83] Ruggieri S., Drago G., Panunzi S., Rizzo G., Tavormina E.E., Maltese S. (2022). The influence of sociodemographic factors, lifestyle, and risk perception on dietary patterns in pregnant women living in highly contaminated areas: data from the NEHO birth cohort. Nutrients.

[84] Wingrove K., Lawrence M.A., McNaughton S.A. (2022). A systematic review of the methods used to assess and report dietary patterns. Front. Nutr..

[85] Pett M.A., Lackey N.R., Sullivan J.J. (2003).

[86] Vitale M., Giosue A., Vaccaro O., Riccardi G. (2021). Recent trends in dietary habits of the Italian population: potential impact on health and the environment. Nutrients.

