# Supplementary material for: Are Major a Posteriori Dietary Patterns Reproducible in the Italian Population? A Systematic Review and Quantitative Assessment
Source: Adv Nutr. 2023 Dec 23;15(2):100165. doi: 10.1016/j.advnut.2023.100165 (PMC10818059; doi:10.1016/j.advnut.2023.100165)
Supplement: Multimedia component 1 [file mmc1.pdf]

Manuscript: “Are major a posteriori dietary patterns reproducible in the Italian population? A systematic review and quantitative assessment”

Rachele Bianco
Online Supplemental Material

Supplemental methods

Eligibility criteria

Articles were excluded if: 1. they did not provide original data (e.g., reviews, commentaries, editorials, or personal opinions), or they were case reports, in vitro and in vivo animal studies, conference abstracts or posters; 2. the reference population lived outside Italy, or populations from different countries, including Italy, were available, but it was not possible to separate out the Italian-specific DPs of interest; 3. results concerned single nutrients, single food items, or single food groups; 4. the term DP was used to identify dietary attitudes and perceptions (e.g., feelings felt during meal times, sense of anxiety, perception of self-body image) or patterns of meals; 5. DPs were identified using the *a priori* approach, the mixed-type approach, or the *a posteriori* approach but not following PCA or EFA (e.g., cluster analysis, latent class models, or treelet transform); 6. PCA or EFA were applied on dietary behaviors and not on dietary components; and 7. PCA or EFA were applied on lifestyle variables, including diet, to derive lifestyle risk patterns.
No restrictions were imposed on year of publication, population characteristics, or health status.

Search strategy

Each search string included the following terms: “Feeding Behavior” OR “Diet, Western” OR “diet quality” OR “dietary pattern” OR “diet pattern” OR “food pattern” OR “food intake pattern” OR “food consumption pattern” OR “eating pattern” AND “Factor Analysis, Statistical” OR “Principal Component Analysis” OR “factor” OR “component” OR “score” OR “cluster” AND “Italy” or “Italian”, as both keywords and MeSH/Emtree terms. Details on the single strings used were provided below.

PubMed	("Feeding Behavior"[Mesh] OR "Diet, Western"[Mesh] OR "diet qualit*" OR "dietary pattern*" OR "diet pattern*" OR "food pattern*" OR "food intake pattern*" OR "food consumption pattern" OR "eating pattern*") AND ("Factor Analysis, Statistical"[Mesh] OR "Principal Component Analysis"[Mesh] OR factor* OR component* OR score* OR cluster*) AND (Italy OR Italian)
Embase	('dietary quality'/exp OR 'dietary pattern'/exp OR 'dietary pattern*' OR 'diet pattern*' OR 'food pattern*' OR 'food intake pattern*' OR 'food consumption pattern*' OR 'eating pattern*' OR 'diet qualit*') AND ('factor analysis'/exp OR 'component analysis'/exp OR 'factor analysis*' OR 'component analysis*' OR factor* OR component* OR cluster* OR score*) AND ('Italy'/exp OR 'italian'/exp OR Italy OR italian)

Cochrane	("Feeding Behavior" OR "Diet, Western" OR “dietary pattern*” OR “diet pattern*” OR “food pattern*” OR “food intake pattern*” OR “food consumption pattern” OR “eating pattern*” OR "diet qualit*") AND ("Factor Analysis, Statistical" OR "Principal Component Analysis" OR factor* OR component* OR score* OR cluster*) AND (Italy OR italian)

Data extraction

Using a predefined Excel spreadsheet, data extraction was performed independently by two investigators (RB and MT). Data extraction was checked by other two investigators (VE and MS) and a third one (MF) was involved in resolving any potential disagreement. Information extracted from each study included the following: 1. general characteristics of the studies (first author, year of publication, country, and study name); 2. study design and characteristics (type of design, brief description of data collection, study location, age, sex, and sample size); 3. dietary assessment tool used (type, reference period, reproducibility and validity, and form of administration); 4. DP identification method (number of foods/nutrients considered, pre-processing of foods/nutrients, estimation method, rotation, criteria for choosing the number of factors to retain, factor labelling strategy, checks of factorability, internal consistency, and internal reproducibility); 5. Number of DPs, proportion of variance explained, name and composition; 6. statistical methods used for relating the identified DPs to disease outcomes/determinants/correlates, and 7. main results on the relationship between identified DPs and disease outcomes/determinants/correlates (corresponding to those statistical models adjusted for all the available confounders, if models were fitted).

Supplemental Table 1. Quality assessment of the included studies according to study design[1]

	Case-control studies (12 items)													
	1	2	3	4	5	6	7	8	9	10	11	12	-	-
	Was the research question or objective in this paper clearly stated and appropriate?	Was the study population clearly specified and defined?	Did the authors include a sample size justification?	Were controls selected or recruited from the same or similar population that gave rise to the cases (including the same timeframe)?	Were the definitions, inclusion and exclusion criteria, algorithms or processes used to identify or select cases and controls valid, reliable, and implemented consistently across all study participants?	Were the cases clearly defined and differentiated from controls?	If less than 100 percent of eligible cases and/or controls were selected for the study, were the cases and/or controls randomly selected from those eligible?	Was there use of concurrent controls?	Were the investigators able to confirm that the exposure/risk occurred prior to the development of the condition or event that defined a participant as a case?	Were the measures of exposure/risk clearly defined, valid, reliable, and implemented consistently (including the same time period) across all study participants?	Were the assessors of exposure/risk blinded to the case or control status of participants?	Were key potential confounding variables measured and adjusted statistically in the analyses? If matching was used, did the investigators account for matching during study analysis?	-	-
Edefonti, 2008 (34)	Y	Y	N	Y	Y	Y	N	Y	Y	Y	N	Y	-	-
Bertuccio, 2009 (35)	Y	Y	N	Y	Y	Y	N	Y	Y	Y	N	Y	-	-
Edefonti, 2010 (36)	Y	Y	N	Y	Y	Y	N	Y	Y	Y	N	Y	-	-
Bravi, 2010 (37)	Y	Y	N	Y	Y	Y	N	Y	Y	Y	N	Y	-	-
Edefonti, 2010 (38)	Y	Y	N	Y	Y	Y	N	Y	Y	Y	N	Y	-	-
Bravi, 2012 (39)	Y	Y	N	Y	Y	Y	N	Y	Y	Y	N	Y	-	-
Bosetti, 2013 (40)	Y	Y	N	Y	Y	Y	N	Y	Y	Y	N	Y	-	-

Rosato, 2014 (41)	Y	Y	N	Y	Y	Y	N	Y	Y	Y	N	Y	-	-
Bravi, 2015 (42)	Y	Y	N	Y	Y	Y	N	Y	Y	Y	N	Y	-	-
Edefonti, 2015 (43)	Y	Y	N	Y	Y	Y	N	Y	Y	Y	N	N	-	-
Dalmartello, 2020 (44)	Y	Y	N	Y	Y	Y	N	Y	Y	Y	N	Y	-	-
Edefonti, 2020 (45)	Y	Y	N	Y	Y	Y	N	Y	Y	Y	N	Y	-	-
Palli, 2001 (81)	Y	Y	N	Y	Y	Y	NR	N	Y	Y	N	Y	-	-
	**Cohort and cross-sectional studies (14 items)**													
	1	2	3	4	5	6	7	8	9	10	11	12	13	14
	Was the research question or objective in this paper clearly stated?	Was the study population clearly specified and defined?	Was the participation rate of eligible persons at least 50%?	Were all the subjects selected or recruited from the same or similar populations (including the same time period)? Were inclusion and exclusion criteria for being in the study prespecified and applied uniformly to all participants?	Was a sample size justification, power description, or variance and effect estimates provided?	For the analyses in this paper, were the exposure(s) of interest measured prior to the outcome(s) being measured?	Was the timeframe sufficient so that one could reasonably expect to see an association between exposure and outcome if it existed?	For exposures that can vary in amount or level, did the study examine different levels of the exposure as related to the outcome (e.g., categories of exposure, or exposure measured as continuous variable)?	Were the exposure measures (independent variables) clearly defined, valid, reliable, and implemented consistently across all study participants?	Was the exposure(s) assessed more than once over time?	Were the outcome measures (dependent variables) clearly defined, valid, reliable, and implemented consistently across all study participants?	Were the outcome assessors blinded to the exposure status of participants?	Was loss to follow-up after baseline 20% or less?	Were key potential confounding variables measured and adjusted statistically for their impact on the relationship between exposure(s) and outcome(s)?
Edefonti, 2020 (46)	Y	Y	Y	Y	N	N	N	Y	Y	NA	Y	NR	NA	Y

Marinoni, 2022 (47)	Y	Y	Y	Y	N	N	N	Y	Y	NA	Y	Y	NA	Y
Centritto, 2009 (48)	Y	Y	Y	Y	N	N	N	Y	Y	NA	Y	Y	NA	Y
Bonaccio, 2012 (49)	Y	Y	Y	Y	N	N	N	Y	Y	NA	Y	Y	NA	Y
Bonaccio, 2012 (50)	Y	Y	Y	Y	N	N	N	Y	Y	NA	Y	Y	NA	Y
Bonaccio, 2013 (51)	Y	Y	Y	Y	N	N	N	Y	Y	NA	Y	Y	NA	Y
Bonanni, 2013 (52)	Y	Y	Y	Y	N	N	N	N	Y	NA	Y	Y	NA	Y
Bonaccio, 2013 (53)	Y	Y	Y	Y	N	N	N	Y	Y	NA	Y	Y	NA	Y
Bonaccio, 2016 (54)	Y	Y	NR	Y	N	Y	N	Y	Y	N	Y	Y	Y	Y
Bonaccio, 2018 (55)	Y	Y	Y	Y	Y	N	N	Y	Y	NA	Y	Y	NA	Y
Pala, 2006 (56)	Y	Y	Y	Y	N	N	N	Y	Y	NA	Y	Y	NA	N
Masala, 2007 (57)	Y	Y	Y	Y	N	Y	Y	Y	Y	N	Y	Y	Y	Y
Jannasch, 2019 (58)	Y	Y	Y	N	N	Y	Y	Y	Y	N	Y	Y	NR	Y
Balder, 2003 (11)	Y	Y	NR	N	N	NA	NA	NA	Y	NA	NA	NA	NA	NA
Männistö, 2005 (12)	Y	Y	NR	N	Y	Y	Y	Y	Y	N	Y	Y	NR	Y
Sieri, 2004 (59)	Y	Y	NR	Y	N	Y	Y	Y	Y	N	Y	Y	Y	Y
Sant Sant, 2007 (60)	Y	Y	NR	Y	N	Y	Y	Y	Y	N	Y	Y	Y	Y
Menotti, 2012 (61)	Y	Y	Y	Y	N	Y	Y	Y	Y	N	Y	Y	NR	Y
Menotti, 2018 (62)	N	Y	Y	Y	N	Y	Y	Y	Y	N	Y	Y	Y	Y
Maugeri, 2019 (63)	Y	Y	NR	Y	N	N	N	Y	Y	NA	Y	N	NA	Y
Maugeri, 2019 (64)	Y	Y	NR	Y	N	N	N	Y	Y	NA	Y	N	NA	Y
Magnano San Lio, 2022 (65)	Y	Y	NR	Y	N	N	N	Y	Y	NA	Y	Y	NA	Y
Ojeda-Granados, 2022 (66)	Y	Y	NR	Y	N	N	N	Y	Y	NA	Y	NR	NA	N

Barchitta, 2018 (67)	Y	Y	NR	Y	N	N	N	Y	Y	NA	Y	Y	NA	Y
Barchitta, 2019 (68)	Y	Y	NR	Y	N	N	N	Y	Y	NA	Y	Y	NA	Y
Barchitta, 2019 (69)	Y	N	NR	Y	N	N	N	Y	Y	NA	Y	Y	NA	N
Fernández-Alvira, 2014 (70)	Y	Y	Y	Y	N	N	N	Y	Y	NA	Y	Y	NA	Y
Naska, 2006 (71)	Y	Y	NA	N	N	N	N	Y	N	NA	Y	Y	NA	N
Bravi, 2021 (72)	Y	Y	NR	Y	N	N	N	Y	Y	NA	Y	Y	NA	N
Lasalvia, 2021 (73)	Y	Y	Y	Y	N	N	N	Y	Y	NA	Y	Y	NA	Y
Zupo, 2020 (74)	Y	N	Y	Y	N	Y	Y	Y	Y	N	Y	Y	Y	Y
Tatoli, 2022 (75)	Y	Y	NR	Y	N	NA	NA	NA	Y	NA	NA	NA	NA	NA
Giontella, 2019 (76)	Y	Y	Y	N	Y	N	N	Y	Y	NA	Y	N	NA	Y
Colica, 2017 (79)	Y	Y	NR	Y	Y	N	N	Y	Y	NA	N	NR	NA	Y
Mazza, 2017 (80)	Y	Y	NR	Y	Y	Y	Y	Y	Y	N	N	Y	N	Y
Anelli, 2022 (82)	Y	Y	NR	Y	N	Y	Y	Y	Y	N	Y	Y	Y	Y
Ruggieri, 2022 (83)	Y	Y	Y	Y	N	N	N	Y	Y	NA	Y	Y	NA	Y
	**Trials (14 items)**													
	1	2	3	4	5	6	7	8	9	10	11	12	13	14

	Was the study described as randomized, a randomized trial, a randomized clinical trial, or an RCT?	Was the method of randomization adequate (i.e., use of randomly generated assignment)?	Was the treatment allocation concealed (so that assignments could not be predicted)?	Were study participants and providers blinded to treatment group assignment?	Were the people assessing the outcomes blinded to the participants' group assignments?	Were the groups similar at baseline on important characteristics that could affect outcomes (e.g., demographics, risk factors, co-morbid conditions)?	Was the overall drop-out rate from the study at endpoint 20% or lower of the number allocated to treatment?	Was the differential drop-out rate (between treatment groups) at endpoint 15 percentage points or lower?	Was there high adherence to the intervention protocols for each treatment group?	Were other interventions avoided or similar in the groups (e.g., similar background treatments)?	Were outcomes assessed using valid and reliable measures, implemented consistently across all study participants?	Did the authors report that the sample size was sufficiently large to be able to detect a difference in the main outcome between groups with at least 80% power?	Were outcomes reported or subgroups analyzed prespecified (i.e., identified before analyses were conducted)?	Were all randomized participants analyzed in the group to which they were originally assigned, i.e., did they use an intention-to-treat analysis?
Turroni, 2021 (77)	N	Y	N	N	Y	N	Y	Y	NR	Y	Y	N	Y	Y
Donati Zeppa, 2020 (78)	N	N	N	N	NR	NA	Y	Y	Y	Y	Y	N	Y	NA

[1]For each quality assessment tool, each row reported the distribution of replies (“Yes”, “No”, “Not applicable”, and “Not reported”) to single questions. “Cannot determine” reply was never used during this quality assessment evaluation.
ABBREVIATIONS: NA, Not Applicable; NR, Not reported

Supplemental Table 2. Main characteristics of studies identifying dietary patterns using principal component and factor analyses in Italy[1]

Reference, location, study name, study quality	Study design	Participants	Dietary questionnaire
Edefonti, 2008 (34)<br>Breast cancer: northern Italy (Milan, Genoa, Gorizia, Forli), central and southern Italy (Latina, Naples)<br>Ovarian cancer: northern Italy (Milan, Pordenone, Padua), central and southern Italy (Latina, Naples)<br>Good quality	Case-control study; 2 companion studies on breast and ovarian cancers; hospital based; recruitment from 1991 to 1994 for the breast cancer study and from 1992 to 1999 for the ovarian cancer study; Italian multicentric	7013 total subjects (100% Fs); 2569 breast cancer cases 25-74 ys (median: 55 ys, NA); 1031 ovarian cancers cases 18-79 ys (median: 56 ys, NA); 3413 controls 17-79 ys (median: 57 ys, NA)	FFQ<br>2 ys before<br>IA<br>Reproducible and valid<br>78 FIs (30 NUTs)
Bertuccio, 2009 (35)<br>Milan (Lombardy)<br>Good quality	Case-control study; gastric cancer; hospital based; recruitment from 1997 to 2007; single center/area	777 total subjects; 230 cases (143 Ms, 87 Fs)<br>22-80 ys (median: 63 ys, NA); 547 controls (286 Ms, 261 Fs) 22-80 ys (median: 63 ys, NA)	FFQ<br>2 ys before<br>IA<br>reproducible and valid<br>78 FIs (28 NUTs)
Edefonti, 2010 (36)<br>Milan (Lombardy), Pordenone (Friuli Venezia Giulia), Rome, Latina (Lazio)<br>Good quality	Case-control study; oral cavity cancer; hospital based; recruitment from 1992 to 2005; Italian multicentric	2886 total subjects; 805 cases (659 Ms, 146 Fs) 22-78 ys (median: 58 ys, NA); 2081 controls (1302 Ms, 779 Fs); 19-79 ys (median: 58 ys, NA)	FFQ<br>2 ys before<br>IA<br>Reproducible and valid<br>78 FIs (29 NUTs)
Bravi, 2010 (37)<br>Milan (Lombardy), Genoa (Liguria), Pordenone, Gorizia (Friuli Venezia Giulia), Forlì (Emilia-Romagna), Latina (Lazio), Naples (Campania)<br>Good quality	Case-control study; colorectal cancer; hospital based; recruitment from 1992 to 1996; Italian multicentric	6107 total subjects; 1225 colon cancer cases (688 Ms, 537 Fs) 19-74 ys (median: 62 ys, NA); 728 rectum cancer cases (437 Ms, 291 Fs) 23-74 ys (median: 62 ys, NA); 4154 controls (2073 Ms, 2081 Fs) 19-74 ys (median: 58 ys, NA)	FFQ<br>2 ys before<br>IA<br>Reproducible and valid<br>78 FIs (28 NUTs)

Edefonti, 2010 (38)<br>Milan (Lombardy), Pordenone (Friuli Venezia Giulia)<br>Good quality	Case-control study; laryngeal cancer; hospital based; recruitment from 1992 to 2000; Italian multicentric	1548 total subjects; 460 cases (415 Ms, 45 Fs) 30-80 ys (median: 61 ys, NA); 1088 controls (863 Ms, 225 Fs) 31-79 ys (median: 61 ys, NA)	FFQ<br>2 ys before<br>IA<br>Reproducible and valid<br>78 FIs (28 NUTs)
Bravi, 2012 (39)<br>Milan (Lombardy), Pordenone (Friuli Venezia Giulia), Padua (Veneto)<br>Good quality	Case-control study; esophageal cancer; hospital based; recruitment from 1992 to 1997; Italian multicentric	1047 total subjects; 304 cases (275 Ms, 29 Fs) 39-77 ys (median: 60 ys, NA); 743 controls (593 Ms, 150 Fs) 33-77 ys (median: 60 ys, NA)	FFQ<br>2 ys before<br>IA<br>Reproducible and valid<br>78 FIs (28 NUTs)
Bosetti, 2013 (40)<br>Milan (Lombardy), Pordenone (Friuli Venezia Giulia)<br>Good quality	Case-control study; pancreatic cancer; hospital based; recruitment from 1991 to 2008; Italian multicentric	978 total subjects; 326 cases (174 Ms, 152 Fs) (median: 63 ys, NA); 652 controls (348 Ms, 304 Fs) (median: 62 ys, NA)	FFQ<br>2 ys before<br>IA<br>Reproducible and valid<br>78 FIs (28 NUTs)
Rosato, 2014 (41)<br>Milan (Lombardy), Pordenone, Gorizia (Friuli Venezia Giulia)<br>Latina (Lazio), Naples (Campania)<br>Good quality	Case-control study; prostate cancer; hospital based; recruitment from 1991 to 2002; Italian multicentric	2745 total subjects (100% Ms); 1294 cases 46-74 ys (median: 66 ys, NA); 1451 controls 46-74 ys (median: 63 ys, NA)	FFQ<br>2 ys before<br>IA<br>Reproducible and valid<br>78 FIs (28 NUTs)
Bravi, 2015 (42)<br>Milan (Lombardy), Pordenone, Udine (Friuli Venezia Giulia), Naples (Campania)<br>Good quality	Case-control study; endometrial cancer; hospital based; recruitment from 1992 to 2006; Italian multicentric	1362 total subjects (100% Fs); 454 cases 18-79 ys (median: 60 ys, NA); 908 controls 19-80 ys (median: 61 ys, NA)	FFQ<br>2 ys before<br>IA<br>Reproducible and valid<br>78 FIs (28 NUTs)
Edefonti, 2015 (43)<br>Milan (Lombardy), Pordenone (Friuli Venezia Giulia), Naples (Campania), Catania (Sicily)<br>Good quality	Case-control study; nasopharyngeal cancer; hospital based; recruitment from 1992 to 2008; Italian multicentric	792 total subjects; 198 cases (157 Ms, 41 Fs) 18-76 ys (median: 52 ys, NA); 594 controls (471 Ms, 123 Fs) 19-76 ys (median: 52 ys, NA)	FFQ<br>2 ys before<br>IA<br>Reproducible and valid<br>78 FIs (28 NUTs)

Dalmartello, 2020 (44)<br>Milan (Lombardy), Pordenone, Udine (Friuli Venezia Giulia), Latina (Lazio), Naples (Campania)<br>Good quality	Case-control study; renal cell cancer; hospital based; recruitment from 1992 to 2004; Italian multicentric	2301 total subjects; 767 cases (494 Ms, 273 Fs) 24-79 ys (median: 62 ys, NA); 1534 controls (988 Ms, 546 Fs) 22-79 ys (median: 62 ys, NA)	FFQ<br>2 ys before<br>IA<br>Reproducible and valid<br>78 FIs (28 NUTs)
Edefonti, 2020 (45)<br>Milan (Lombardy), Pordenone (Friuli Venezia Giulia), Naples (Campania), Catania (Sicily)<br>Good quality	Case-control study; bladder cancer; hospital based; recruitment from 2003 to 2014; Italian multicentric	1355 total subjects; 690 cases (595 Ms, 95 Fs) 25-84 ys (median: 67 ys, NA); 665 controls (561 Ms, 104 Fs) 27-84 ys (median: 66 ys, NA)	FFQ<br>2 ys before<br>IA<br>Reproducible and valid<br>80 FIs (28 NUTs)
Edefonti, 2020 (46)<br>Milan (Lombardy)<br>Good quality	Cross-sectional study; rheumatoid arthritis disease activity; recruitment from January 2018 to December 2019; single center/area with recruitment at Pini Hospital (Milan)	205 total subjects (40 Ms, 165 Fs) 18-65 ys (median: 58.46 ys, IQR: 47.81-69.03 ys)	FFQ<br>6 mos before<br>IA<br>Reproducible and valid<br>110 FIs (33 NUTs)
Marinoni, 2022 (47)<br>Croatia, Greece, Italy (Friuli Venezia Giulia region)<br>Good quality	Cross-sectional analysis nested within the NAC-II birth cohort which followed-up 632 eligible (i.e, 18-month children with neurodevelopment assessed) born from 767 mothers originally recruited between 2007 and 2009; international	379 total subjects (195 Ms, 184 Fs); mean: 7 ys, SD: 0.05 ys	3d-DR (2 weekdays and 1 weekend day, not necessarily consecutive) in the wk before<br>IA<br>Reproducible and valid<br>828 FIs (37 NUTs)
Centritto, 2009 (48)<br>Molise<br>Moli-sani<br>Good quality	Cross-sectional study; men and women living in Molise randomly recruited from city-hall registries of Molise by using electronically generated numbers; 16704 subjects recruited from 2005 to 2008; single center/area	7646 total subjects (49% Ms, 51% Fs); age ≥ 35 ys (mean: 50 ys, SE: 10 ys)	Modified version of the reproducible and valid EPIC FFQ to include some typical southern Italy foods<br>NA reference period<br>SA<br>Validated in a different form<br>188 FIs (45 FGs)

Bonaccio, 2012 (49)<br>Molise<br>Moli-sani<br>Good quality	Cross-sectional study; men and women living in Molise randomly recruited from city-hall registries of Molise by using electronically generated numbers; 24325 subjects recruited from March 2005 to April 2010; single center/area	13262 total subjects (6590 Ms, 6672 Fs); age ≥ 35 ys (mean: 53.3 ys, SD: 10.6 ys)	Modified version of the reproducible and valid EPIC FFQ to include some typical southern Italy foods<br>NA reference period<br>SA<br>Validated in a different form<br>188 FIs (43 FGs)
Bonaccio, 2012 (50)<br>Molise<br>Moli-sani<br>Good quality	Cross-sectional study; men and women living in Molise randomly recruited from city-hall registries of Molise by using electronically generated numbers; 1132 subjects recruited from May 2009 to April 2010; single center/area	959 total subjects (479 Ms, 480 Fs) aged ≥ 35 ys (mean: 52.8 ys, SD: 9.6 ys)	Modified version of the reproducible and valid EPIC FFQ to include some typical southern Italy foods<br>NA reference period<br>SA<br>Validated in a different form<br>188 FIs (45 FGs based on reference to a previous paper)
Bonaccio, 2013 (51)<br>Molise<br>Moli-sani<br>Good quality	Cross-sectional study; men and women living in Molise randomly recruited from city-hall registries of Molise by using electronically generated numbers; 1132 subjects recruited from May 2009 to April 2010; single center/area	744 total subjects (50.3% Ms, 49.7% Fs); age ≥ 35 ys (mean: 52.1 ys, SD: 9.4 ys)	Modified version of the reproducible and valid EPIC FFQ to include some typical southern Italy foods<br>NA reference period<br>SA<br>Validated in a different form<br>188 FIs (43 FGs)
Bonanni, 2013 (52)<br>Molise<br>Moli-sani<br>Good quality	Cross-sectional study; men and women living in Molise randomly recruited from city-hall registries of Molise by using electronically generated numbers; 1571 subjects recruited from May 2009 to April 2010; single center/area	883 total subjects (442 Ms, 441 Fs); age ≥ 35 ys (mean: 52.5 ys, SD: 9.6 ys)	Modified version of the reproducible and valid EPIC FFQ to include some typical southern Italy foods<br>NA reference period<br>SA<br>Validated in a different form<br>188 FIs (45 FGs based on reference to a previous paper)

Bonaccio, 2013 (53)<br>Molise<br>Moli-sani<br>Good quality	Cross-sectional study; men and women living in Molise randomly recruited from city-hall registries of Molise by using electronically generated numbers; 24325 subjects recruited from March 2005 to April 2010; single center/area	16937 total subjects (48.4% Ms, 51.6% Fs); age ≥ 35 ys (mean: 53.0 ys, SD: 10.8 ys)	Modified version of the reproducible and valid EPIC FFQ to include some typical southern Italy foods<br>NA reference period<br>SA<br>Validated in a different form<br>188 FIs (43 FGs)
Bonaccio, 2016 (54)<br>Molise<br>Moli-sani<br>Good quality	Prospective cohort study; men and women living in Molise randomly recruited from city-hall registries of Molise by using electronically generated numbers; 24325 subjects recruited from March 2005 to April 2010 for a final sample of 1995 patients with type 2 diabetes followed-up for mortality until December 2011; single center/area	1995 total subjects (1319 Ms, 676 Fs); age ≥ 35 ys (mean: 62.6 ys, SD: 10.2 ys)	Modified version of the reproducible and valid EPIC FFQ to include some typical southern Italy foods<br>NA reference period<br>SA<br>Validated in a different form<br>188 FIs (46 FGs)
Bonaccio, 2018 (55)<br>Molise<br>Moli-sani<br>Good quality	Cross-sectional study; men and women living in Molise randomly recruited from city-hall registries of Molise by using electronically generated numbers; 24325 subjects recruited from March 2005 to April 2010; single center/area	11272 total subjects (46.2% Ms, 53.8% Fs) age ≥ 35 ys (mean: 52.7 ys, SD: 10.8 ys) reduced to 10812 due to unreliable medical or dietary questionnaires, implausible EIs or missing values for dietary information	Modified version of the reproducible and valid EPIC FFQ to include some typical southern Italy foods<br>NA reference period<br>SA<br>Validated in a different form<br>188 FIs (46 FGs)
Pala, 2006 (56)<br>Denmark, France, Germany, Greece, Netherlands, Spain, Sweden, UK, Italy (Varese, Turin, Florence, Naples, Ragusa)<br>EPIC (EPIC-Elderly)<br>Good quality	Cross-sectional analysis nested within a prospective cohort study; elderly (≥60 ys) participants from EPIC study recruited voluntarily from 1993 to 1998 in 5 different areas covered by cancer registries in northern, central and southern Italy; international	100 059 total subjects; 5611 Italian participants: 1536 Ms (60.0-72.2 ys, median age at enrollment: 62.3 ys, IQR: NA), 4075 Fs (60.0-77.8 ys, median age at enrollment: 62.3 ys, IQR: NA)	3 different FFQs<br>1 y before<br>NA<br>Reproducible and valid<br>188 FIs (Varese, Turin, Florence), 217 FIs (Ragusa), 140 FIs (Naples), (57 FGs for all centers)

Masala, 2007 (57)<br>Denmark, France, Germany, Greece, Netherlands, Spain, Sweden, UK, Italy (Varese, Turin, Florence, Naples, Ragusa)<br>EPIC (EPIC-Elderly)<br>Very good quality	Prospective cohort study; elderly (≥60 ys) participants from EPIC study recruited voluntarily in 5 different areas covered by cancer registries in northern, central and southern Italy between 1993 and 1998 and followed-up for overall mortality up to 2001 or 2002 (median follow-up of 6.2 ys after applying exclusion criteria); international	100 059 total subjects; 5611 Italian participants: 1536 Ms (60.0-72.2 ys, median age at enrollment: 62.3 ys, IQR: NA), 4075 Fs (60.0-77.8 ys, median age at enrollment: 62.3 ys, IQR: NA)	3 different FFQs<br>1 y before<br>NA<br>Reproducible and valid<br>188 FIs (Varese, Turin, Florence), 217 FIs (Ragusa), 140 FIs (Naples), (57 FGs for all centers)
Jannasch, 2019 (58)<br>Italy, France, Spain, UK, Netherlands, Germany, Sweden, Denmark<br>EPIC-InterAct<br>Good quality	Case-cohort study nested within EPIC prospective cohort study and based on incident cases of type 2 diabetes in the full EPIC cohort (cases which occurred between 1991 and the 31 December 2007 in 8 countries) and a randomly drawn subcohort stratified by center (9 centers); international	25877 total subjects of which 14694 randomly drawn subcohort subjects and 11183 verified incident type 2 diabetes cases; 719 verified incident type 2 diabetes cases overlapping with the subcohort; 1927 Italian participants in the subcohort (32.3% Ms, 67.7% Fs), mean: 50.2 ys, SD: 7.9 ys at baseline	Reproducible and valid country specific FFQs<br>1 y before<br>NA FIs (36 FGs)
Balder, 2003 (11)<br>Netherlands, Sweden, Finland, and Italy<br>DIETSCAN Project (NLSC, SMC, ATBC, ORDET)<br>Poor quality	Parallel analysis of 4 prospective cohort studies on diet and cancer according to the same strategy (no pooled analysis); NLSC (random subcohort of): population-based cohort of Ms and Fs from Dutch municipalities that began in 1986; SMC: population-based cohort of Fs based on a mammography screening in 2 countries in central Sweden from 1987 to 1990; ATBC: randomized placebo-controlled intervention study conducted among M smokers who lived in south-western Finland (1985–1988); ORDET: cohort study of Italian healthy volunteer Fs from the province of Varese, northern Italy (1987–1992); international	100911 total subjects; ORDET (from Italy): 9208 Fs with complete dietary data (mean age at baseline: 48.6 ys, SE: 8.6 ys, 35–69 ys); median follow-up and number of deaths not reported	4 different but validated FFQs;<br>ORDET-FFQ: 1 y before;<br>SA;<br>Reproducible and valid;<br>107 FIs (51 FGs, but final number equal to 32, due to ORDET availability)

Männistö, 2005 (12)<br>Netherlands, Sweden, and Italy<br>DIETSCAN Project (NLSC, SMC, ATBC, ORDET)<br>Good quality	Parallel analysis of 3 prospective cohort studies on diet and cancer according to the same strategy (no pooled analysis); NLSC (random subcohort of): population-based cohort of Ms and Fs from Dutch municipalities that began in 1986; SMC: population-based cohort of Fs based on a mammography screening in 2 countries in central Sweden from 1987 to 1990; all invasive breast cancer cases were identified through national or local cancer registers; ORDET: cohort study of Italian healthy volunteer Fs from the province of Varese, northern Italy (enrollment from 1987 to 1992; 9 ys follow-up); international; re-analysis of DPs originally derived in Balder et al. 2003	73849 total subjects (3271 breast cancer cases with complete information on their diet); ORDET (from Italy): 10788 Fs (mean age at baseline: 48 ys; SE: 8.5 ys, 35-69 ys), 212 breast cancer cases	3 different but validated FFQs: ORDET-FFQ: 1 y before; SA; Reproducible and valid; 107 FIs (51 FGs, but final number equal to 32)
Sieri, 2004 (59)<br>Varese (Lombardy)<br>ORDET<br>Very good quality	Prospective cohort study; Italian healthy volunteer women from the province of Varese, northern Italy; cancer cases identified through local cancer registry; recruitment from 1987 to 1992; 9.5 ys of average follow-up; single center/area	8984 subjects 100% Fs (34-70 ys) based on a total of 10786 subjects; 207 incident breast cancer cases	FFQ<br>1 y before<br>SA<br>Reproducible and valid<br>107 FIs (34 FGs)
Sant, 2007 (60)<br>Varese (Lombardy)<br>ORDET<br>Very good quality	Prospective cohort study; Italian healthy volunteer women from the province of Varese, northern Italy; cancer cases identified through local cancer registry; recruitment from 1987 to 1992; 11.5 ys of average follow-up; single center/area; re-analysis of DPs originally provided in Sieri et al. 2004	8861 subjects 100% Fs (34-70 ys) based on a total of 8984 subjects recruited in a previous ORDET study; 267 incident breast cancer cases by December 31, 2001, with availability of HER2 status in 238 of them	FFQ<br>1 y before<br>SA<br>Reproducible and valid<br>107 FIs (34 FGs)
Menotti, 2012 (61)<br>Italian Rural Areas of Seven Countries Study of Cardiovascular Disease<br>Seven Countries Study<br>Very good quality	Prospective cohort study; enrollment in 1960 from the Italian Rural Areas cohorts, follow-up of 20 ys for CHD events and 40 ys for mortality; international	1221 total subjects (100% Ms) 45-64 ys at the 5-y follow-up in 1965 (mean: 54.9 ys, SD: 5.0 ys); at 20-y follow-up CHD events were 185 (fatal and non-fatal); at 40-y follow-up deaths were 187 for CHD, 513 for CVD, 324 for cancer, and 1148 for all-cause mortality	Dietary history; Italian Rural Areas administered at the 5-y follow-up in 1965;<br>IA<br>Validated<br>NA FIs (17 FGs)

Menotti, 2018 (62)<br>Italian Rural Areas of Seven Countries Study of Cardiovascular Disease<br>Seven Countries Study<br>Very good quality	Prospective cohort study; enrollment in 1960 from the Italian Rural Areas cohorts, follow-up of 40 ys for mortality; comparison of the role of 4 dietary scores in a sample of middle-aged men followed up during 40 ys for CHD mortality; international	1284 total subjects with final sample size equal to 1214 after excluding 70 subjects with major prevalent CHD (100% Ms); 45-64 ys at the 5-y follow-up in 1965; at 40-y follow-up deaths were 200 from CHD	Dietary history<br>IA<br>Validated<br>NA FIs (17 FGs)
Maugeri, 2019 (63)<br>Mamma & Bambino<br>Catania (Sicily)<br>Fair quality	Cross-sectional study nested within the "Mamma & Bambino" birth cohort of pregnant women referring to “Policlinico Vittorio Emanuele” (Catania, Italy) for the prenatal genetic counselling without pre-existing medical conditions and/or pregnancy complications; recruitment from November 2014 to 2019 (ongoing at publication); single center/area	332 total subjects (100% Fs); 15-50 ys (median: 37 ys, NA); gestational age at recruitment 4-20 gwks (median: 16 gwks, NA)	FFQ<br>1 mo before<br>IA<br>Adapted from a previously validated FFQ<br>95 FIs (39 FGs)
Maugeri, 2019 (64)<br>Mamma & Bambino<br>Catania (Sicily)<br>Fair quality	Cross-sectional study nested within the "Mamma & Bambino" birth cohort enrolling pregnant women referring to “Policlinico-Vittorio Emanuele” (Catania) at 4–20 gwks (median: 16 gwks) with additional exclusion criteria related to the current paper; single center/area	232 total subjects (100% Fs); 15-50 ys (median: 37 ys, NA)	FFQ<br>1 mo before<br>IA<br>Adapted from a previously validated FFQ<br>95 FIs (39 FGs)
Magnano San Lio, 2022 (65)<br>Catania (Sicily)<br>Good quality	Cross-sectional analysis of data from two prospective cohorts; pregnant women enrolled before COVID-19 pandemic (“Mamma & Bambino” cohort, from November 2014 to December 2019, during the prenatal genetic counseling) and during COVID-19 pandemic ("MAMI-MED", from December 2020 to January 2022, during the first trimester visit) in two hospitals in Catania with the aim to evaluate how their dietary habits affect the health of mother-child pairs; Italian multicentric	1097 total subjects (100% Fs); 397 "Mamma & Bambino" (median: 37.0 ys, IQR: 4.0 ys); 801 "MAMI-MED" (median: 31.0 ys, IQR: 7.0 ys)	FFQ for both studies<br>1 mo before<br>IA<br>Adapted from a previously validated FFQ<br>95 FIs (39 FGs)

Ojeda-Granados, 2022 (66)<br>Catania (Sicily), Guadalajara (Mexico)<br>Fair quality	Cross-sectional study; age-matched Italian non-pregnant women with no history of severe diseases recruited among those referring to three clinical laboratories in Catania (Italy) from 2010 to 2017 and from the general adult population referring to University of Guadalajara (Mexico) from 2011 to 2015; international	1026 total subjects (100% Fs), age 18-72 ys; 811 Italian subjects (median: 40 ys, IQR: 19 ys); 215 Mexican subjects (median: 40 ys, IQR: 21 ys)	Italian FFQ: 1 mo before, IA, Adapted from a previously validated FFQ, 95 FIs (39 FGs);<br>Mexican FFQ: NA reference period, IA, 64 FIs (20 FGs)
Barchitta, 2018 (67)<br>Catania (Sicily)<br>Good quality	Cross-sectional study; women diagnosed with an abnormal PAP test without previous treatments and referred to a cervical cancer screening unit in Catania, later classified according to hrHPV status and histological grade of CIN (from normal cervical epithelium to CIN3); recruitment from 2013 to 2015; single center/area	539 total subjects (100% Fs) of which 252 with normal cervical epithelium and 160 CIN1 (i.e., low-grade CIN); 84 hrHPV infections (+) (mean: 38.63 ys, SD: 10.53 ys) among the 251 (as reported in the text) with a normal cervical epithelium; 167 hrHPV infections (-) (mean: 43.65 ys, SD: 9.62 ys) among the 251 (as reported in the text) with a normal cervical epithelium; 127 CIN2+ (mean: 36.01 ys, SD: 8.10 ys); 411 with normal cervical epithelium or CIN1 (as reported in the text) (mean: 41.50 ys, SD: 10.21 ys)	FFQ<br>1 mo before<br>IA<br>Validated<br>95 FIs (39 FGs)
Barchitta, 2019 (68)<br>Catania (Sicily)<br>Good quality	Cross-sectional study; non-pregnant women with no history of severe diseases referring for routine physical examination to three clinical laboratories in Catania; recruitment from 2010 to 2017; single center/area	349 total subjects (100% Fs); age 12-87 ys (median: 36 ys, NA)	FFQ<br>1 mo before<br>IA<br>Adapted from a previously validated FFQ<br>95 FIs (39 FGs)
Barchitta, 2019 (69)<br>Eastern Sicily<br>Fair quality	Cross-sectional study; adolescents attending three high schools in the urban area of Eastern Sicily; single center/area	213 total subjects; age 15-18 ys (median: 16 ys; IQR: 0 ys); 102 Ms (median: 16 ys, IQR: 0 ys), 111 Fs (median: 16 ys, IQR: 1 y)	FFQ<br>NA<br>SA<br>Adapted from a previously validated FFQ<br>95 FIs (36 FGs)

Fernández-Alvira, 2014 (70)<br>Italy, Estonia, Cyprus, Belgium, Sweden, Hungary, Germany, and Spain<br>IDEFICS<br>Good quality	Cross-sectional analysis nested within a prospective cohort study of children aged 2–9 ys from 8 European countries (recruited between September 2007 and May 2008) with the aim to investigate the etiology of obesity and the possible interventions for its prevention; international	14233 total subjects (8028 Ms, 6205 Fs; 2-9 ys, of which 12462 with complete dietary and socioeconomic information; mean: 6.0 ys, SD: 1.8 ys at baseline); Italy 2110 subjects (NA Ms, NA Fs)	Same FFQ across all centers (Children’s Eating Habits Questionnaire-FFQ)<br>1 mo before<br>IA<br>Reproducible and valid<br>43 FIs (14 FGs) to investigate the consumption frequency of obesity-related foods
Naska, 2006 (71)<br>Belgium, France, Finland, Germany, Greece, Italy, Norway, Portugal, Spain, UK<br>DAFNE<br>Fair quality	Analysis of standardized and post-harmonized data collected through the national household budget surveys undertaken in 10 European countries during the 1990s (Italy 1996) on food, goods, and services available to household members during the reference period conducted by the National Statistical Offices of each country; international	94564 original subjects (NA Ms, NA Fs), age from 0 to over 75 ys, of which 15251 were excluded because they did not fit the pre-defined categories; Italy: 22740 original subjects (NA Ms, NA Fs) of which 16% (3638 subjects) was excluded	No dietary assessment tool used; collected data were availability of foods and beverages at the household level taking into consideration the households' purchases, contributions from all production and food items offered to members as gifts; 56 detailed original FGs further aggregated into 25 final FGs
Bravi, 2021 (72)<br>Turin (Piemonte), Florence (Tuscany), Rome (Lazio), San Giovanni Rotondo (Apulia), Palermo (Sicily)<br>MEDIDIET<br>Fair quality	Cross-sectional study; exclusively breastfeeding and healthy women recruited in 5 hospital settings in northern, central and southern Italy had information on dietary habits and a sample of freshly expressed foremilk collected at 6±1 wks post-partum; recruitment between 2012 and 2014; Italian multicentric	300 total subjects (100% Fs), age 25-41 ys (mean: 33 ys, SD: 4.06 ys)	FFQ at 6±1 wks post-partum, same d of milk collection<br>From partum to d of milk collection<br>IA<br>Reproducible and valid<br>78 FIs (31 NUTs)
Lasalvia, 2021 (73)<br>Varese (Lombardy)<br>ROCAV<br>Good quality	Cross-sectional study; men and women randomly selected among residents of the Varese city (Lombardy) without main chronic diseases with the aim to investigate the relation between dietary patterns and arterial stiffness; recruitment between 2013 and 2016; single center/area	2640 total subjects (mean: 65.5 ys, SD: 6.7 ys); 1608 Ms (50-75 ys), 1032 Fs (60-75 ys)	FFQ<br>1 y before<br>SA<br>Reproducible and valid<br>188 FIs (41 FGs)

Zupo, 2020 (74)<br>Castellana Grotte (Apulia)<br>Salus in Apulia Study (from MICOL study)<br>Very good quality	Prospective cohort study originally enrolling participants from Apulia based center of MICOL study in 1985, with a follow-up for mortality until December 31, 2017; single center/area	2472 total subjects (1429 Ms, 1043 Fs); age > 30 ys (mean: 48.00 ys, SD: 10.71 ys) in a representative sample of the population of Apulia in 1985; 990 total deaths, no additional information on causes	FFQ administered in 1985<br>1 y before<br>SA<br>Validated<br>31 FIs (29 FGs)
Tatoli, 2022 (75)<br>Castellana Grotte (Apulia)<br>Salus in Apulia Study (including also a major part of MICOL study participants)<br>Poor quality	Cross-sectional study a part of which nested within the MICOL cohort; investigated dietary differences between subjects with and without diabetes among non-institutionalized older adults from Southern Italy, recruited between 2014 and 2018, based on health registry office list at December 31, 2014, as well as previous MICOL study participants; single center/area	1399 total subjects (mean: 73.43 ys, SD: 6.30 ys); 187 diabetic subjects (115 Ms, 72 Fs; mean: 74.66 ys, SD: 6.39 ys); 1212 non-diabetic subjects (634 Ms, 578 Fs; mean: 73.24 ys, SD: 6.26 ys)	FFQ administered between 2014 and 2018<br>1 y before<br>SA with interviewer checks<br>Validated<br>85 FIs (28 FGs)
Giontella, 2019 (76)<br>Verona (Veneto)<br>Good quality	Cross-sectional study; children were recruited from the third and fourth classes of four primary schools in the Verona South district with the aim to assess the relationship between food, PA, and main CVD risk factors; single center/area	300 total subjects (7-10 ys); 150 Ms (mean: 8.7 ys, SD: 0.8 ys), 150 Fs (mean: 8.6 ys, SD: 0.7 ys)	FFQ<br>NA<br>NA<br>Validated<br>61 FIs (10 FGs)
Turroni, 2021 (77)<br>Emilia-Romagna (Italy)<br>Good quality	Pilot intervention study; based on Istituto Romagnolo per lo Studio dei Tumori "Dino Amadori" (Meldola, Emilia Romagna) recruitment from October 2018 to September 2019; 60 subjects with at least one among abdominal obesity, hypertension, dyslipidemia, impaired fasting glucose or insulin resistance, 33 of which consumed symbiotic agriculture food (SA-group) and 27 of which received probiotic supplementation (PROB-group) over 30 ds, with a follow-up of 15 ds and stool, urine, and blood samples collected over time; single center/area	60 total subjects (13 Ms, 47 Fs) 18.3-86.4 ys (median age at enrollment: 46.9 ys, IQR: NA); 33 subjects in SA-group (5 Ms, 28 Fs) 34.6-86.4 ys (median age at enrollment: 52.7 ys, IQR: NA); 27 subjects in PROB-group (8 Ms, 19 Fs) 18.3-64.2 ys (median age at enrollment: 45.3 ys, IQR: NA)	FFQ<br>1 y before<br>IA<br>Reproducible ad valid<br>188 FIs (27 NUTs)

Donati Zeppa, 2020 (78)<br>Urbino (Marche)<br>Fair quality	Trial; normal-weight M and F young adults were recruited by the University of Urbino to participate to a 9-wk HIIT program to investigate the role of PA in modulating food choices; single center/area	32 total subjects (21-24 ys at enrollment); 20 Ms (mean: 22.6 ys, SD: 1.7 ys), 12 Fs (mean: 21.5 ys, SD: 0.8 ys)	24HR in association with PHOTOdietometer for portion size estimation from 2 wks before to the end of the training session<br>IA<br>NA<br>NA FIs (16 NUTs)
Colica, 2017 (79)<br>Catanzaro (Calabria)<br>Fair quality	Cross-sectional study nested within the cohort reported in Mazza et al. 2017; Caucasian, community-dwelling individuals from Calabria, enrolled between 2013 and 2014, without any bone metabolism disfunctions, aged ≥ 65 ys and satisfying additional criteria underwent whole-body-dual X-ray absorptiometry scan, a fasting venous blood collection, and fractures and dietary intake assessments; single center/area	177 total subjects (37% Ms, 63% Fs); age ≥ 65 ys (mean: 70 ys, SD: 4.1 ys); 41 participants had fractures (52 total fractures)	24HR + 7d-DR<br>NA<br>IA<br>NA<br>NA FIs (10 FGs)
Mazza, 2017 (80)<br>Catanzaro (Calabria)<br>Good quality	Cross-sectional and longitudinal analysis of a prospective cohort enrolled between 2013 and 2014 including community-dwelling, Caucasian individuals from Calabria, aged ≥ 65 ys, who underwent a neuropsychological assessment (MMSE and ADAS-Cog) at baseline and 1-y follow-up, and satisfied additional criteria (e.g., MMSE>20); dietary guidance to promote a "healthy diet" was given by a dietitian to all participants during follow-up; 1-y follow-up; single center/area	214 total subjects ≥ 65 ys at baseline (mean: 70 ys, SD: 4 ys), 144 of which had complete data on ADAS-Cog at follow-up and were included in the follow-up analysis	24HR + 7d-DR at baseline<br>IA<br>Validated<br>NA FIs (8 FGs + 10 NUTs)
Palli, 2001 (81)<br>Florence (Tuscany)<br>Good quality	Case-control study; in high-risk area for gastric cancer in central Italy, 382 cases and 561 controls recruited from 1985 to 1987 and 142 additional controls at the end of the study period to have a more representative sample; population based; single center/area	943 total subjects; 382 cases (239 Ms, 143 Fs) 30 subjects <50 ys, 130 subjects 50-64 ys; 222 subjects >64 ys; 561 controls (328 Ms, 233 Fs) 122 subjects <50 ys, 188 subjects 50-64 ys, 251 subjects >64 ys	FFQ<br>1 y before<br>NA<br>NA<br>181 FIs (20 NUTs)

Anelli, 2022 (82)<br>Milan (Lombardy), Naples (Campania)<br>GIFt Study<br>Very good quality	Prospective cohort study; Italian healthy normal-weight singleton pregnant women at 20±2 gwks recruited between January 2017 and June 2020 in 3 hospital settings in northern and southern Italy, followed-up until delivery for pregnancy outcomes; Italian multicentric	179 total subjects 20-40 ys at baseline (mean: 31.8 ys, SD: 4.3 ys); 85 enrolled in Milan (mean: 31.7 ys, SD: 4.5 ys); 94 enrolled in Naples (mean: 31.9 ys, SD: 4.1 ys);	7d-DR: at 25±1 gwks, IA by a trained dietitian;<br>FFQ: at 29±2 gwks, 3 mos before (second trimester of pregnancy), SA but checked by a trained dietitian, adapted from a previously validated FFQ, 192 FIs (15 FGs)
Ruggieri, 2022 (83)<br>Crotone (Calabria), Milazzo and Augusta-Priolo (Sicily)<br>NEHO Study<br>Good quality	Cross-sectional study nested within a birth cohort; healthy pregnant women with no history of chronic diseases, not requiring special diets, and living in the areas surrounding the perimeter of National Priority Contaminated Sites in Southern Italy were voluntarily recruited starting from January 2018 when admitted to the maternity units of the public hospitals in Milazzo, Syracuse (for the Augusta-Priolo area) and Crotone; Italian multicentric	816 total subjects (100% Fs), age 18-40 ys (mean: 30.6 ys, SD: 5.1 ys); 534 Augusta-Priolo (mean: 30.4 ys, SD ± 5.1 ys); 165 Crotone (mean: 30.5 ys, SD: 5.4 ys); 117 Milazzo (mean: 31.5 ys, SD: 4.5 ys); 589 subjects with available data for risk perception analyses	FFQ<br>Gestational period until FFQ administration (from 32 gwks onwards)<br>IA<br>Not validated<br>41 FIs (38 FGs)

[1]Whenever international studies were included, summarized evidence concerned only the Italian-specific subpopulation and dietary patterns.

ABBREVIATONS: 24HR, 24-hour recall; ADAS-Cog, Alzheimer's Disease Assessment Scale - Cognitive sub-scale; ATBC, Alpha-Tocopherol Beta-Carotene Cancer; CHD, coronary heart disease; CIN, cervical intraepithelial neoplasia; COVID-19, Coronavirus disease 2019; CVD, cardiovascular disease; d, day(s); DAFNE, Data Food Networking; DIETSCAN, Dietary Patterns and Cancer; DP, dietary pattern; DR, dietary record; EI, energy intake(s); EPIC, European Prospective Investigation into Cancer and Nutrition; F, female(s); FFQ, Food Frequency Questionnaire; FG, food group(s); FI, food item(s); GIFt, Gestational Intake of Food towards healthy outcomes; gwk, gestational week(s); HER2, human epidermal growth factor receptor 2; HIIT, high intensity interval training; hrHPV, high-risk Human Papilloma Virus; IA, interviewer administered; IDEFICS, Identification and prevention of Dietary- and lifestyle-induced health EFfects In Children and infantS; IQR, interquartile range; M, male(s); MAMI-MED, Multisettoriale Alla salute Materno-Infantile Mediante valutazione dell'Esposoma nelle Donne; MMSE, Mini Mental State Examination; mo, month(s); NA, not available; NAC-II, Northern Adriatic Cohort II; NEHO, Neonatal Environment and Health Outcomes; NLSC, Netherlands Cohort Study; NUT, nutrient(s); ORDET, Ormoni e Dieta nell'Eziologia del Tumore della Mammella; PA, physical activity; ROCAV, Risk Of Cardiovascular diseases and abdominal aortic Aneurysm in Varese; SA, self-administered; SD, standard deviation; SE, standard error; SMC, Swedish Mammography Cohort; vs., versus; wk, week(s); y, year(s)

Supplemental Table 3. Dietary patterns identified using principal component and factor analyses in Italy[1]

Reference, location, study name, study quality	Dietary pattern identification methods	Expl. Var. % (NF)	Dietary pattern composition
Edefonti, 2008 (34)<br>Breast cancer: northern Italy (Milan, Genoa, Gorizia, Forli), central and southern Italy (Latina, Naples)<br>Ovarian cancer: northern Italy (Milan, Pordenone, Padua), central and southern Italy (Latina, Naples)<br>Good quality	PCFA<br>Standardization<br>EIG>1, Scree plot, and interpretability<br>Varimax rotation<br>|FL|≥0.63<br>Factorability checks NA<br>DP internal consistency NA<br>DP reproducibility (internal)	75.70%<br>(4)	ANIMAL PRODUCTS: animal protein and animal fat, calcium, cholesterol, SFAs, riboflavin, zinc, and phosphorus;<br>VITAMINS AND FIBER: vitamin C and total fiber, total folate, potassium, beta-carotene equivalents, soluble carbohydrates, and vitamin B6;<br>UNSATURATED FAT: vegetable fat and vitamin E, MUFAs and PUFAs;<br>STARCH-RICH: starch, vegetable protein, and sodium
Bertuccio, 2009 (35)<br>Milan (Lombardy)<br>Good quality	PCFA<br>Standardization<br>EIG>1, Scree plot, and interpretability<br>Varimax rotation<br>|FL|≥0.63<br>Factorability checks<br>DP internal consistency<br>DP reproducibility (internal)	75.09%<br>(4)	ANIMAL PRODUCTS: animal protein, riboflavin, cholesterol, phosphorus, calcium, and zinc;<br>VITAMINS AND FIBER: vitamin C, total fiber, potassium, total folate, beta-carotene equivalents, and soluble carbohydrates;<br>VUFA: other PUFAs, vitamin E, MUFAs, LA, and ALA;<br>STARCH-RICH: starch, vegetable protein, and sodium
Edefonti, 2010 (36)<br>Milan (Lombardy), Pordenone (Friuli Venezia Giulia), Rome, Latina (Lazio)<br>Good quality	PCFA<br>Standardization<br>EIG>1, Scree plot, and interpretability<br>Varimax rotation<br>|FL|≥0.63<br>Factorability checks<br>DP internal consistency<br>DP reproducibility (internal)	79.94%<br>(5)	ANIMAL PRODUCTS: animal fat, calcium, SFAs, animal protein, phosphorus, cholesterol, and riboflavin;<br>VITAMINS AND FIBER: vitamin C, total fiber, soluble carbohydrates, and beta-carotene equivalents;<br>UNSATURATED FATS: vegetable fat and vitamin E, MUFAs and PUFAs;<br>RETINOL AND NIACIN: retinol and niacin;<br>STARCH-RICH: starch, vegetable protein, and sodium
Bravi, 2010 (37)<br>Milan (Lombardy); Genoa (Liguria), Pordenone, Gorizia (Friuli Venezia Giulia), Forlì (Emilia-Romagna), Latina (Lazio), Naples (Campania)<br>Good quality	PCFA<br>Standardization<br>EIG>1, Scree plot, and interpretability<br>Varimax rotation<br>|FL|≥0.63<br>Factorability checks<br>DP internal consistency<br>DP reproducibility (internal)	81.36%<br>(5)	ANIMAL PRODUCTS: calcium, animal protein, phosphorus, riboflavin, SFAs, and cholesterol;<br>VITAMINS AND FIBER: vitamin C, total fiber, beta-carotene equivalents, soluble carbohydrates, and total folate;<br>VUFA: LA, ALA, and vitamin E;<br>AUFA: other PUFAs and vitamin D;<br>STARCH-RICH: starch, vegetable protein, and sodium

Edefonti, 2010 (38)<br>Milan (Lombardy),<br>Pordenone (Friuli Venezia Giulia)<br>Good quality	PCFA<br>Standardization<br>EIG>1, Scree plot, and interpretability<br>Varimax rotation<br>|FL|≥0.63<br>Factorability checks<br>DP internal consistency<br>DP reproducibility (internal)	79.00%<br>(5)	ANIMAL PRODUCTS: calcium, phosphorus, riboflavin, animal protein, SFAs, zinc, and cholesterol;<br>VITAMINS AND FIBER: vitamin C and total fiber, beta-carotene equivalents, and total folate;<br>VUFA: LA, ALA, and vitamin E;<br>AUFA: other PUFAs and vitamin D;<br>STARCH-RICH: starch, vegetable protein, and sodium
Bravi, 2012 (39)<br>Milan (Lombardy),<br>Pordenone (Friuli Venezia Giulia); Padua (Veneto)<br>Good quality	PCFA<br>Standardization<br>EIG>1, Scree plot, and interpretability<br>Varimax rotation<br>|FL|≥0.63<br>Factorability checks<br>DP internal consistency<br>DP reproducibility (internal)	79.18%<br>(5)	ANIMAL PRODUCTS AND RELATED COMPONENTS: calcium, phosphorus, riboflavin, animal protein, SFAs, cholesterol, and zinc;<br>VITAMINS AND FIBER: vitamin C, total fiber, beta-carotene equivalents, soluble carbohydrates, and total folate;<br>STARCH-RICH: starch, vegetable protein, and sodium;<br>OTHER PUFAs AND VITAMIN D: other PUFAs, vitamin D, and niacin;<br>OTHER FATS: LA, ALA, and vitamin E
Bosetti, 2013 (40)<br>Milan (Lombardy),<br>Pordenone (Friuli Venezia Giulia)<br>Good quality	PCFA<br>Standardization<br>EIG>1, Scree plot, and interpretability<br>Varimax rotation<br>|FL|≥0.63<br>Factorability checks<br>DP internal consistency<br>DP reproducibility (internal)	75.84%<br>(4)	ANIMAL PRODUCTS: calcium, animal protein, phosphorus, riboflavin, SFAs, cholesterol, and zinc;<br>VITAMINS AND FIBER: vitamin C, total fiber, beta-carotene equivalents, soluble carbohydrates, total folate, and potassium;<br>UNSATURATED FATS: LA, vitamin E, ALA, and other PUFAs;<br>STARCH-RICH: starch, vegetable protein, and sodium
Rosato, 2014 (41)<br>Milan (Lombardy),<br>Pordenone, Gorizia (Friuli Venezia Giulia),<br>Latina (Lazio), Naples (Campania)<br>Good quality	PCFA<br>Standardization<br>EIG>1, Scree plot, and interpretability<br>Varimax rotation<br>|FL|≥0.63<br>Factorability checks<br>DP internal consistency<br>DP reproducibility (internal)	78.27%<br>(5)	ANIMAL PRODUCTS: calcium, phosphorus, riboflavin, animal protein, SFAs, zinc, and cholesterol;<br>VITAMINS AND FIBER: vitamin C, total fiber, beta-carotene equivalents, total folate, and soluble carbohydrates;<br>VUFA: LA, vitamin E, and ALA;<br>AUFA: other PUFAs and vitamin D;<br>STARCH-RICH: starch, vegetable protein, and sodium

Bravi, 2015 (42)<br>Milan (Lombardy),<br>Pordenone, Udine (Friuli Venezia Giulia), Naples (Campania)<br>Good quality	PCFA<br>Standardization<br>EIG>1, Scree plot, and interepretability<br>Varimax rotation<br>|FL|≥0.63<br>Factorability checks<br>DP internal consistency<br>DP reproducibility (internal)	80.04%<br>(5)	WESTERN TYPE DIET: calcium, riboflavin, phosphorus, animal protein, SFAs, cholesterol, and zinc;<br>VITAMINS AND FIBER: vitamin C, total fiber, potassium, total folate, beta-carotene equivalents, and soluble carbohydrates;<br>STARCH-RICH: starch, vegetable protein, and sodium;<br>ANIMAL DERIVED NUTRIENTS AND PUFA: vitamin D, other PUFAs, and niacin;<br>OTHER FATS: LA, ALA, and vitamin E
Edefonti, 2015 (43)<br>Milan (Lombardy),<br>Pordenone (Friuli Venezia Giulia), Naples (Campania), Catania (Sicily)<br>Good quality	PCFA<br>Standardization<br>EIG>1, Scree plot, and interpretability<br>Varimax rotation<br>|FL|≥0.63<br>Factorability checks<br>DP internal consistency<br>DP reproducibility (internal)	79.60%<br>(5)	ANIMAL PRODUCTS: calcium, riboflavin, phosphorus, SFAs, animal protein, and cholesterol;<br>VITAMINS AND FIBER: vitamin C and total fibre, beta-carotene equivalents, and total folate;<br>VUFA: LA, ALA, and vitamin E;<br>AUFA: other PUFAs and vitamin D;<br>STARCH-RICH: starch, vegetable protein, and sodium
Dalmartello, 2020 (44)<br>Milan (Lombardy),<br>Pordenone, Udine (Friuli Venezia Giulia), Latina (Lazio), Naples (Campania)<br>Good quality	PCFA<br>Standardization<br>EIG>1, Scree plot, and interpretability<br>Varimax rotation<br>|FL|≥0.63<br>Factorability checks<br>DP internal consistency<br>DP reproducibility (internal)	74.52%<br>(4)	ANIMAL PRODUCTS: calcium, animal protein, riboflavin, phosphorus, cholesterol, SFAs, and zinc;<br>VITAMINS AND FIBER: vitamin C, total fiber, soluble carbohydrates, beta-carotene equivalents, potassium, and total folate;<br>COOKING OIL AND DRESSING: vitamin E, LA, and ALA;<br>STARCH-RICH: starch, vegetable protein, and sodium
Edefonti, 2020 (45)<br>Milan (Lombardy),<br>Pordenone (Friuli Venezia Giulia), Naples (Campania), Catania (Sicily)<br>Good quality	PCFA<br>Standardization<br>EIG>1, Scree plot, and interpretability<br>Varimax rotation<br>|FL|≥0.63<br>Factorability checks<br>DP internal consistency<br>DP reproducibility (internal)	78.09%<br>(4)	ANIMAL PRODUCTS: calcium, SFAs, riboflavin, animal protein, cholesterol, phosphorus, and zinc;<br>VITAMINS AND FIBER: vitamin C, total fiber, beta-carotene equivalents, vitamin E, potassium, and total folate;<br>AUFA: other PUFAs and vitamin D;<br>STARCH-RICH: starch, vegetable protein, and sodium

Edefonti, 2020 (46)<br>Milan (Lombardy)<br>Good quality	PCFA<br>Standardization<br>EIG>1, Scree plot, and interpretability<br>Varimax rotation<br>|FL|≥0.63<br>Factorability checks<br>DP internal consistency<br>DP reproducibility (internal)	79.85%<br>(5)	ANIMAL PRODUCTS: cholesterol and SFAs;<br>ANTI-OXIDANT VITAMINS AND FIBER: soluble carbohydrates, potassium, vitamin C, vitamin A (Retinol Activity Equivalent), soluble and insoluble fiber, lignans, and flavonoids;<br>VUFA: LA, ALA, and vitamin E;<br>AUFA: EPA and DHA, and vitamin D;<br>STARCH-RICH: total protein, starch, sodium, phosphorus, iron, zinc, magnesium, selenium, and vitamin B1 and B3
Marinoni, 2022 (47)<br>Croatia, Greece, Italy (Friuli Venezia Giulia region)<br>Good quality	PCFA<br>Standardization<br>EIG>1, Scree plot, and interpretability<br>Varimax rotation<br>|FL|≥0.60<br>Factorability checks<br>DP internal consistency<br>DP reproducibility (internal)	63.39%<br>(5)	DAIRY PRODUCTS: calcium, biotin, magnesium, pantothenic acid, iodine, phosphorus, and vitamin B2;<br>PLANT-BASED FOODS: total fiber, vitamin C, folate, potassium, beta-carotene, vitamin E, and iron;<br>FATS: MUFAs, oleic acid, SFAs, and LA;<br>MEAT AND POTATOES: niacin, vitamin B6, proteins, vitamin B1, and zinc;<br>SEAFOOD: EPA, DHA, and selenium
Centritto, 2009 (48)<br>Molise<br>Moli-sani<br>Good quality	PCFA<br>Standardization<br>EIG>1, Scree plot, and interpretability<br>Varimax rotation<br>|FL|≥0.15<br>Factorability checks NA<br>DP internal consistency NA<br>DP reproducibility (internal)	15.7%<br>(3)	OLIVE OIL AND VEGETABLES: olive oil, cooked and raw vegetables, legumes, soups, fruits, fish, potatoes, bouillon, white meat, crustaceans and molluscs, crisp bread and rusks, nuts and dried fruits, yogurt, snacks, and fresh cheese;<br>PASTA AND MEAT: high on pasta and other grains, cooked tomatoes, red meat, white meat, olive oil, animal fats, other sauces, wine, beer, bread, offals, processed meat, and seasoned cheese; low on breakfast cereals and yogurt;<br>EGGS AND SWEETS: eggs, margarines, processed meat, sugar and sweets, vegetable oils, snacks, mayonnaises, butter, seasoned cheese, fresh cheese, pizza, canned fish, fruit juices, coffee, soft drinks, potatoes, white meat, red meat, animal fats, bread, and beer

Bonaccio, 2012 (49)<br>Molise<br>Moli-sani<br>Good quality	PCFA<br>Standardization<br>EIG>1, Scree plot, and interpretability<br>Varimax rotation<br>|FL|≥0.15<br>Factorability checks NA<br>DP internal consistency NA<br>DP reproducibility (internal)	NA%<br>(3)	OLIVE OIL AND VEGETABLES: olive oil, cooked and raw vegetables, legumes, soups, fruits, fish, potatoes, bouillon, white meat, crustaceans and molluscs, crisp bread and rusks, nuts and dried fruits, yogurt, snacks, and fresh cheese;<br>PASTA AND MEAT: high on pasta and other grains, cooked tomatoes, red meat, white meat, olive oil, animal fats, other sauces, wine, beer, bread, offals, processed meat, and seasoned cheese; low on breakfast cereals and yogurt;<br>EGGS AND SWEETS: eggs, margarines, processed meat, sugar and sweets, vegetable oils, snacks, mayonnaises, butter, seasoned cheese, fresh cheese, pizza, canned fish, fruit juices, coffee, soft drinks, potatoes, white meat, red meat, animal fats, bread, and beer
Bonaccio, 2012 (50)<br>Molise<br>Moli-sani<br>Good quality	PCFA<br>Standardization<br>EIG>1, Scree plot, and interpretability<br>Varimax rotation<br>|FL|≥0.15<br>Factorability checks NA<br>DP internal consistency NA<br>DP reproducibility (internal)	NA%<br>(3)	OLIVE OIL AND VEGETABLES: olive oil, cooked and raw vegetables, legumes, soups, fruits, fish, potatoes, bouillon, white meat, crustaceans and molluscs, crisp bread and rusks, nuts and dried fruits, yogurt, snacks, and fresh cheese;<br>PASTA AND MEAT: high on pasta and other grains, cooked tomatoes, red meat, white meat, olive oil, animal fats, other sauces, wine, beer, bread, offals, processed meat, and seasoned cheese; low on breakfast cereals and yogurt;<br>EGGS AND SWEETS: eggs, margarines, processed meat, sugar and sweets, vegetable oils, snacks, mayonnaises, butter, seasoned cheese, fresh cheese, pizza, canned fish, fruit juices, coffee, soft drinks, potatoes, white meat, red meat, animal fats, bread, and beer

Bonaccio, 2013 (51)<br>Molise<br>Moli-sani<br>Good quality	PCFA<br>Standardization<br>EIG>1, Scree plot, and interpretability<br>Varimax rotation<br>|FL|≥0.15<br>Factorability checks NA<br>DP internal consistency NA<br>DP reproducibility (internal)	NA%<br>(3)	OLIVE OIL AND VEGETABLES: olive oil, cooked and raw vegetables, legumes, soups, fruits, fish, potatoes, bouillon, white meat, crustaceans and molluscs, crisp bread and rusks, nuts and dried fruits, yogurt, snacks, and fresh cheese;<br>PASTA AND MEAT: high on pasta and other grains, cooked tomatoes, red meat, white meat, olive oil, animal fats, other sauces, wine, beer, bread, offals, processed meat, and seasoned cheese; low on breakfast cereals and yogurt;<br>EGGS AND SWEETS: eggs, margarines, processed meat, sugar and sweets, vegetable oils, snacks, mayonnaises, butter, seasoned cheese, fresh cheese, pizza, canned fish, fruit juices, coffee, soft drinks, potatoes, white meat, red meat, animal fats, bread, and beer
Bonanni, 2013 (52)<br>Molise<br>Moli-sani<br>Good quality	PCFA<br>Standardization<br>EIG>1, Scree plot, and interpretability<br>Varimax rotation<br>|FL|≥0.15<br>Factorability checks NA<br>DP internal consistency NA<br>DP reproducibility (internal)	NA%<br>(3)	OLIVE OIL AND VEGETABLES: olive oil, cooked and raw vegetables, legumes, soups, fruits, fish, potatoes, bouillon, white meat, crustaceans and molluscs, crisp bread and rusks, nuts and dried fruits, yogurt, snacks, and fresh cheese;<br>PASTA AND MEAT: high on pasta and other grains, cooked tomatoes, red meat, white meat, olive oil, animal fats, other sauces, wine, beer, bread, offals, processed meat, and seasoned cheese; low on breakfast cereals and yogurt;<br>EGGS AND SWEETS: eggs, margarines, processed meat, sugar and sweets, vegetable oils, snacks, mayonnaises, butter, seasoned cheese, fresh cheese, pizza, canned fish, fruit juices, coffee, soft drinks, potatoes, white meat, red meat, animal fats, bread, and beer

Bonaccio, 2013 (53)<br>Molise<br>Moli-sani<br>Good quality	PCFA<br>Standardization<br>EIG>1, Scree plot, and interpretability<br>Varimax rotation<br>|FL|≥0.15<br>Factorability checks NA<br>DP internal consistency NA<br>DP reproducibility (internal)	NA%<br>(3)	OLIVE OIL AND VEGETABLES: olive oil, cooked and raw vegetables, legumes, soups, fruits, fish, potatoes, bouillon, white meat, crustaceans and molluscs, crisp bread and rusks, nuts and dried fruits, yogurt, snacks, and fresh cheese;<br>MEAT AND PASTA: high on pasta and other grains, cooked tomatoes, red meat, white meat, olive oil, animal fats, other sauces, wine, beer, bread, offals, processed meat, and seasoned cheese; low on breakfast cereals and yogurt;<br>EGGS AND SWEETS: eggs, margarines, processed meat, sugar and sweets, vegetable oils, snacks, mayonnaises, butter, seasoned cheese, fresh cheese, pizza, canned fish, fruit juices, coffee, soft drinks, potatoes, white meat, red meat, animal fats, bread, and beer
Bonaccio, 2016 (54)<br>Molise<br>Moli-sani<br>Good quality	PCFA<br>Standardization<br>EIG>1, Scree plot, and interpretability<br>Varimax rotation<br>|FL|≥0.15<br>Factorability checks NA<br>DP internal consistency NA<br>DP reproducibility (internal)	13.5%<br>(3)	OLIVE OIL AND VEGETABLES: olive oil, cooked and raw vegetables, legumes, soups, fruits, fish, potatoes, bouillon, white meat, crustaceans and molluscs, crisp bread and rusks, nuts and dried fruits, yogurt, snacks, and fresh cheese;<br>PASTA AND MEAT: high on pasta and other grains, cooked tomatoes, red meat, white meat, olive oil, animal fats, other sauces, wine, beer, bread, offals, processed meat, and seasoned cheese; low on breakfast cereals and yogurt;<br>EGGS AND SWEETS: eggs, margarines, processed meat, sugar and sweets, vegetable oils, snacks, mayonnaises, butter, seasoned cheese, fresh cheese, pizza, canned fish, fruit juices, coffee, soft drinks, potatoes, white meat, red meat, animal fats, bread, and beer

Bonaccio, 2018 (55)<br>Molise<br>Moli-sani<br>Good quality	PCFA<br>Standardization<br>EIG>1, Scree plot, and interpretability<br>Varimax rotation<br>|FL|≥0.15<br>Factorability checks NA<br>DP internal consistency NA<br>DP reproducibility (internal)	6.6%<br>(3)	OLIVE OIL AND VEGETABLES: olive oil, cooked and raw vegetables, legumes, soups, fruits, fish, potatoes, bouillon, white meat, crustaceans and molluscs, crisp bread and rusks, nuts and dried fruits, yogurt, snacks, and fresh cheese;<br>ANIMAL FATS AND MEAT: high on pasta and other grains, cooked tomatoes, red meat, white meat, olive oil, animal fats, other sauces, wine, beer, bread, offals, processed meat, and seasoned cheese; low on breakfast cereals and yogurt;<br>EGGS AND SWEETS: eggs, margarines, processed meat, sugar and sweets, vegetable oils, snacks, mayonnaises, butter, seasoned cheese, fresh cheese, pizza, canned fish, fruit juices, coffee, soft drinks, potatoes, white meat, red meat, animal fats, bread, and beer
Pala, 2006 (56)<br>Denmark, France, Germany, Greece, Netherlands, Spain, Sweden, UK, Italy (Varese, Turin, Florence, Naples, Ragusa)<br>EPIC (EPIC-Elderly)<br>Good quality	EFA<br>Standardization<br>EIG≥NA, Scree plot<br>Varimax rotation<br>|FL|≥0.30<br>Factorability checks NA<br>DP internal consistency NA<br>DP reproducibility NA	21%<br>(4)	PRUDENT: other vegetables, legumes, cooked leafy vegetables, onions and garlic, cabbage, fish, crustaceans and molluscs, mushrooms, seed oils, cooked tomatoes, fresh fruit (non-citrus), and nuts and seeds;<br>PASTA & MEAT: high on pasta and other grains, beef, other animal fats, cooked tomatoes, wine, bread, processed meat, and pork; low on yogurt;<br>OLIVE OIL & SALAD: olive oil, raw tomatoes, raw leafy vegetables, root vegetables, soup, and chicken and turkey;<br>SWEET & DAIRY: sugar and honey and jam, ice cream, chocolate-based confectionery, cakes and puddings, coffee, processed meat, eggs, milk, butter, cheese, and patisserie and biscuits

Masala, 2007 (57)<br>Denmark, France, Germany, Greece, Netherlands, Spain, Sweden, UK, Italy (Varese, Turin, Florence, Naples, Ragusa)<br>EPIC (EPIC-Elderly)<br>Very good quality	EFA<br>Standardization NA<br>EIG≥NA, Scree plot<br>Varimax rotation<br>|FL|≥0.30<br>Factorability checks NA<br>DP internal consistency NA<br>DP reproducibility NA	21%<br>(4)	PRUDENT: other vegetables, legumes, cooked leafy vegetables, onions and garlic, cabbage, fish, crustaceans and molluscs, mushrooms, seed oil, fresh fruit (non-citrus), cooked tomatoes, and nuts and seeds;<br>PASTA & MEAT: high on pasta and other grains, beef, other animal fats, cooked tomatoes, wine, white bread, processed meat, and pork; low on yogurt;<br>OLIVE OIL & SALAD: olive oil, raw tomatoes, raw leafy vegetables, root vegetables, soup, and chicken and turkey;<br>SWEET & DAIRY: sugar and honey and jam, ice cream, chocolate-based confectionery, cakes and puddings, coffee, processed meat, eggs, milk, butter, cheese, and patisserie and biscuits
Jannasch, 2019 (58)<br>Italy, France, Spain, UK, Netherlands, Germany, Sweden, Denmark<br>EPIC-InterAct<br>Good quality	Separate PCFAs on each country<br>Standardization<br>EIG>1, Scree plot, and interpretability<br>Varimax rotation<br>Simplified sum score with different cut-offs for FL values, but final cut-off equal to |0.4| Factorability checks NA<br>DP internal consistency NA<br>DP reproducibility (internal and cross-study)	18.3%<br>(2)	PC1: leafy vegetables, fruiting vegetables, cabbage, other vegetables, legumes, fish, and vegetable oils;<br>PC2: pasta and rice, red meat, processed meat, other fats, and sugar
Balder, 2003 (11)<br>Netherlands, Sweden, Finland, and Italy<br>DIETSCAN Project (NLSC, SMC, ATBC, ORDET)<br>Poor quality	Separate PCFAs on each of the 4 studies (but NLSC separate analyses for Ms and Fs)<br>Standardization<br>EIG>1, Scree plot, and interpretability<br>Varimax rotation<br>|FL|≥0.35<br>Factorability checks NA<br>DP internal consistency NA<br>DP reproducibility (internal and cross-study)	ORDET:<br>28.5%<br>(4)	(SALAD) VEGETABLES: raw leafy vegetables, dressings, tomatoes, oil, and carrots;<br>PORK, PROCESSED MEAT, POTATOES: butter, non-fermented whole milk, pasta, beef, potatoes, processed meat, cakes, and eggs;<br>COOKED VEGETABLES: legumes, cabbages, cooked leafy vegetables, fish, carrots, rice, potatoes, and poultry;<br>ALCOHOL: high on wine and spirits; low on coffee (with milk), non-fermented lowfat milk, cakes, and other fruits

Männistö, 2005 (12)<br>Netherlands, Sweden, and Italy<br>DIETSCAN Project (NLSC, SMC, ATBC, ORDET)<br>Good quality	Separate PCFAs on each of the 3 studies<br>Standardization<br>EIG>1, Scree plot, and interpretability<br>Varimax rotation<br>|FL|≥0.35<br>Factorability checks NA<br>DP internal consistency NA<br>DP reproducibility NA	ORDET:<br>28.5%<br>(2)	VEGETABLES (VEG): raw leafy vegetables, tomatoes, dressings, oil, and carrots;<br>PORK, PROCESSED MEAT, POTATOES (PPP): butter, pasta, potatoes, beef and veal, and processed meat;<br>Plus 2 additional DPs for ORDET (presented in Balder et al.) but not common to other cohorts and therefore not considered here
Sieri, 2004 (59)<br>Varese (Lombardy)<br>ORDET<br>Very good quality	EFA<br>Standardization NA<br>EIG>NA, Scree plot<br>Varimax rotation<br>|FL|>0.25<br>Factorability checks NA<br>DP internal consistency NA<br>DP reproducibility NA	30%<br>(4)	SALAD VEGETABLES: raw and cooked leafy vegetables, mixed vegetables in salad, raw tomatoes, raw carrots, olive oil and other fruiting vegetables;<br>WESTERN: butter, potatoes, other pasta, processed meat, veal, eggs, cakes, beef, seed oils, offal, pork, and cheese;<br>CANTEEN: pasta, cooked tomatoes, olive oil, pulses, other fruiting vegetables, veal, bread and wine;<br>PRUDENT: high on cooked carrots, cooked leafy vegetables, rice, fish, other fruiting vegetables, pulses, poultry, raw carrots, potatoes, yogurt, and olive oil; low on wine and spirits
Sant, 2007 (60)<br>Varese (Lombardy)<br>ORDET<br>Very good quality	EFA<br>Standardization<br>EIG>NA, Scree plot<br>Varimax rotation<br>|FL|>0.25<br>Factorability checks NA<br>DP internal consistency NA<br>DP reproducibility NA	30%<br>(4)	SALAD VEGETABLES: raw and cooked leafy vegetables, mixed vegetables in salad, raw tomatoes, raw carrots, olive oil and other fruiting vegetables;<br>WESTERN: butter, potatoes, other pasta, processed meat, veal, eggs, cakes, beef, seed oils, offal, pork, and cheese;<br>CANTEEN: pasta, cooked tomatoes, olive oil, pulses, other fruiting vegetables, veal, bread and wine;<br>PRUDENT: high on cooked carrots, cooked leafy vegetables, rice, fish, other fruiting vegetables, pulses, poultry, raw carrots, potatoes, yogurt, and olive oil; low on wine and spirits

Menotti, 2012 (61)<br>Italian Rural Areas of Seven Countries Study of Cardiovascular Disease<br>Seven Countries Study<br>Very good quality	PCFA<br>Standardization NA<br>Adjustment by weight<br>EIG>1, Scree plot<br>Varimax rotation<br>|FL|≥0.25<br>Factorability checks NA<br>DP internal consistency NA<br>DP reproducibility (internal) with PCA	≥82%*<br>(3)	FACTOR 1: sugar, milk, meat, fruit, pastries, and cheese;<br>FACTOR 2: bread, cereals, vegetables, fish, potatoes, and oils;<br>FACTOR 3: eggs and alcoholic beverages
Menotti, 2018 (62)<br>Italian Rural Areas of Seven Countries Study of Cardiovascular Disease<br>Seven Countries Study<br>Very good quality	PCA and EFA<br>Standardization NA<br>Energy adjustment (density method)<br>EIG>1, Scree plot<br>Varimax rotation<br>|FL|≥0.30<br>Factorability checks NA<br>DP internal consistency NA<br>DP reproducibility (internal)	≥82%*<br>(3)	FA2 (EFA-based FACTOR2 from Menotti 2012): bread, cereals, vegetables, fish, potatoes, and oils;<br>PCA2 (PCA-based COMPONENT2 from Menotti 2012): bread, cereals, vegetables, fish, potatoes, and oils;<br>Plus 2 additional factors and 2 additional principal components not further investigated due to the lack of association with CHD mortality
Maugeri, 2019 (63)<br>Mamma & Bambino<br>Catania (Sicily)<br>Fair quality	PCFA<br>Standardization<br>Energy adjustment (residual method)<br>EIG>2, Scree plot, and interpretability<br>Varimax rotation<br>|FL|≥0.25<br>Factorability checks NA<br>DP internal consistency NA<br>DP reproducibility NA	15.6%<br>(2)	PRUDENT: potatoes, raw and cooked vegetables, legumes, rice, and soup;<br>WESTERN: red meat, fries, dipping sauces, salty snacks, and alcoholic drinks

Maugeri, 2019 (64)<br>Mamma & Bambino<br>Catania (Sicily)<br>Fair quality	PCFA<br>Standardization<br>Energy adjustment (residual method)<br>EIG>2, Scree plot, and interpretability<br>Varimax rotation<br>|FL|≥0.20<br>Factorability checks NA<br>DP internal consistency NA<br>DP reproducibility (internal)	15.55%<br>(2)	PRUDENT: potatoes, cooked vegetables, legumes, pizza, and soup;<br>WESTERN: red meat, fries, dipping sauces, salty snacks, and alcoholic drinks
Magnano San Lio, 2022 (65)<br>Catania (Sicily)<br>Good quality	PCFA on the overall sample<br>Standardization<br>Energy adjustment, NA method<br>EIG>2, Scree plot, and interpretability<br>Varimax rotation<br>|FL|≥0.4<br>Factorability checks NA<br>DP internal consistency NA<br>DP reproducibility NA	15.6%<br>(2)	PRUDENT: cooked and raw vegetables, legumes, fruits, fish, and soup;<br>WESTERN: white bread, vegetable oil, fries, salty snacks, dipping sauces, and sweets
Ojeda-Granados, 2022 (66)<br>Catania (Sicily), Guadalajara (Mexico)<br>Fair quality	Separate PCFAs on each country<br>Standardization<br>Energy adjustment (residual method)<br>EIG>2, Scree plot, and interpretability<br>Varimax rotation<br>|FL|≥0.2<br>Factorability checks NA<br>DP internal consistency NA<br>DP reproducibility NA	15.3%<br>(2)	LEGUMES, VEGETABLES AND FISH (DP1): legumes, cooked and raw vegetables, vegetable soup, potatoes, and fish;<br>SNACK FOODS, PROCESSED MEATS AND OILS (DP2): chips, dipping sauces, snacks, processed meat, vegetable oils, red meat, sugar and sweets, and fruit juice

Barchitta, 2018 (67)<br>Catania (Sicily)<br>Good quality	PCFA<br>Standardization<br>Energy adjustment (residual method)<br>EIG>2, Scree plot, and interpretability<br>Varimax rotation<br>|FL|≥0.2<br>Factorability checks NA<br>DP internal consistency NA<br>DP reproducibility (internal)	14.31%<br>(2)	PRUDENT: legumes, vegetable soups, potatoes, cooked and raw vegetables, and olive oil;<br>WESTERN: high on chips, snacks, dipping sauces, plant oils, processed and red meats; low on olive oil
Barchitta, 2019 (68)<br>Catania (Sicily)<br>Good quality	PCFA<br>Standardization<br>Energy adjustment (residual method)<br>EIG>2, Scree plot, and interpretability<br>Varimax rotation<br>|FL|≥0.3<br>Factorability checks NA<br>DP internal consistency NA<br>DP reproducibility NA	17.2%<br>(2)	PRUDENT: potatoes, cooked and raw vegetables, legumes, soup, and fish;<br>WESTERN: high on canned fish, vegetable oil, processed meat, salty snacks, alcoholic drinks, and dipping sauces; low on fruits
Barchitta, 2019 (69)<br>Eastern Sicily<br>Fair quality	PCFA<br>Standardization<br>Energy adjustment (residual method)<br>EIG>2, Scree plot, and interpretability<br>Varimax rotation<br>|FL|≥0.2<br>Factorability checks NA<br>DP internal consistency NA<br>DP reproducibility NA	26.8%<br>(3)	PRUDENT: potatoes, cooked vegetables, legumes, fruits, nuts, yogurt, offals, shellfish, and tea;<br>WESTERN: white bread, red and processed meat, shellfish, vegetable oil, dipping sauces, and fries;<br>ENERGY DENSE: yogurt, butter and margarine, sweets and refined sugar, dipping sauces, pizza, and fries

Fernández-Alvira, 2014 (70)<br>Italy, Estonia, Cyprus, Belgium, Sweden, Hungary, Germany, and Spain<br>IDEFICS<br>Good quality	Separate PCFA by center<br>Standardization NA<br>EIG>1, Scree plot<br>Varimax rotation<br>|FL|≥0.3<br>Factorability checks NA<br>DP internal consistency NA<br>DP reproducibility (cross-study)	20.5%<br>(3)	PROCESSED: crisps, corn crisps and popcorn, ketchup, chocolate and candy bars, mayonnaise and mayonnaise-based products, and sweetened drinks;<br>HEALTHY: raw vegetables, cooked vegetables and beans, fresh fruits without added sugar, fresh or frozen fish (not fried), and fresh meat (not fried);<br>SPREADS: reduced-fat products on bread, butter and/or margarine on bread, jam and honey, and chocolate or nut-based spread
Naska, 2006 (71)<br>Belgium, France, Finland, Germany, Greece, Italy, Norway, Portugal, Spain, UK<br>DAFNE<br>Fair quality	Separate PCAs by country on daily individual food availability defined as recorded food quantities divided by the corresponding household values (defined as age and sex specific consumption units calculated on the basis of the respective average energy requirements using energy requirements of males aged 18-29 ys as the reference unit)<br>Standardization<br>Log-transformation of individual food availability relative to the overall average DAFNE food availability (calculated for each FG as unweighted arithmetic mean of the country-specific mean availability values)<br>EIG>1, and interpretability<br>Rotation NA<br>|FL|≥0.2<br>Factorability checks NA<br>DP internal consistency NA<br>DP reproducibility (cross-study)	PC1: 15-20%; PC2: 6-8%<br>(2)	WIDE RANGE: high on fruits, vegetables, cereals, meat, fish, and dairy products;<br>BEVERAGE AND CONVENIENCE: high on beverages (alcoholic and nonalcoholic) and ready-to-eat dishes; low on plant foods and elaborate-to-cook dishes
Bravi, 2021 (72)<br>Turin (Piemonte), Florence (Tuscany), Rome (Lazio), San Giovanni Rotondo (Apulia), Palermo (Sicily)	PCFA<br>Standardization<br>EIG>1, Scree plot, and interpretability<br>Varimax rotation<br>|FL|≥0.63<br>Factorability checks	80.57%<br>(5)	VITAMINS, MINERALS AND FIBERS: fiber, potassium, iron, folate, vitamin C, vitamin E, and beta-carotene equivalents;<br>PROTEINS AND FATTY ACIDS WITH LEGS: animal protein, SFAs, cholesterol, calcium, phosphorus, zinc, and riboflavin;<br>FATTY ACIDS WITH FINS: EPA, DHA, DPA, and vitamin D;<br>FATTY ACIDS WITH LEAVES: MUFAs, LA, ALA, vitamin E,

MEDIDIET<br>Fair quality	DP internal consistency<br>DP reproducibility (internal)		and lycopene;<br>STARCH AND VEGETABLE PROTEINS: starch, vegetable protein, and sodium
Lasalvia, 2021 (73)<br>Varese (Lombardy)<br>ROCAV<br>Good quality	PCFA<br>EIG>1, Scree plot and total variance explained<br>Varimax rotation<br>FL≥0.28 or FL≤-0.15<br>Factorability checks NA<br>DP internal consistency NA<br>DP reproducibility NA	24.35%<br>(4)	WESTERN: high on red meats, animal fats, processed meats, salty biscuits, vegetable oils, mayonnaise and other sauces, spirits, cheeses, eggs, pizza, crustaceans and molluscs, beer and cider, offals, wine, soft drinks, sugar and sweets, and butter; low on toasted bread and rusks, and fruits;<br>MEDITERRANEAN: high on olive oil, cooked vegetables, raw vegetables, legumes, pasta and other grains, bouillon, cooked tomatoes, soups, fruits, fish, and potatoes; low on soft drinks;<br>CARBOHYDRATE: high on pasta and other grains, cooked tomatoes, bread, and animal fats; low on yogurt, fish, nuts and seeds, breakfast cereals, crustaceans and molluscs, tea, cooked vegetables, fruit juices, fruits, snacks, and eggs;<br>RESIDUAL: high on milk, coffee, and white meats; low on tea, wine, spirits
Zupo, 2020 (74)<br>Castellana Grotte (Apulia)<br>Apulia (from MICOL Study)<br>Very good quality	PCA<br>Percentage of explained variance<br>Varimax rotation NA<br>Descriptive labelling<br>Factorability checks NA<br>DP internal consistency NA<br>DP reproducibility NA	NA%<br>(5)	ENERGY-RICH: cured meat, sausages, lean ham, bacon, desserts, chocolate, and packaged/fried foods;<br>FARM-HOUSE DIET: dairy products, vegetables, legumes, fruits, and semolina-type bread;<br>SWEETS: desserts, chocolate, and package products;<br>WINTER PATTERN: whole grains, poultry, fish, seafood, and legumes;<br>ELDERLY PATTERN: whole milk, semolina-type bread, legumes, and vegetables

Tatoli, 2022 (75)<br>Castellana Grotte (Apulia)<br>Apulia (including also a major part of MICOL Study participants)<br>Poor quality	Separate PCAs by diabetic status<br>Standardization NA<br>Subjective criteria (higher loadings in each group)<br>Varimax rotation NA<br>|FL|≥0.1<br>Factorability checks NA<br>DP internal consistency NA<br>DP reproducibility NA	NA%<br>(1 for each separate PCA)	DIABETIC/VEGETARIAN: dairy products, eggs, vegetables, nuts, legumes, potatoes, olive oil, fruits, sweets, and sugary foods;<br>NOT DIABETIC: white, red and processed meat, seafood, grains, sweets, sugary foods, caloric drinks, ready-to-eat dishes, wine, beer, and spirits
Giontella, 2019 (76)<br>Verona (Veneto)<br>Good quality	PCA<br>Standardization<br>EIG>1, Scree plot NA<br>Varimax rotation<br>|FL|≥0.2<br>Factorability checks<br>DP internal consistency NA<br>DP reproducibility NA	45.5%<br>(2)	HEALTHY: vegetables, fresh and dried fruit, legumes, fish, dairy products, cereals and tubers, eggs, and meat;<br>UNHEALTHY: meat, fast food, sweets, cereals and tubers, eggs, fish, and dairy products
Turroni, 2021 (77)<br>Emilia-Romagna (Italy)<br>Good quality	PCFA<br>Standardization<br>EIG>1, Scree plot, and interpretability<br>Varimax rotation<br>|FL|≥0.63<br>Factorability checks<br>DP internal consistency NA<br>DP reproducibility NA	80.36%<br>(3)	ANIMAL PRODUCTS: animal protein, cholesterol, niacin, zinc, SFAs, phosphorus, vitamin D, sodium, vitamin B6, retinol, riboflavin, thiamin, calcium, and LA;<br>VITAMINS AND FIBER: vitamin C, beta-carotene, total fiber, total folate, vitamin E, potassium, MUFAs, and soluble carbohydrates;<br>REGIONAL: vegetable protein, other PUFAs, and starch
Donati Zeppa, 2020 (78)<br>Urbino (Marche)<br>Fair quality	Principal Axis Factor Analysis<br>Standardization NA<br>Variables are expressed in terms of difference between values at time 3 (mean of the third mesocycle of training) and values at time 0 (mean of the 2 wks-before period)<br>EIG ≥ 1, variance explained<br>Descriptive labelling<br>Factorability checks<br>DP internal consistency NA<br>DP reproducibility NA	71.61%<br>(3)	FACTOR 1: fat, protein, carbohydrate, energy, MUFAs, SFAs, and vitamin E;<br>FACTOR 2: PUFAs, omega 6, and omega 3;<br>FACTOR 3: soluble fiber, insoluble fiber, vitamin C, vitamin A, starch, and iron

Colica, 2017 (79)<br>Catanzaro (Calabria)<br>Fair quality	PCA (not clear which dietary assessment tool is used)<br>Standardization<br>EIG≥1, Scree plot<br>Varimax rotation<br>|FL|>0.4<br>Factorability checks NA<br>DP internal consistency NA<br>DP reproducibility NA	55%<br>(6)	PATTERN 1: meat, grains, olive oil, and potatoes;<br>PATTERN 2: fish, vegetables, and milk;<br>PATTERN 3: cheese, cakes, and fruit;<br>PATTERN 4: cheese and animal-based fats;<br>PATTERN 5: eggs, legumes, and wine;<br>PATTERN 6: cakes, biscuits, and sugary drinks
Mazza, 2017 (80)<br>Catanzaro (Calabria)<br>Good quality	Separate PCA on FGs and NUTs (not clear which dietary assessment tool is used)<br>Standardization<br>EIG≥1, Scree plot<br>Varimax rotation<br>|FL|>0.40<br>Factorability checks (authors' information: not reported in the paper)<br>DP internal consistency NA<br>DP reproducibility NA	NA%<br>(4+4)	FOOD-BASED PATTERNS:<br>CEREALS/MEAT/FISH/OLIVE OIL PATTERN: cereals, meat, fish, and olive oil;<br>CAKES/FRUIT PATTERN: cakes and fruit;<br>ANIMAL FATS/MARGARINES PATTERN: animal fats and margarines;<br>LEGUMES PATTERN: legumes;<br>NUTRIENT-BASED PATTERNS:<br>ANIMAL PROTEIN PATTERN: animal protein;<br>VEGETAL OILS PATTERN: vegetal oils;<br>FATS PATTERN: fats;PLANT PROTEINS/POLYUNSATURATED FATS PATTERN: plant proteins, PUFAs
Palli, 2001 (81)<br>Florence (Tuscany)<br>Good quality	EFA<br>Energy adjustment (residual method)<br>EIG>NA, Scree plot NA, interpretability NA<br>Varimax rotation<br>|FL|≥0.40<br>Factorability checks NA<br>DP internal consistency NA<br>DP reproducibility NA	75.3%<br>(4)	VITAMIN-RICH: sugar, fiber, vitamin C, vitamin E, beta-carotene, and nitrates;<br>TRADITIONAL: total protein, starch, alcohol, nitrite, and N-nitrosodimethylamine;<br>REFINED: total protein, SFAs, other PUFAs, cholesterol, sugar, retinol, vitamin E, vitamin D, and N-nitrosodimethylamine;<br>FAT-RICH: SFA, oleic acid, MUFAs, LA, ALA, cholesterol, and vitamin E

Anelli, 2022 (82)<br>Milan (Lombardy), Naples (Campania)<br>GIFt Study<br>Very good quality	PCA on the overall sample<br>Energy adjustment (NA method) on FGs from FFQ<br>EIG≥1.1, Scree plot NA<br>Rotation NA<br>Descriptive labelling<br>Factorability checks NA<br>DP internal consistency NA<br>DP reproducibility NA	33,4%<br>(3)	HIGH MEAT, ANIMAL FATS, GRAIN: meat, animal fats, and grains;<br>HIGH FISH, FRUIT, NUTS: fish, fruit, and nuts;<br>HIGH EGGS AND SWEETS, LOW LEGUMES: high on eggs and sweets; low on legumes
Ruggieri, 2022 (83)<br>Crotone (Calabria), Milazzo and Augusta-Priolo (Sicily)<br>NEHO Study<br>Good quality	PCA<br>Standardization NA<br>EIG>NA, Scree plot NA, interpretability NA<br>Rotation NA<br>Descriptive labelling<br>Factorability checks NA<br>DP internal consistency NA<br>DP reproducibility NA	24.9%<br>(3)	PRUDENT: stem-leafy-cooked-raw vegetables, cauliflower, blue fish, fresh caught-farmed fish, fruit, legumes, beef, and yogurt;<br>HIGH ENERGY: high on salty snacks, bakery products, cold meats, fries, mayonnaise, soft drinks, bread, butter, pasta, fresh and aged cheese, potatoes, and pork; low on cereals;<br>VEGETARIAN: high leafy-cooked-raw vegetables, lamb and mutton, tubers, fries, cereals, dried fruit, eggs, oil, butter, and potatoes; low on beef and fresh farmed fish

[1]Whenever international studies were included, summarized evidence concerned only the Italian-specific subpopulation and dietary patterns.
ABBREVIATIONS: ALA, alpha-linolenic acid; ATBC, Alpha-Tocopherol Beta-Carotene; AUFA, Animal Unsaturated Fatty Acids; CHD, coronary heart disease; DAFNE, Data Food Networking; DHA, docosahexaenoic acid; DIETSCAN, Dietary Patterns and Cancer; DP, dietary pattern; DPA, docosapentaenoic acid; EFA, Exploratory Factor Analysis; EI, energy intake(s); EIG, eigenvalue; EPA, eicosapentaenoic acid; EPIC, European Prospective Investigation into Cancer and Nutrition; F, female(s); FA, fatty acid(s); FG, food group(s); FL, factor loading(s); GIFt, Gestational Intake of Food towards healthy outcomes;IDEFICS, Identification and prevention of Dietary- and lifestyle-induced health EFfects In Children and infantS; LA, linoleic acid; M, male(s); MICOL, Multicenter Italian Study on Epidemiology of Cholelithiasis; MUFA, monounsaturated fatty acid(s); NA, not available; NEHO, Neonatal Environment and Health Outcomes; NLSC, Netherlands Cohort Study; NUT, nutrient(s); ORDET, Ormoni e Dieta nell'Eziologia del Tumore della Mammella; PC, principal component; PCA, Principal Component Analysis; PCFA, Principal Component Factor Analysis; PUFA, polyunsaturated fatty acid(s); ROCAV, Risk Of Cardiovascular diseases and abdominal aortic Aneurysm in Varese; SFA, saturated fatty acid(s); SMC, Swedish Mammography Cohort; VUFA, Vegetable Unsaturated Fatty Acids; wk, week(s); y, year(s)

Supplemental Table 4. Quantitative assessment of dietary pattern reproducibility for those dietary patterns identified on the same list of input variables: details of study design, participants, dietary assessment tool, and dietary pattern identification method for the papers included in this analysis

Multi-centric case-control studies on diet and cancer at several sites, papers presenting the same list of 28 nutrients as input variables (35, 37-45)

Paper	Study design	Participants	Dietary questionnaire	Dietary pattern identification methods
Bertuccio<br>2009<br>(35)	Case-control study; gastric cancer; hospital based; single center/area	777 total subjects recruited from 1997 to 2007; cases were 230 subjects with incident, histologically confirmed gastric cancer diagnosed no longer than 1 y before the interview, and with no previous diagnosis of cancer; controls were 547 subjects frequency matched to cases by age and sex	FFQ<br>2 ys before<br>IA<br>reproducible and valid<br>78 FIs (28 NUTs)	PCFA<br>Standardization<br>EIG>1, Scree plot, and interpretability<br>Varimax rotation<br>|FL|≥0.63<br>Factorability checks<br>DP internal consistency<br>DP reproducibility (internal)
Bravi<br>2010<br>(37)	Case-control study; colorectal cancer; hospital based; Italian multicentric	6107 total subjects recruited from 1992 to 1996; cases were 1225 subjects with colon cancer and 728 subjects with rectum cancer with histologically confirmed incident diagnosis; controls were 4154 subjects frequency matched to cases by age and sex	FFQ<br>2 ys before<br>IA<br>reproducible and valid<br>78 FIs (28 NUTs)	PCFA<br>Standardization<br>EIG>1, Scree plot, and interpretability<br>Varimax rotation<br>|FL|≥0.63<br>Factorability checks<br>DP internal consistency<br>DP reproducibility (internal)
Edefonti<br>2010<br>(38)	Case-control study; laryngeal cancer; hospital based; Italian multicentric	1548 total subjects recruited from 1992 to 2000; cases were 460 subjects with incident, histologically confirmed squamous cell cancer of the larynx diagnosed no longer than 1 year before the interview and with no history of cancer; controls were 1088 subjects frequency matched with cases by age, sex, and area of residence	FFQ<br>2 ys before<br>IA<br>reproducible and valid<br>78 FIs (28 NUTs)	PCFA<br>Standardization<br>EIG>1, Scree plot, and interpretability<br>Varimax rotation<br>|FL|≥0.63<br>Factorability checks<br>DP internal consistency<br>DP reproducibility (internal)

Bravi<br>2012<br>(39)	Case-control study;<br>esophageal cancer; hospital<br>based; Italian multicentric	1047 total subjects recruited from 1992 to 1997; cases were 304 with incident, histologically confirmed squamous cell cancer of the esophagus, and with no history of cancer; controls were 743 subjects frequency matched with cases by age, sex, period of interview, and area of residence	FFQ<br>2 ys before<br>IA<br>reproducible and valid<br>78 FIs (28 NUTs)	PCFA<br>Standardization<br>EIG>1, Scree plot, and interpretability<br>Varimax rotation<br>|FL|≥0.63<br>Factorability checks<br>DP internal consistency<br>DP reproducibility (internal)
Bosetti<br>2013<br>(40)	Case-control study; pancreatic cancer; hospital based; Italian multicentric	978 total subjects recruited from 1991 to 2008; cases were 326 subjects with incident, confirmed pancreatic cancer; controls were 652 subjects frequency matched to cases by study center, gender, and age	FFQ<br>2 ys before<br>IA<br>reproducible and valid<br>78 FIs (28 NUTs)	PCFA<br>Standardization<br>EIG>1, Scree plot, and interpretability<br>Varimax rotation<br>|FL|≥0.63<br>Factorability checks<br>DP internal consistency<br>DP reproducibility (internal)
Rosato<br>2014<br>(41)	Case-control study; prostate cancer; hospital based; Italian multicentric	2745 total subjects recruited from 1991 to 2002; cases were 1294 men with incident, histologically confirmed prostate cancer; controls were 1451 men admitted to the same network of hospitals	FFQ<br>2 ys before<br>IA<br>reproducible and valid<br>78 FIs (28 NUTs)	PCFA<br>Standardization<br>EIG>1, Scree plot, and interpretability<br>Varimax rotation<br>|FL|≥0.63<br>Factorability checks<br>DP internal consistency<br>DP reproducibility (internal)
Bravi<br>2015<br>(42)	Case-control study;<br>endometrial cancer; hospital<br>based; Italian multicentric	1362 total subjects recruited from 1992 to 2006; cases were 454 women with incident, histologically confirmed endometrial cancer; controls were 908 women frequency matched to cases by study center and age	FFQ<br>2 ys before<br>IA<br>reproducible and valid<br>78 FIs (28 NUTs)	PCFA<br>Standardization<br>EIG>1, Scree plot, and interepretability<br>Varimax rotation<br>|FL|≥0.63<br>Factorability checks<br>DP internal consistency<br>DP reproducibility (internal)

Edefonti 2015 (43)	Case-control study; nasopharyngeal cancer; hospital based; Italian multicentric	792 total subjects recruited from 1992 to 2008; cases were 198 subjects with incident, histologically confirmed nasopharyngeal cancer, diagnosed no longer than 1 year before the interview, and with no history of cancer; controls were 594 subjects frequency matched to cases by age, sex, period of interview, and area of residence	FFQ<br>2 ys before<br>IA<br>reproducible and valid<br>78 FIs (28 NUTs)	PCFA<br>Standardization<br>EIG>1, Scree plot, and interpretability<br>Varimax rotation<br>|FL|≥0.63<br>Factorability checks<br>DP internal consistency<br>DP reproducibility (internal)
Dalmartello 2020 (44)	Case-control study; renal cell cancer; hospital based; Italian multicentric	2301 total subjects recruited from 1992 to 2004; cases were 767 subjects with incident, histologically confirmed renal cell cancer; controls were 1534 subjects matched by study center, sex, and quinquennia of age	FFQ<br>2 ys before<br>IA<br>reproducible and valid<br>78 FIs (28 NUTs)	PCFA<br>Standardization<br>EIG>1, Scree plot, and interpretability<br>Varimax rotation<br>|FL|≥0.63<br>Factorability checks<br>DP internal consistency<br>DP reproducibility (internal)
Edefonti 2020 (45)	Case-control study; bladder cancer; hospital based; Italian multicentric	1355 total subjects recruited from 2003 to 2014; cases were 690 subjects with incident urothelial carcinoma of the bladder (almost confirmed by histology or cytology) and with no previous history of other neoplasms; controls were 665 subjects selected among those admitted to the same hospital networks of cases	FFQ<br>2 ys before<br>IA<br>reproducible and valid<br>80 FIs (28 NUTs)	PCFA<br>Standardization<br>EIG>1, Scree plot, and interpretability<br>Varimax rotation<br>|FL|≥0.63<br>Factorability checks<br>DP internal consistency<br>DP reproducibility (internal)

Moli-sani study, papers presenting the same list of 43 food groups as input variables (49-51, 53)

Paper	Study design	Participants	Dietary questionnaire	Dietary pattern identification methods

Bonaccio 2012 (49)	Cross-sectional study; men and women living in Molise randomly recruited from city-hall registries of Molise by using electronically generated numbers; single center/area	13262 total subjects with information on household income and no reported history of cardiovascular disease, cancer or diabetes, from a cohort of 24325 subjects recruited from March 2005 to April 2010 (Moli-sani Project)	Modified version of the reproducible and valid EPIC FFQ to include some typical southern Italy foods<br>NA reference period<br>SA<br>Validated in a different form<br>188 FIs (43 FGs)	PCFA<br>Standardization<br>EIG>1, Scree plot, and interpretability<br>Varimax rotation<br>|FL|≥0.15<br>Factorability checks NA<br>DP internal consistency NA<br>DP reproducibility (internal)
Bonaccio 2012 (50)	Cross-sectional study; men and women living in Molise randomly recruited from city-hall registries of Molise by using electronically generated numbers; single center/area	959 total subjects with information on mass media exposure from a cohort of 1132 subjects recruited from May 2009 to April 2010 (Moli-sani Project)	Modified version of the reproducible and valid EPIC FFQ to include some typical southern Italy foods<br>NA reference period<br>SA<br>Validated in a different form<br>188 FIs (45 FGs based on reference to a previous paper)	PCFA<br>Standardization<br>EIG>1, Scree plot, and interpretability<br>Varimax rotation<br>|FL|≥0.15<br>Factorability checks NA<br>DP internal consistency NA<br>DP reproducibility (internal)
Bonaccio 2013 (51)	Cross-sectional study; men and women living in Molise randomly recruited from city-hall registries of Molise by using electronically generated numbers; single center/area	744 total subjects with nutritional knowledge assessment and available data on diet from a cohort of 1132 subjects recruited from May 2009 to April 2010 (Moli-sani Project	Modified version of the reproducible and valid EPIC FFQ to include some typical southern Italy foods<br>NA reference period<br>SA<br>Validated in a different form<br>188 FIs (43 FGs)	PCFA<br>Standardization<br>EIG>1, Scree plot, and interpretability<br>Varimax rotation<br>|FL|≥0.15<br>Factorability checks NA<br>DP internal consistency NA<br>DP reproducibility (internal)

Bonaccio 2013 (53)	Cross-sectional study; men and women living in Molise randomly recruited from city-hall registries of Molise by using electronically generated numbers; single center/area	16937 total subjects with information on health-related quality of life and dietary habits, and no reported history of cardiovascular disease or cancer quality of life assessment from a cohort 24325 subjects recruited from March 2005 to April 2010 (Moli-sani Project)	Modified version of the reproducible and valid EPIC FFQ to include some typical southern Italy foods<br>NA reference period<br>SA<br>Validated in a different form<br>188 FIs (43 FGs)	PCFA<br>Standardization<br>EIG>1, Scree plot, and interpretability<br>Varimax rotation<br>|FL|≥0.15<br>Factorability checks NA<br>DP internal consistency NA<br>DP reproducibility (internal)

Moli-sani study, papers presenting the same list of 46 food groups as input variables (54, 55)

Paper	Study design	Participants	Dietary questionnaire	Dietary pattern identification methods
Bonaccio 2016 (54)	Prospective cohort study; men and women living in Molise randomly recruited from city-hall registries of Molise by using electronically generated numbers; single center/area	1995 total subjects with type 2 diabetes at time of enrollment, no reported history of cancer, reliable dietary or medical questionnaires and not lost at follow up from a cohort of 24325 subjects recruited from March 2005 to April 2010 (Moli-sani Project)	Modified version of the reproducible and valid EPIC FFQ to include some typical southern Italy foods<br>NA reference period<br>SA<br>Validated in a different form<br>188 FIs (46 FGs)	PCFA<br>Standardization<br>EIG>1, Scree plot, and interpretability<br>Varimax rotation<br>|FL|≥0.15<br>Factorability checks NA<br>DP internal consistency NA<br>DP reproducibility (internal)
Bonaccio 2018 (55)	Cross-sectional study; men and women living in Molise randomly recruited from city-hall registries of Molise by using electronically generated numbers; single center/area	10812 final subjects with complete information on psychological resilience and dietary information, reliable medical or dietary questionnaires, and plausible energy intakes from a random sample of 18680 participants, from an original cohort of 24325 subjects recruited from March 2005 to April 2010 (Moli-sani Project)	Modified version of the reproducible and valid EPIC FFQ to include some typical southern Italy foods<br>NA reference period<br>SA<br>Validated in a different form<br>188 FIs (46 FGs)	PCFA<br>Standardization<br>EIG>1, Scree plot, and interpretability<br>Varimax rotation<br>|FL|≥0.15<br>Factorability checks NA<br>DP internal consistency NA<br>DP reproducibility (internal)

Research group from Sicily, papers presenting the same list of 39 food groups as input variables (66, 67)

Paper	Study design	Participants	Dietary questionnaire	Dietary pattern identification methods

Ojeda-Granados 2022 (66)	Cross-sectional study from Italy and Mexico; international	811 Italian non-pregnant women with no history of severe diseases recruited among those referring to three clinical laboratories in Catania from 2010 to 2017	Italian FFQ:<br>1 mo before<br>IA<br>Adapted from a previously validated FFQ<br>95 FIs (39 FGs)	Separate PCFAs on each country<br>Standardization<br>Energy adjustment (residual method)<br>EIG>2, Scree plot, and interpretability<br>Varimax rotation<br>|FL|≥0.2<br>Factorability checks NA<br>DP internal consistency NA<br>DP reproducibility NA
Barchitta 2018 (67)	Cross-sectional study; single center/area	539 women diagnosed with an abnormal PAP test without previous treatments and referred to a cervical cancer screening unit in Catania, later classified according to hrHPV status and histological grade of CIN (from normal cervical epithelium to CIN3), recruited from 2013 to 2015	FFQ<br>1 mo before<br>IA<br>Validated<br>95 FIs (39 FGs)	PCFA<br>Standardization<br>Energy adjustment (residual method)<br>EIG>2, Scree plot, and interpretability<br>Varimax rotation<br>|FL|≥0.2<br>Factorability checks NA<br>DP internal consistency NA<br>DP reproducibility (internal)

ABBREVIATIONS: CIN, cervical intraepithelial neoplasia; DP, dietary pattern; EIG, eigenvalue; EPIC, European Prospective Investigation into Cancer and Nutrition; FFQ, Food Frequency Questionnaire; FG, food group(s); FI, food item(s); FL, factor loading(s); hrHPV, high-risk Human Papilloma Virus; IA, interviewer administered; NA, not available; NUT, nutrient(s); PCFA, Principal Component Factor Analysis; SA, self-administered

Supplemental Table 5. Factor congruence coefficients[1] between pairs of apparently similar dietary patterns identified in the multicentric case-control studies on diet and cancer at several sites, presenting the same list of 28 nutrients as input variables for dietary pattern computation

Animal Products

	Gastric cancer (35)	Colorectal cancer (37)	Laryngeal cancer (38)	Esophageal cancer (39)	Pancreatic cancer (40)	Prostatic cancer (41)	Endometrial cancer (42)	Nasopharyngeal cancer (43)	Renal cancer (44)	Bladder cancer (45)
Gastric cancer	1.00	**0.95**	**0.96**	**0.97**	**0.98**	**0.96**	**0.96**	**0.96**	**0.99**	**0.96**
Colorectal cancer	-	1.00	**0.99**	**0.99**	**0.99**	**0.99**	**1.00**	**1.00**	**0.98**	**0.98**
Laryngeal cancer	-	-	1.00	**1.00**	**0.99**	**0.99**	**1.00**	**0.99**	**0.98**	**0.97**
Esophageal cancer	-	-	-	1.00	**1.00**	**0.99**	**0.99**	**0.99**	**0.99**	**0.97**
Pancreatic cancer	-	-	-	-	1.00	**0.99**	**0.99**	**0.99**	**0.99**	**0.97**
Prostatic cancer	-	-	-	-	-	1.00	**1.00**	**0.99**	**0.99**	**0.98**
Endometrial cancer	-	-	-	-	-	-	1.00	**0.99**	**0.99**	**0.98**
Nasopharyngeal cancer	-	-	-	-	-	-	-	1.00	**0.98**	**0.98**
Renal cancer	-	-	-	-	-	-	-	-	1.00	**0.98**
Bladder cancer	-	-	-	-	-	-	-	-	-	1.00

Vitamins and Fiber

	Gastric cancer (35)	Colorectal cancer (37)	Laryngeal cancer (38)	Esophageal cancer (39)	Pancreatic cancer (40)	Prostatic cancer (41)	Endometrial cancer (42)	Nasopharyngeal cancer (43)	Renal cancer (44)	Bladder cancer (45)
Gastric cancer	1.00	**0.99**	**0.97**	**0.97**	**0.99**	**0.98**	**0.99**	**0.98**	**0.98**	**0.98**
Colorectal cancer	-	1.00	**0.99**	**0.99**	**0.99**	**0.98**	**0.99**	**0.98**	**0.96**	**0.97**

Laryngeal cancer	-	-	1.00	**0.98**	**0.99**	**0.99**	**0.98**	**0.99**	**0.96**	**0.95**
Esophageal cancer	-	-	-	1.00	**0.98**	**0.98**	**0.97**	**0.97**	**0.96**	**0.97**
Pancreatic cancer	-	-	-	-	1.00	**0.99**	**0.99**	**0.99**	**0.99**	**0.97**
Prostatic cancer	-	-	-	-	-	1.00	**0.99**	**0.99**	**0.97**	**0.95**
Endometrial cancer	-	-	-	-	-	-	1.00	**0.99**	**0.98**	**0.97**
Nasopharyngeal cancer	-	-	-	-	-	-	-	1.00	**0.97**	**0.96**
Renal cancer	-	-	-	-	-	-	-	-	1.00	**0.95**
Bladder cancer	-	-	-	-	-	-	-	-	-	1.00

	Starch-rich									
	Gastric cancer (35)	Colorectal cancer (37)	Laryngeal cancer (38)	Esophageal cancer (39)	Pancreatic cancer (40)	Prostatic cancer (41)	Endometrial cancer (42)	Nasopharyngeal cancer (43)	Renal cancer (44)	Bladder cancer (45)
Gastric cancer	1.00	0.94	0.92	**0.95**	0.90	0.93	**0.96**	0.94	0.88	0.93
Colorectal cancer	-	1.00	**0.99**	**0.99**	**0.99**	**0.99**	**0.99**	**1.00**	**0.98**	0.92
Laryngeal cancer	-	-	1.00	**0.99**	**0.99**	**0.98**	**0.98**	**0.99**	**0.98**	0.90
Esophageal cancer	-	-	-	1.00	**0.98**	**0.98**	**0.99**	**0.99**	**0.96**	0.92
Pancreatic cancer	-	-	-	-	1.00	**0.97**	**0.97**	**0.98**	**0.99**	0.89
Prostatic cancer	-	-	-	-	-	1.00	**0.99**	**0.99**	**0.97**	0.93
Endometrial cancer	-	-	-	-	-	-	1.00	**0.99**	**0.96**	0.94
Nasopharyngeal cancer	-	-	-	-	-	-	-	1.00	**0.98**	0.93
Renal cancer	-	-	-	-	-	-	-	-	1.00	0.88

Bladder cancer	-	-	-	-	-	-	-	-	-	1.00

Animal Unsaturated Fatty Acids (AUFA)[2]

	Colorectal cancer (37)	Laryngeal cancer (38)	Esophageal cancer (39)	Prostatic cancer (41)	Endometrial cancer (42)	Nasopharyngeal cancer (43)	Bladder cancer (45)
Colorectal cancer	1.00	**0.99**	**0.96**	**0.97**	**0.99**	**0.98**	0.92
Laryngeal cancer	-	1.00	**0.97**	**0.99**	**0.99**	**0.98**	0.94
Esophageal cancer	-	-	1.00	**0.96**	**0.97**	**0.96**	**0.96**
Prostatic cancer	-	-	-	1.00	**0.98**	**0.96**	0.91
Endometrial cancer	-	-	-	-	1.00	**0.98**	0.94
Nasopharyngeal cancer	-	-	-	-	-	1.00	0.92
Bladder cancer	-	-	-	-	-	-	1.00

Vegetable Unsaturated Fatty Acids (VUFA)[3]

	Gastric cancer (35)	Colorectal cancer (37)	Laryngeal cancer (38)	Esophageal cancer (39)	Pancreatic cancer (40)	Prostatic cancer (41)	Endometrial cancer (42)	Nasopharyngeal cancer (43)	Renal cancer (44)
Gastric cancer	1.00	0.93	0.92	0.88	**0.98**	0.92	0.93	0.92	**0.98**
Colorectal cancer	-	1.00	**0.99**	**0.97**	0.94	**0.99**	**0.98**	**0.97**	**0.96**
Laryngeal cancer	-	-	1.00	**0.99**	0.93	**0.99**	**0.98**	**0.97**	**0.96**
Esophageal cancer	-	-	-	1.00	0.91	**0.97**	**0.97**	**0.95**	0.94
Pancreatic cancer	-	-	-	-	1.00	0.93	0.93	0.92	**0.99**
Prostatic cancer	-	-	-	-	-	1.00	**0.97**	**0.96**	**0.96**
Endometrial cancer	-	-	-	-	-	-	1.00	**0.97**	**0.96**
Nasopharyngeal cancer	-	-	-	-	-	-	-	1.00	0.94

Renal cancer	-	-	-	-	-	-	-	-	1.00

[1]Congruence coefficients range between 0 and 1 (in absolute value), with values between 0.85 and 0.94 indicating fair similarity and values ≥0.95 (in bold typeface in the upper triangular matrix) indicating equivalence of corresponding dietary patterns.
[2]Three papers (35, 40, 44) are missing as the *AUFA* DP was not identified there.
[3]One paper (45) is missing as the *VUFA* DP was not identified there.

Supplemental Table 6. Factor congruence coefficients[1] between pairs of apparently similar dietary patterns identified in the papers involving the Moli-sani study population for the same list of 43 and 46 food groups as input variables for dietary pattern computation, respectively

**43 food groups**				
Olive Oil and Vegetables				
	Household income (49)	Mass media exposure (50)	Nutrition knowledge (51)	Quality of life (53)
Household income	1.00	0.94	**0.95**	**1.00**
Mass media exposure	-	1.00	**0.99**	**0.95**
Nutrition knowledge	-	-	1.00	**0.95**
Quality of life	-	-	-	1.00
Pasta and Meat				
	Household income (49)	Mass media exposure (50)	Nutrition knowledge (51)	Quality of life (53)
Household income	1.00	**0.97**	**0.97**	**1.00**
Mass media exposure	-	1.00	**0.99**	**0.98**
Nutrition knowledge	-	-	1.00	**0.97**
Quality of life	-	-	-	1.00
Eggs and Sweets				
	Household income (49)	Mass media exposure (50)	Nutrition knowledge (51)	Quality of life (53)
Household income	1.00	0.93	0.92	**1.00**
Mass media exposure	-	1.00	**0.99**	0.94
Nutrition knowledge	-	-	1.00	0.93
Quality of life	-	-	-	1.00

**46 food groups**		
Olive Oil and Vegetables		
	Overall and cause-specific mortality (54)	Cognitive performance (55)
Overall and cause-specific mortality	1.00	**0.98**

Cognitive performance	-	1.00
Pasta and Meat		
	Overall and cause-specific mortality (54)	Cognitive performance (55)
Overall and cause-specific mortality	1.00	**0.98**
Cognitive performance	-	1.00
Eggs and Sweets		
	Overall and cause-specific mortality (54)	Cognitive performance (55)
Overall and cause-specific mortality	1.00	**0.97**
Cognitive performance	-	1.00

[1] Congruence coefficients range between 0 and 1 (in absolute value), with values between 0.85 and 0.94 indicating fair similarity and values ≥0.95 (in bold typeface in the upper triangular matrix) indicating equivalence of corresponding dietary patterns.

Supplemental Figure 1. Summary of quality assessment for studies included in the systematic review by single rating tool available from the National Institutes of Health, National Heart, Lung, and Blood Institute[1]

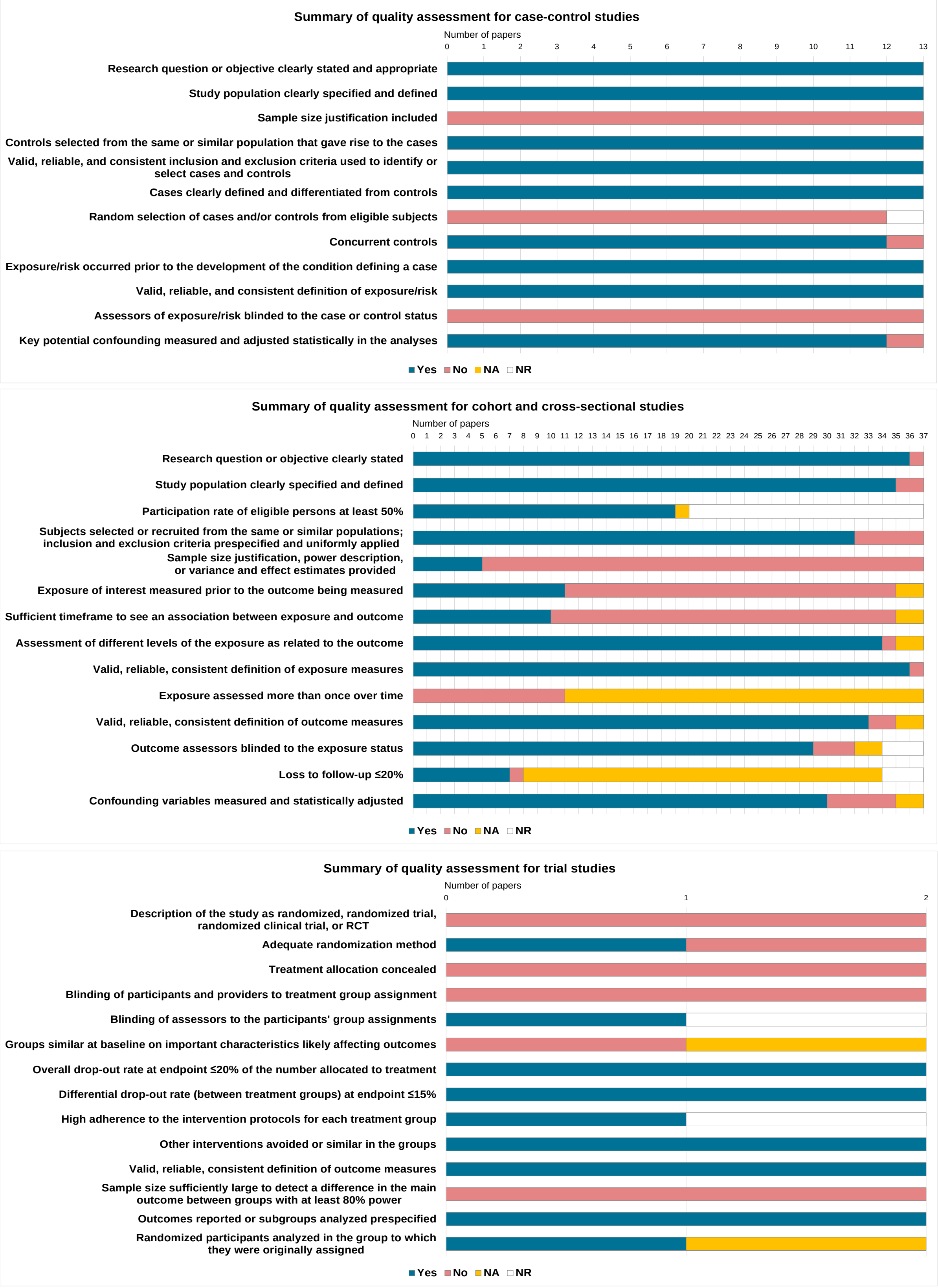

[1]For each quality assessment tool, each row reported the distribution of replies (“Yes”, “No”, “Not applicable”, and “Not reported”) to single questions. The “Cannot determine” reply was never used during this quality assessment.

ABBREVIATIONS: NA, Not Applicable; NR, Not reported
